# A Chemical Biological Approach to Study G Protein-Coupled Receptors: Labeling the Adenosine A_1_ Receptor Using an Electrophilic Covalent Probe

**DOI:** 10.1021/acschembio.2c00589

**Published:** 2022-10-24

**Authors:** Bert L. H. Beerkens, Çağla Koç, Rongfang Liu, Bogdan I. Florea, Sylvia E. Le Dévédec, Laura H. Heitman, Adriaan P. IJzerman, Daan van der Es

**Affiliations:** † Division of Drug Discovery and Safety, Leiden Academic Centre for Drug Research, Leiden University, Einsteinweg 55, 2333 CC Leiden, The Netherlands; ‡ Department of Bioorganic Synthesis, Leiden Institute of Chemistry, Leiden University, Einsteinweg 55, 2333 CC Leiden, The Netherlands; § Oncode Institute, 2333 CC Leiden, The Netherlands

## Abstract

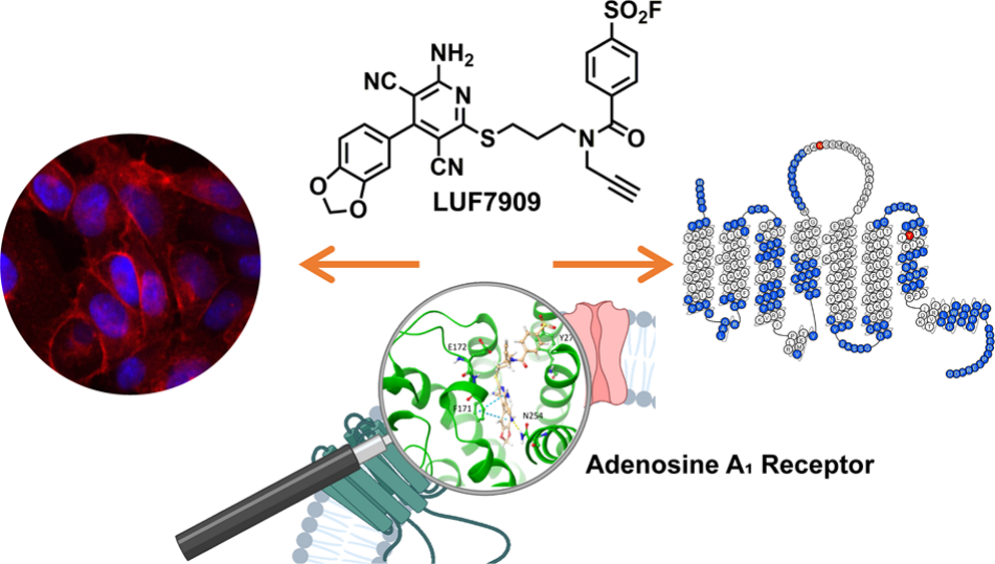

G protein-coupled receptors (GPCRs) have been known for decades as attractive drug targets. This has led to the development and approval of many ligands targeting GPCRs. Although ligand binding effects have been studied thoroughly for many GPCRs, there are multiple aspects of GPCR signaling that remain poorly understood. The reasons for this are the difficulties that are encountered upon studying GPCRs, for example, a poor solubility and low expression levels. In this work, we have managed to overcome some of these issues by developing an affinity-based probe for a prototypic GPCR, the adenosine A_1_ receptor (A_1_AR). Here, we show the design, synthesis, and biological evaluation of this probe in various biochemical assays, such as SDS-PAGE, confocal microscopy, and chemical proteomics.

## Introduction

Adenosine receptors (A_1_, A_2A_, A_2B_, and A_3_) are class A G protein-coupled receptors (GPCRs) that respond to extracellular levels of adenosine.^1,2^ These receptors play a role in a wide variety of physiological and pathological processes, ranging from the suppression of immune responses to the regulation of nociception.^3,4^ This versatile role has prompted decades of research toward the effects of modulating adenosine receptors and led to the development of multiple clinical candidates. However, only few chemical entities have thus far reached the markets.^5,6^

One reason for the lack of success might be the multitasking role of the adenosine receptors throughout the human body; for example, the adenosine A_1_ receptor (A_1_AR) influences lipolysis in adipocytes, reduces ischemic injury in cardiomyocytes, and shows analgesic activity in the spinal cord.^4,7,8^ Other reasons might be receptor oligomerization, biased downstream signaling, and the presence/absence of post-translational modifications (PTMs).^9,10^

The latter observations are not limited to the adenosine receptors but have been found to play a role in the signaling of GPCRs in general.^11,12^ Altogether, there is a plethora of possible mechanisms that could affect targeting of GPCRs in a specific disease state. As GPCRs are the target of roughly one third of the FDA-approved drugs (∼34% in 2017),^13^ it is important to get a better picture of all possible aspects that have an influence on receptor signaling.

**Figure 1:**
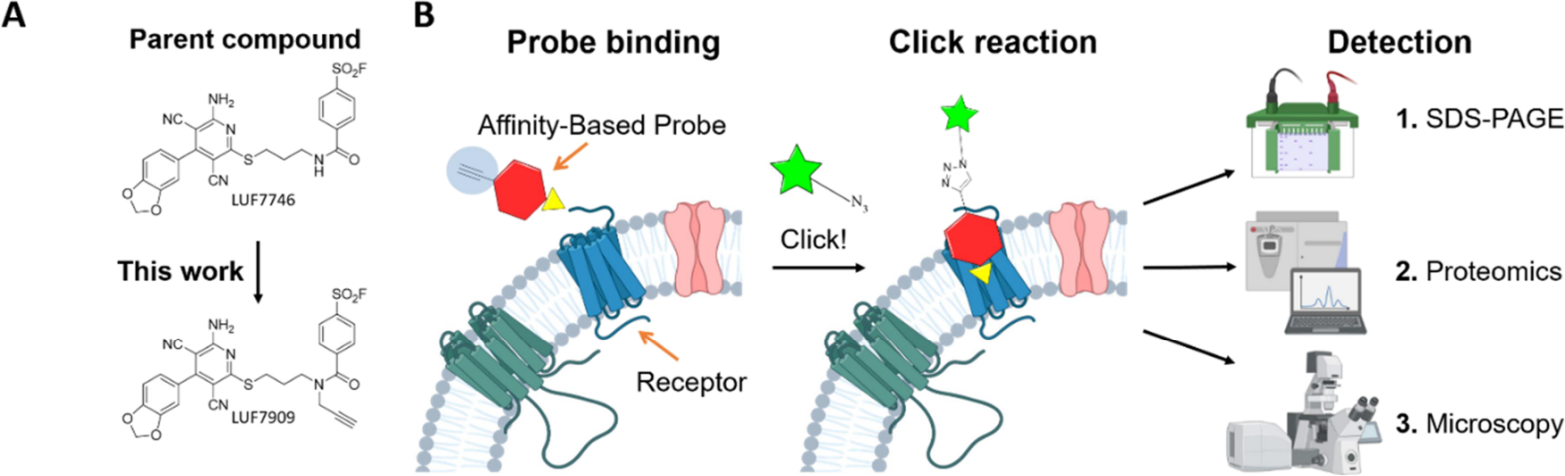
(A) A_1_AR-targeting 3,5-dicyanopyridines: covalent partial agonist LUF7746 and AfBP LUF7909. (B) Typical affinity-based protein profiling workflow used in this study. First, membrane fractions or live cells are incubated with alkyne-containing AfBP. Second, the alkyne moiety of the probe is conjugated to an azide-containing reporter group through the copper(I)-catalyzed azide-alkyne cycloaddition (CuAAC). Third, the probe-bound proteins are being processed for further detection methods. These include SDS-PAGE experiments, chemical proteomics, and confocal microscopy. Figure created with BioRender.com.

Parallel to the development of novel assays and more accurate read-outs in existing assay setups, the development of tool compounds is a valid approach to obtain a better understanding of GPCRs.^14,15^ To this end, our group recently developed LUF7746: a partial agonist for the A_1_AR equipped with an electrophilic fluorosulfonyl group, which facilitated covalent binding to the A_1_AR (Figure 1A).^16^ Covalent ligands for GPCRs have especially proven useful in structural studies, “locking” multiple individual receptors into the same conformation.^17,18^

Aside from covalent ligands, multiple functional ligands have been developed over the years to expand the scope of GPCR profiling.^15^ Among these tool molecules are ligands that are conjugated to, for example, fluorophores, bio-orthogonal click handles, and photoactivatable groups.^19−22^ In other protein families such as hydrolases and proteases, many so-called activity-based probes have already been developed to broadly characterize the respective protein family.^23,24^ Having such an extensive arsenal of probes for GPCRs would allow for a more thorough investigation of these important drug targets in biological systems.

Activity-based probes consist of three parts: a selective targeting moiety, a reactive electrophilic group (warhead), and a reporter tag to detect the probe-bound proteins.^25^ Classical activity-based probes target the active site of an enzyme, using warheads that make use of the enzyme’s intrinsic mechanism to react.^23,24^ GPCRs, on the other hand, do not have such an active site pocket. Therefore, in case of GPCRs, affinity-based probes (AfBPs) have been developed which either use photoactivatable or highly electrophilic groups as warheads.^26−31^ AfBPs thus rely on high affinity and selectivity toward a protein target for selective labeling. Besides that, there are various challenges associated with the biochemical profiling of GPCRs. First, most receptors have low expression levels, even when stably overexpressed in model cell lines.^1,32,33^ Second, as mentioned before, oligomerization and PTMs greatly influence the behavior and appearance of GPCRs.^9,10^ Third, GPCRs are membrane proteins, which are poorly soluble in aqueous media and thus prone to solubility issues.^32−34^

Recently, a handful of AfBPs has been developed to target and label the adenosine receptors. Among these are a clickable antagonist for the A_2A_AR and a clickable antagonist with high affinity for both the A_1_AR and A_3_AR.^29,31^ The application of these probes, however, has been limited to gel-based experiments. Presumably, the aforementioned issues, such as expression levels and the presence of PTMs, have impeded the detection of adenosine receptors in a biochemical setup. Therefore, further exploration of the design, synthesis, and applicability of such probes is warranted.

In this study, we explored another avenue to study the adenosine receptors with AfBPs, enabling the profiling of the A_1_AR in a multitude of biochemical assays. To achieve this, we developed the first agonistic AfBP for the A_1_AR, starting from the aforementioned covalent partial agonist LUF7746. LUF7746 already contains two elements of an AfBP, the only element lacking is the reporter tag for detection.^16^ An alkyne group was chosen as a ligation handle to be conjugated to a reporter tag in the CuAAC.^35,36^ The choice of the alkyne moiety has two advantages compared to a direct conjugation with a reporter group (one-step-probe). First, the alkyne group is a small moiety and thus accounts for minimal steric clashes in the binding pocket of the A_1_AR. Second, having such a ligation handle provides the flexibility to “click” various types of reporter tags onto the probe-bound protein.

In an exemplary GPCR profiling assay, live cells or membrane fractions are first incubated with the probe to selectively label the desired receptor in the presence of other proteins (Figure 1B). In the subsequent incubation step, the desired reporter group is “clicked” onto the probe, effectively labeling the receptor. Finally, the reporter-bound receptor is further processed, depending on the type of detection method. In our case, three different techniques were used to detect the A_1_AR: SDS-PAGE, chemical proteomics, and confocal microscopy. Here, we show that our synthesized probe, LUF7909, was successfully used in all three of these profiling setups. Taken together, this allows us to gain more insight into various receptor properties, such as expression, glycosylation, and the effects of ligand binding.

## Results and Discussion

### Design and Synthesis of A_1_AR-Targeting AfBPs

**Scheme 1:**
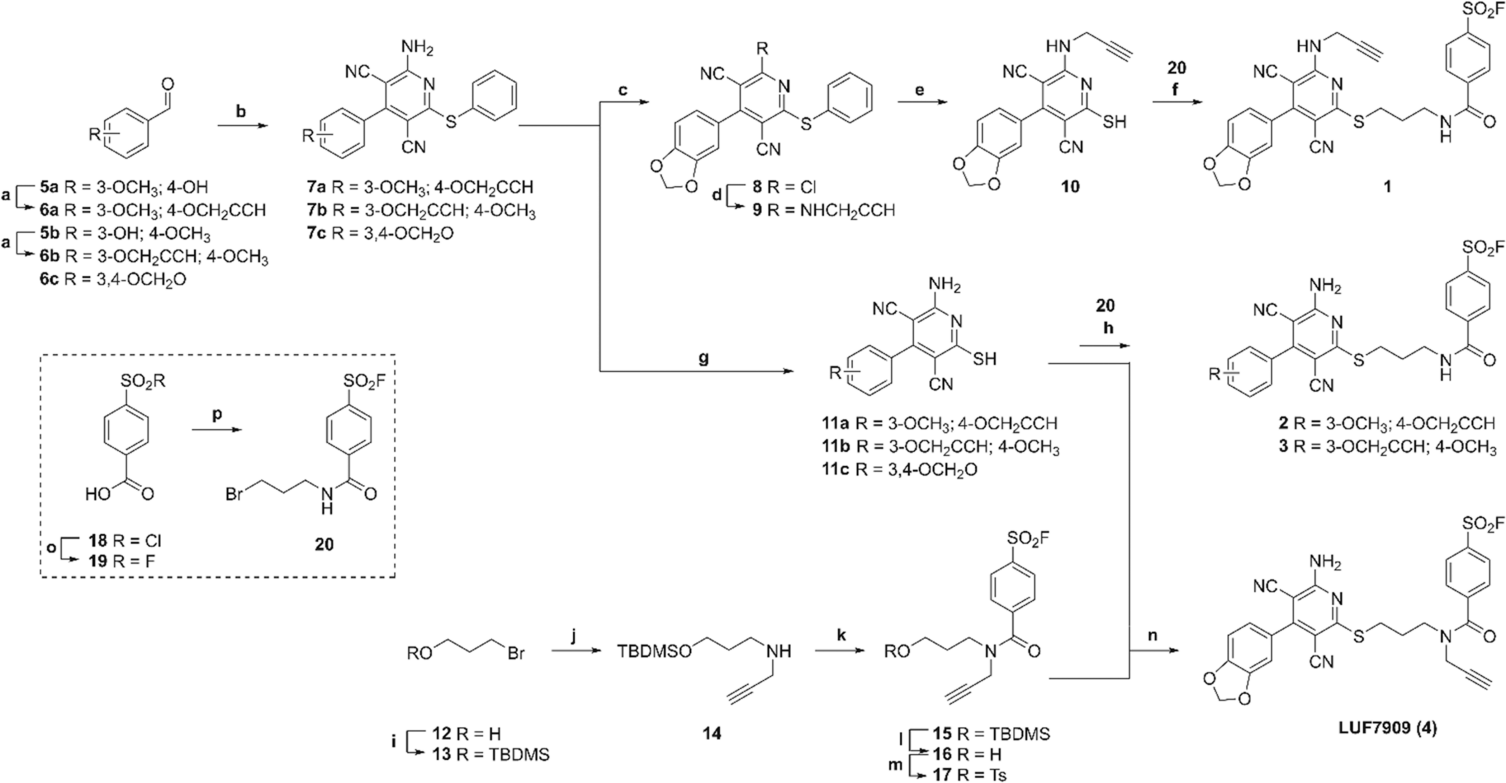
Synthesis of A_1_AR-Targeting AfBPs^a^ Reagents and conditions: (a) Propargyl bromide (80% in toluene), K_2_CO_3_, acetone, reflux, overnight, 80–100%; (b) malononitrile, thiophenol, Et_3_N, EtOH, 50–75 °C, 4–10 h, 30–47%; (c) isopentyl nitrite, CuCl_2_, MeCN, 60 °C, 20 h, 72%; (d) propargyl amine, dry THF, RT, overnight, 92%; (e) potassium thioacetate, dry DMF, RT, 8 h, 99%; (f) **20**, NaHCO_3_, dry DMF, 20–50 °C, 4 days, 40%; (g) and (i) potassium thioacetate, dry DMF, RT, 1–5 days; (ii) 2 M NaOH, RT, 1–3 days, 62–78%; (h) **20**, NaHCO_3_, dry DMF, RT, 1–2 days, 29–53%; (i) TBDMS-Cl (50% in toluene), 1H-imidazole, dry DMF, RT, 5 h, quant.; (j) propargylamine, DIPEA, MeCN, RT, 5 h, 52%; (k) **19**, EDC·HCl, DIPEA, dry DMF, RT, 3 days, 58%; (l) Et_3_N·_3_HF, dry THF, RT, overnight, 92%; (m) TsCl, Et_3_N, dry DMF, RT, 2 days, 55%; (n) NaHCO_3_, dry DMF, RT, overnight, 31%; (o) potassium bifluoride, H_2_O, dioxane, 3 h, 95%; (p) 3-bromopropylamine·HBr, Pybrop, DIPEA, dry DMF, RT, 12 days, 41%.

As mentioned before, our AfBP-design includes an alkyne moiety. To explore the effects that introducing an alkyne has on the binding of LUF7746 to the receptor, multiple ligands were synthesized (i.e., **1**, **2**, **3**, and **4**) each having the alkyne group substituted at a different position of the scaffold (Scheme 1). In all four cases, the synthesis started from the respective benzaldehydes (**6a–c**) which were converted to the corresponding 3,5-dicyanopyridines (**7a–c**) through a multiple component reaction using malononitrile and thiophenol.^37^ 3,5-Dicyanopyridine **7c** was subjected to a Sandmeyer reaction (to furnish chloride **8**), followed by a substitution by propargyl amine (to give alkyne **9**).^8^ The four 3,5-dicyanopyridines (**7a–c** and **9**) were deprotected using thioacetate (**10**, **11a–c**) and subsequently used in a nucleophilic substitution reaction with compound **20** to yield the AfBPs **1**, **2**, and **3**.

In the case of probe **4**, the alkyne group was introduced onto the warhead-containing linker moiety prior to nucleophilic attack of the thiol. In brief, 3-bromopropanol was protected with a TBDMS group (**13**) and converted to compound **14** by a dropwise addition of propargyl amine. 4-Fluorosulfonyl benzoic acid (**19**) was coupled to the same amine in a peptide coupling (**15**) followed by a TBDMS-deprotection (**16**) and a tosylation (**17**) of the compound. The tosylate **20** was then used in the substitution reaction, yielding AfBP LUF7909 (**4**) as a mixture of two rotamers, as determined by nuclear magnetic resonance (NMR) and liquid chromatography–mass spectrometry (LC–MS) measurements.

### Evaluation of the AfBPs in Radioligand Binding Assays and Docking Studies

**Figure 2:**
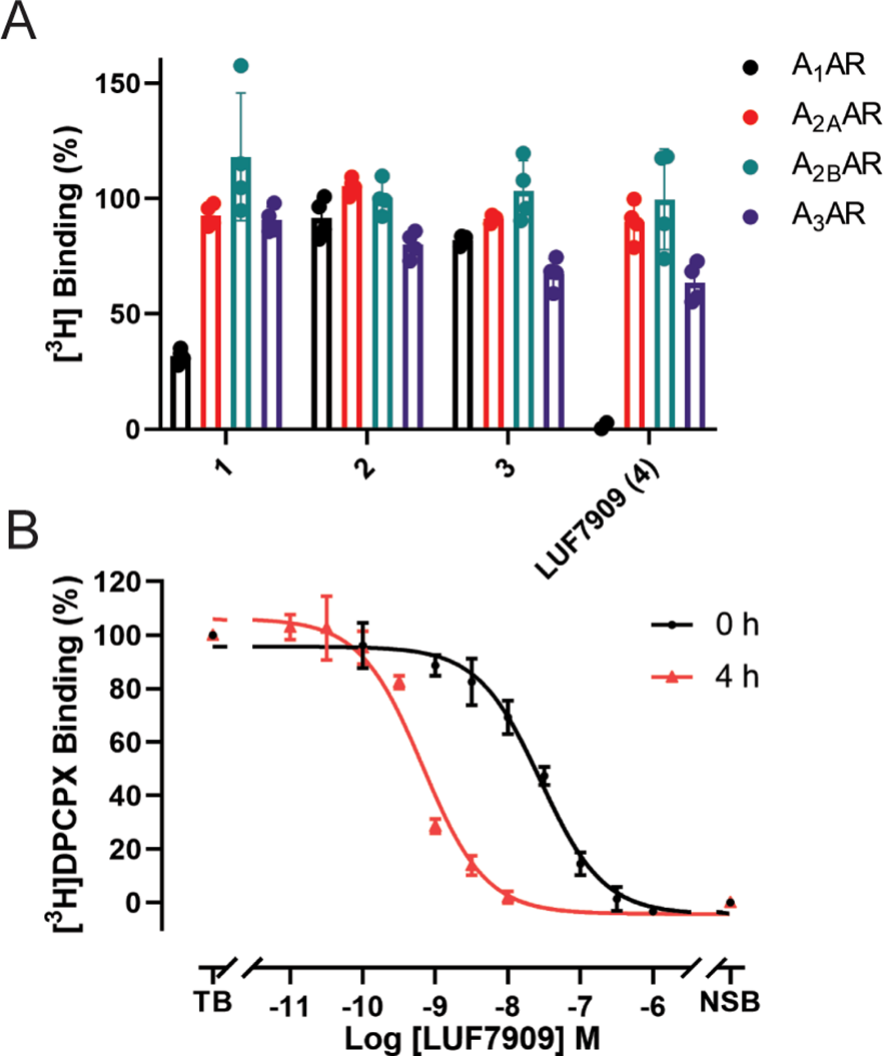
Affinities of LUF7909 and analogues for the four adenosine receptor subtypes. (A) Displacement of [^3^H]DPCPX (A_1_AR), [^3^H]ZM241385 (A_2A_AR), [^3^H]PSB-603 (A_2B_AR), and [^3^H]PSB-11 (A_3_AR) binding by 1 μM of the respective AfBP. Data represent the values of two individual experiments performed in duplicate and are normalized to the vehicle control (100%). (B) Displacement of [^3^H]DPCPX from the A_1_AR by LUF7909 measured after 0 or 4 h of preincubation of LUF7909 with CHO membranes stably overexpressing the A_1_AR. TB = total radioligand binding (vehicle control); NSB = nonspecific radioligand binding. Data represent the mean ± SEM of three individual experiments performed in duplicate.

With the molecules in hand, our attention was shifted toward the assessment of the potential probes’ binding affinity toward the A_1_AR, as well as the selectivity toward other adenosine receptors. The dicyanopyridine scaffold in particular is known to bind other adenosine receptor subtypes dependent on its substitutions.^37,38^ In a single-point radioligand displacement assay using 1 μM probe at all four adenosine receptors, no considerable displacement of the radioligand from the A_2A_AR, A_2B_AR, and A_3_AR was observed (Figure 2A). Probes **2** and **3** did not show considerable displacement of the radioligand from the A_1_AR, while **1** showed moderate (∼70%) displacement and LUF7909 full (∼100%) displacement. This indicates high affinity of LUF7909 toward the A_1_AR and selectivity over the other adenosine receptors.

**Figure 3:**
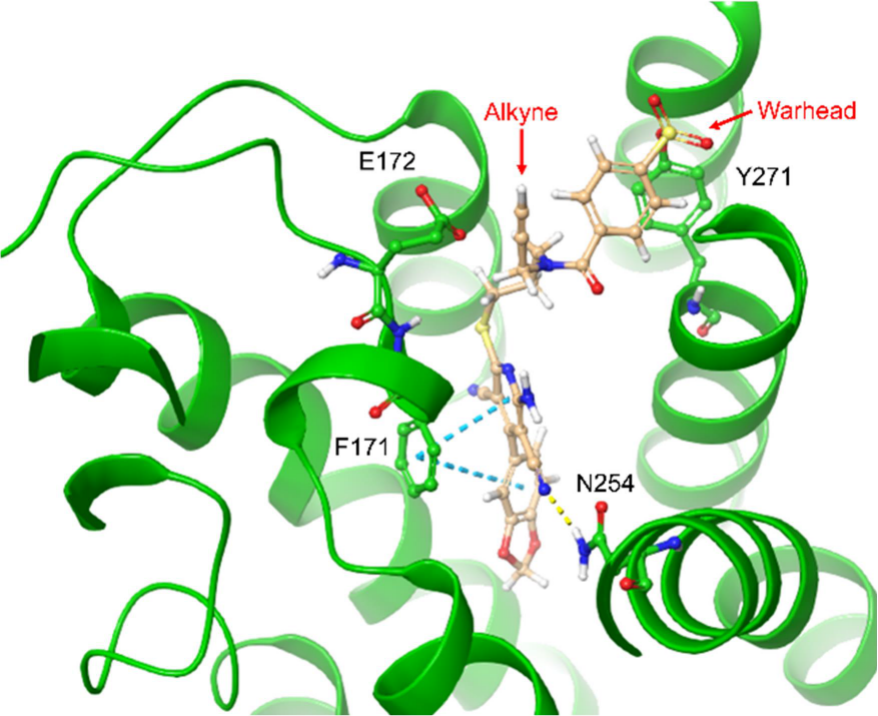
Top-down view of docked LUF7909 into the ligand binding pocket of the A_1_AR. Crystal structure taken from the adenosine-bound A_1_AR (PDB: 6D9H). The final ligand pose was selected based on the crystal structure of LUF5833 in the A_2A_AR (PDB: 7ARO). Shown are the main amino acids that interact with LUF7909. The SO_2_F-containing warhead is located close to tyrosine 271 (Y271), while the alkyne moiety is pointing outward from the receptor.

These differences can be rationalized by covalently docking the four ligands into the A_1_AR binding pocket. Using the crystal structure of adenosine-bound A_1_AR (PDB: 6D9H) and the binding pose of LUF7909 most similar (lowest RMSD) to the recently obtained crystal structure of structurally similar LUF5833 in the A_2A_AR, a representative image was generated (Figure 3).^39^ From this pose, it was deduced that the methylenedioxy group of LUF7909 is located deep inside the binding pocket of the A_1_AR. Substitutions at this position might therefore result in a loss in binding affinity, as observed for compounds **2** and **3**. Furthermore, the alkyne group of LUF7909 does not seem to hinder the important interactions that take place in the binding pocket (with residues F171, E172, and N254), thus explaining the high affinity.

Next, LUF7909 was submitted to a full curve radioligand displacement assay. To study the potential covalent binding mode of the probe, the assay was executed at two different time points, that is, incorporating 0 or 4 h of preincubation of probe with the receptor (Figure 2B). Without preincubation (0 h), LUF7909 showed an apparent p*K*_i_ of 7.8, while this increased to an apparent p*K*_i_ of 9.5 upon 4 h of preincubation (Table S1). Such an increase in apparent p*K*_i_ (*K*_i_ shift of 44.0) is a strong indicator of a covalent mode of action. A wash-out experiment confirmed the persistent mode of binding of LUF7909 to the receptor, even after multiple washing steps (Figure S1). Besides that, LUF7909 acted as a partial agonist in a functional [^35^S]GTPγS assay (Figure S2), having a similar potency to the full agonist *N*^6^-cyclopentyladenosine (CPA) (pEC_50_ of 8.7), but a significantly lower *E*_max_ (74%). Hence, LUF7909 behaves as a partial agonist that binds covalently, with high affinity and selectivity toward the A_1_AR.

### Labeling of the A_1_AR in SDS-PAGE Experiments

**Figure 4:**
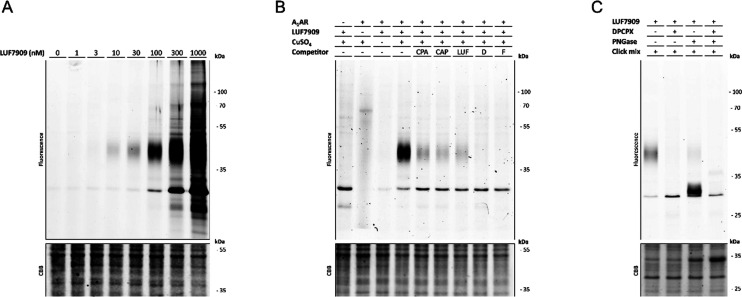
Specific labeling of the A_1_AR using LUF7909 in CHO membranes overexpressing the A_1_AR. Membranes were preincubated with or without competitor, incubated with LUF7909, subsequently “clicked” to AF647-N_3_, denatured, subjected to SDS-PAGE, and analyzed using in-gel fluorescence scanning. (A) Concentration-dependent labeling of the A_1_AR. (B) Labeling of the A_1_AR is dependent on the presence of copper(I) and probe during the click reaction, as well as the presence of known A_1_AR ligands: agonist CPA, partial agonist CAP, covalent partial agonist LUF7746 (LUF), antagonist DPCPX (D), and covalent antagonist FSCPX (F; structures in Figure S3). (C) Labeling of the A_1_AR shows a strong reduction in molecular weight upon incubation with PNGase. Preincubation with 1 μM DPCPX shows full disappearance of both bands. CBB = Coomassie Brilliant Blue. The band that appears upon Coomassie staining (lanes 3 and 4) corresponds to the molecular weight of PNGase.

As a first assessment of the labeling potential of our probe, SDS-PAGE experiments were carried out on CHO membranes that overexpress the A_1_AR. Membranes were incubated with LUF7909 and subjected to click conjugation of the probe to the fluorophore AF647-N_3_. Samples were denatured and loaded on gel, and the gel was visualized by in-gel fluorescence scanning. First, various concentrations of LUF7909 were investigated (Figure 4A). At roughly the apparent *K*_i_ of LUF7909 (16 nM at 0 h preincubation), a band at ∼45 kDa appeared on the gel. Increasing the concentration of LUF7909 revealed additional labeling of a protein at ∼30 kDa, where a probe concentration of 1 μM shows multiple extra proteins being labeled. This apparent nonspecific binding at high concentrations is presumably due to the electrophilic nature of the fluorosulfonyl warhead.

The optimal balance between selective labeling and intensity of the observed bands seemed to occur at a concentration of 100 nM of LUF7909 (Table S2), which was therefore used in further SDS-PAGE experiments. Of note, no labeling was observed in the absence of copper(I) or probe during the click reaction (Figure 4B). Furthermore, the band at ∼45 kDa disappeared upon preincubation with 1 μM of various A_1_AR-selective ligands, such as the full agonist CPA, partial agonist Capadenoson (CAP), parent compound LUF7746 (LUF), reference antagonist DPCPX (D), and covalent antagonist FSCPX (F; structures in Figure S3). Western blot experiments (Figure S4) further confirmed that the band at ∼45 kDa, though slightly higher than the expected mass of the A_1_AR (∼36 kDa), is indeed the A_1_AR.

The slightly higher mass can be explained by N-glycosylation of the A_1_AR, as has been seen in early purification studies of endogenous A_1_AR.^40,41^ Indeed, upon incubation with PNGase, a strong reduction is seen in the molecular weight of the corresponding band (Figure 4C). Preincubation with the reversible antagonist DPCPX resulted in full disappearance of both bands, thereby confirming that this pattern represents the A_1_AR.

### Enrichment and Detection of the A_1_AR in Chemical Proteomics Experiments

**Figure 5:**
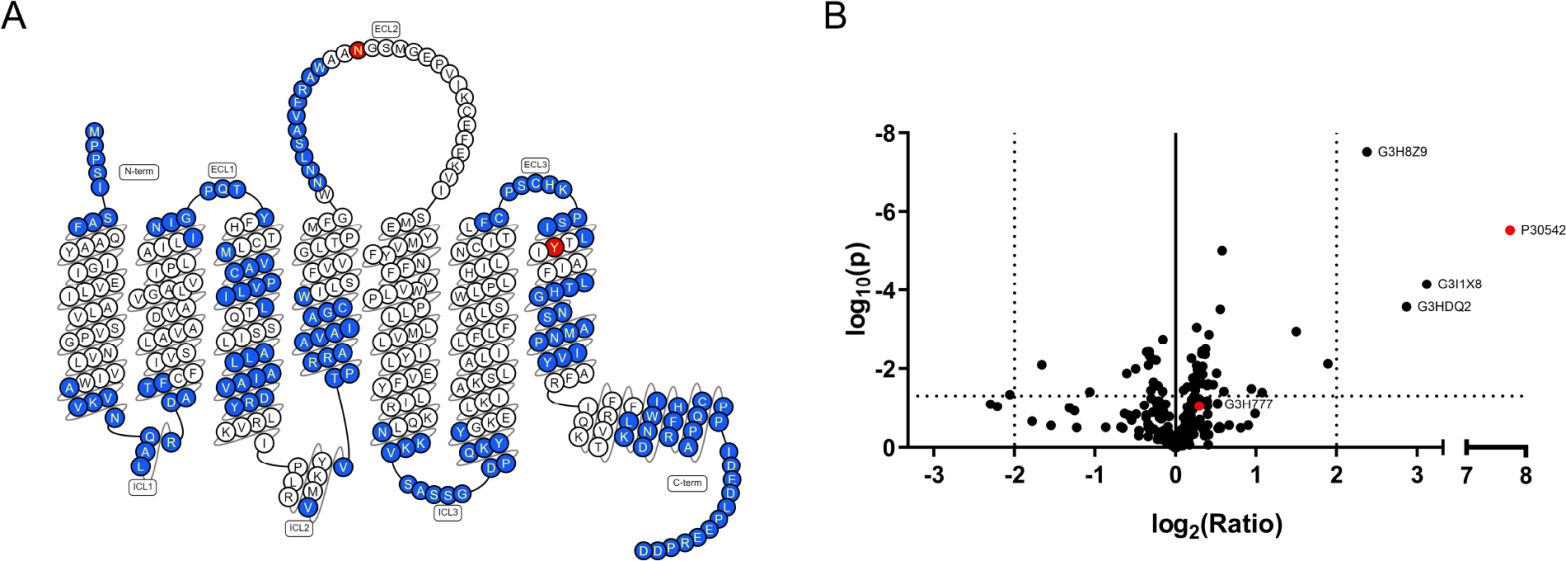
Proteomic detection of the A_1_AR. (A) Snake plot of the adenosine A_1_ receptor. Highlighted in blue are the peptides that were detected upon affinity purification using 1 μM LUF7909 in CHOhA_1_AR membranes. Amino acids highlighted in red are the glycosylation site (N159) and predicted probe binding site (Y271). Snake plot derived from gpcrdb.org.^49^ (B) Volcano plot of affinity purification experiments comparing samples treated with 1 μM LUF7909 to samples containing DMSO as the control. Plotted are the enrichment ratio (log_2_(Ratio)) and the probability (log_10_(*p*)) as determined in a multiple *t* test. All data originate from six technical replicates. The Uniprot codes are given for proteins that meet a threshold value of ratio > 2 and *p*-value < 0.05 (dotted lines). These are the G protein subunit beta-1 (G3I1X8), malate dehydrogenase (G3HDQ2), sodium/potassium-transporting ATPase subunit alpha (G3H8Z9), and the adenosine A_1_ receptor (P30542) (highlighted in red). Also shown in red is the adenine nucleotide translocator (G3H777).

To further explore binding of LUF7909 to the A_1_AR, as well as possible off-targets, chemical proteomics experiments were carried out. In brief, CHO cell membranes overexpressing the A_1_AR were incubated with LUF7909. A concentration of 1 μM was chosen to obtain a full proteomic profile of all the LUF7909-labeled proteins. Probe-bound proteins were “clicked” to biotin-azide, denatured, precipitated, reduced, alkylated, and pulled-down using streptavidin beads. The bound proteins were first washed and then digested. The obtained peptides were measured by LC–MS/MS. Initial attempts using trypsin as the digestion enzyme did not lead to detection of A_1_AR-specific peptides. Therefore chymotrypsin was chosen as the digestion enzyme. Experiments without LUF7909 lead to detection of only one peptide of the A_1_AR. However, upon affinity purification with LUF7909, an average sequence coverage of 40% of the receptor was detected by LC–MS/MS (Figure 5A; Table S2).

In the past years, considerable efforts have been made regarding the detection of GPCRs in chemical proteomics experiments.^28,42−46^ This includes mostly work using photoactivatable groups, such as diazirines and 2,5-disubstituted tetrazoles.^28,42,45,47^ To our knowledge, only one example of the use of an electrophilic ligand has been reported.^46^ Besides that, most of these experiments yielded a low sequence coverage when a nonpurified receptor was measured. Only in case of the metabotropic glutamate receptors in brain slices, a higher sequence coverage was found.^45^ It is therefore remarkable that our experiment yielded an average sequence coverage of 40%. The detected peptides mostly include the nonmembrane domains: C-terminus, N-terminus, intra-, and extracellular loops.

Compared to DMSO-treated samples, a strong enrichment of the A_1_AR (>200-fold) was observed (Figure 5B). This fold change was greatly reduced upon preincubation of the samples with 10 μM covalent ligand LUF7746 (Figure S5). Other proteins that showed a significant but substantially lower enrichment as compared to DMSO-treated samples were the G protein subunit beta-1, malate dehydrogenase, and ATPase subunit alpha. These off-targets might be the result of using a high concentration (1 μM) of electrophilic probe in membrane fractions.

### Investigation of the Potential Off-Targets of LUF7909 in CHOhA_1_AR Membrane Fractions

In Figure 4, it is visible that a potential off-target of LUF7909 (concentrations up to 300 nM) has an approximate molecular mass of 30 kDa. This molecular weight does not correspond to any of the proteins that were significantly “pulled-down” by LUF7909 during the chemical proteomics experiments. To further investigate this probable off-target, we performed similar chemical proteomics experiments, this time using CHO membrane fractions that do not overexpress the A_1_AR. Contrary to the CHOhA_1_AR membranes, only small-fold changes were observed for the detected proteins (vs DMSO control) (Figure S6A). There are however two proteins that show a significant enrichment (>4): Elongation factor 1-alpha 1 and an isoform of the adenine nucleotide translocator (ANT), the latter being an interesting target because of its binding of adenine-containing substrates. We therefore preincubated both CHO and CHOhA_1_AR membranes with various concentrations of bongkrekic acid, a known inhibitor of ANT,^48^ prior to the sequential addition of LUF7909, clicking to AF647-N_3_ subjecting to SDS-PAGE, and scanning using in-gel fluorescence (Figure S6B).

Preincubation with bongkrekic acid resulted in a concentration-dependent inhibition of the band observed for the “empty” CHO membranes, as well as the lower band observed for the CHOhA_1_AR membranes. This suggests that the extra band seen on gel corresponds to ANT. Also the pull-down experiments with LUF7909 in CHOhA_1_AR membranes show the presence of ANT (Figure 5B), although not significantly enriched compared to the DMSO control samples (fold change of 1.2). The reason for this may be the high expression level of ANT in mitochondria,^50^ causing an enrichment of ANT in our membrane fractions. We assume that binding of LUF7909 to ANT occurs because of a high concentration of ANT in combination with the electrophilic nature of the probe.

### Labeling of the A_1_AR on Live Cells

**Figure 6:**
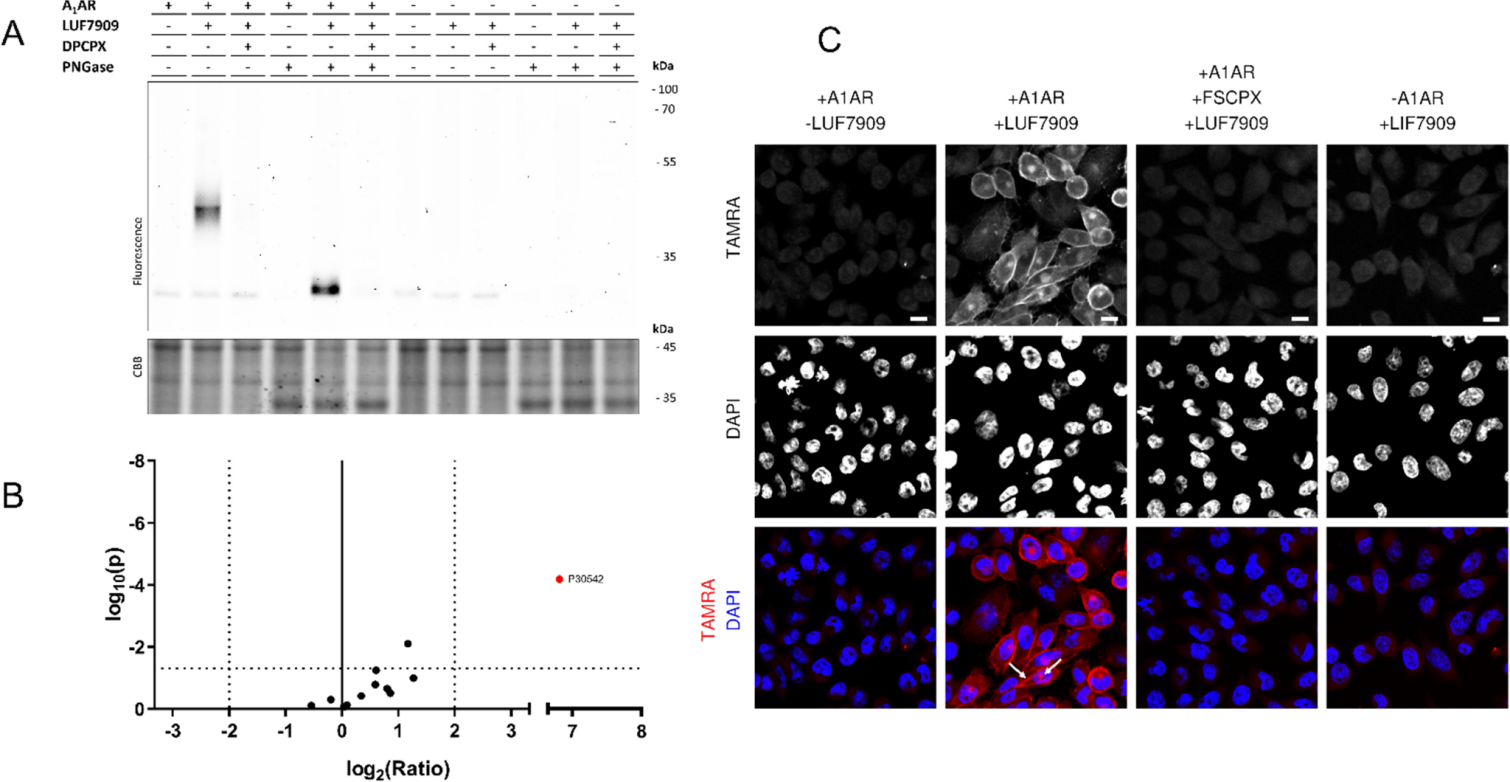
Selective labeling of the A_1_AR in live CHO cells. (A) CHO cells with or without overexpression of the A_1_AR were pretreated for 1 h with DPCPX (1 μM) or 1% DMSO and incubated for 1 h with LUF7909 (100 nM) or 1% DMSO (control). Membranes were collected, treated with PNGase, and incubated with click mix containing AF647-N_3_. The samples were then subjected to SDS-PAGE and analyzed by in-gel fluorescence scanning. CBB = Coomassie Brilliant Blue. (B) Volcano plot of affinity purification experiments comparing live CHOhA_1_AR cells treated with 1 μM LUF7909 to cells treated with 1% DMSO (control). All data originate from six technical replicates. Shown is the Uniprot code for the A_1_AR (P30542) (highlighted in red). (C) Confocal microscopy images. CHO cells with or without overexpression of the A_1_AR were pretreated for 1 h with FSCPX (1 μM) or 1% DMSO and incubated for 1 h with LUF7909 (100 nM) or 1% DMSO (control). The cells were then fixed and stained with TAMRA-N_3_ (first row) and DAPI (second row). The third row shows an overlay of both stains. TAMRA = red, DAPI = blue. Arrows indicate examples of labeled membranes and labeling inside cells. Images were selected manually as representatives of blinded measurements from two separate experiments (see Figure S8). Scale bar = 10 μm. Figure was created using OMERO.^52^

Moving a step closer to a more endogenous system, we tested the labeling properties of LUF7909 in live CHO cells. First, CHO cells with or without overexpression of the A_1_AR were incubated with 100 nM probe, prior to membrane collection and click reaction with AF647-N_3_. The samples were denatured, loaded on SDS-PAGE, and analyzed using in-gel fluorescence (Figures 6A and S7). A “smear” was observed at the height of the A_1_AR, which was not present upon preincubation with DPCPX (1 μM). This smear is presumably due to different glycosylation states of the A_1_AR. No other strong bands were detected, both in the CHO cells with and without overexpression of the A_1_AR. Second, affinity-based pull-down experiments were performed using live CHOhA_1_AR cells. Again, a high enrichment of the A_1_AR was found (Figure 6B), however less labeling of other proteins compared to the experiments with membrane fractions. It thus seems that labeling of the A_1_AR by LUF7909 is more specific in live CHOhA_1_AR cells, as compared to labeling in membrane fractions derived from these CHOhA_1_AR cells (Figures 4 and 5).

To confirm these observations, labeling of the receptor in live CHO cells was studied using confocal microscopy. Live CHO cells with or without overexpression of the A_1_AR were incubated with LUF7909 and subsequently fixed. The probe-bound proteins were stained with TAMRA-N_3_ (via a click reaction), and the cellular nuclei were stained with DAPI. As compared to the DMSO control, LUF7909 showed clear labeling of cell–cell contacts and cellular membranes by TAMRA-N_3_ (Figure 6C). The degree of labeling was diminished by preincubation with 1 μM covalent ligand FSCPX and absent upon using CHO cells that do not overexpress the A_1_AR. These controls confirm that the observed labeling, in gel, LC–MS/MS, and under the microscope, is indeed due to selective labeling of the A_1_AR and not an off-target protein. Furthermore, signs of A_1_AR labeling inside the cells were observed in Figure 6C. The reason for this might be internalization, an effect that has been reported to take place upon incubating the A_1_AR with an agonist.^51^ Having a clickable (partial) agonist thus allows further studies toward receptor internalization.

### Labeling of LUF7909 in Cells Endogenously Expressing the A_1_AR

**Figure 7:**
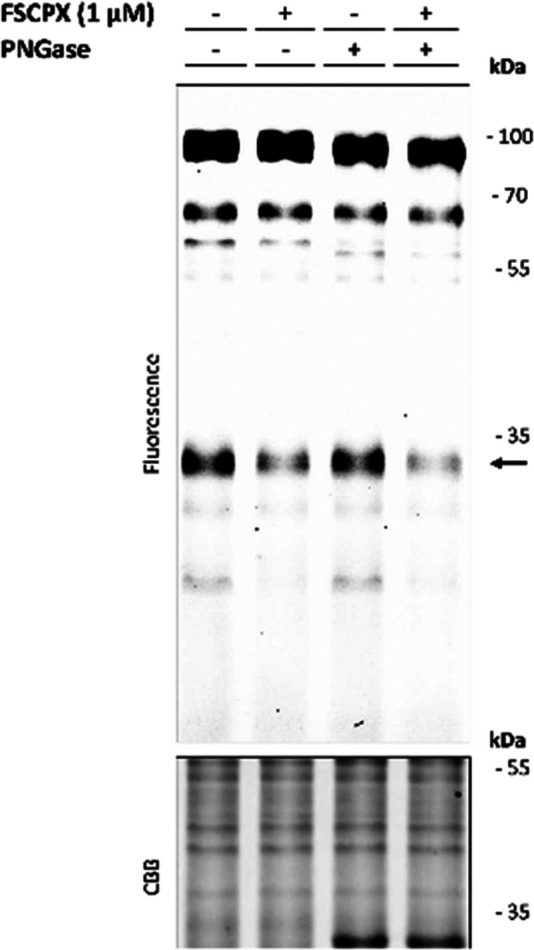
Labeling of the A_1_AR in adipocyte membranes derived from mouse gonadal fat pads. The membranes were pretreated with the covalent antagonist FSCPX (1 μM) or 1% DMSO prior to incubation with LUF7909 (100 nM) and subsequent incubation with click mix containing AF647-N_3_. The samples were then denatured, subjected to SDS-PAGE, and analyzed using in-gel fluorescence scanning. CBB = Coomassie Brilliant Blue. The band that appears upon Coomassie staining (lanes 3 and 4) corresponds to the molecular weight of PNGase.

Having established labeling of the A_1_AR in CHOhA_1_AR membranes and live cells, we attempted to label the receptor in membrane fractions that endogenously contain the A_1_AR as a next step. For this, we used membranes derived from adipocytes: a cell type that is known to express the A_1_AR.^7,53^ Adipocyte membranes were collected from gonadal fat pads from female mice and subsequently incubated with 100 nM LUF7909, deglycosylated with PNGase, clicked to AF647-N_3_, denatured, resolved by SDS-PAGE, and analyzed using in-gel fluorescence scanning. This resulted in multiple bands (lane 1, Figure 7) at molecular weights of roughly 90, 65, and 60 kDa and a smear of presumably two bands at 30 kDa. The intensity of the bands at 30 kDa (indicated with an arrow) is strongly diminished upon preincubation with 1 μM of the selective covalent A_1_AR antagonist FSCPX. Full reduction of this band was observed when using a high concentration of FSCPX (Figure S9A), which gives a strong indication that this band is in fact the A_1_AR.

The band at ∼30 kDa (lane 1) shows a great difference in mass compared to observed A_1_AR in CHO cells. The presumable reason for this is a difference in glycosylation pattern of the receptors, an effect that has also been observed when comparing the A_1_AR from brain to the A_1_AR from testis.^40^

In addition to the A_1_AR, LUF7909 also labeled multiple off-target proteins in these experiments, for example, the bands at ∼90 and ∼65 kDa. Preliminary experiments showed a clear reduction in intensity for the band at ∼90 kDa upon preincubation with a protease inhibitor cocktail (Figure S9B). This would indicate off-target binding to a presumable protease. We did not further examine this finding.

The labeling and visualization of low-abundant GPCRs in SDS-PAGE experiments thus seem to be challenging when high levels of potential off-targets are present. For future studies using AfBPs, we therefore suggest to perform affinity-based pull-down proteomics, confocal microscopy, or flow cytometry experiments to investigate GPCRs on native cells and tissues.

## Conclusions

In this study, we have described the design and synthesis of LUF7909, a versatile probe molecule acting as a partial agonist that was used to characterize the A_1_AR in a broad spectrum of assays. LUF7909 showed labeling of the A_1_AR at about its apparent *K*_i_, as well as labeling of other proteins in SDS-PAGE experiments. The observed off-targets on gel were not significantly enriched in proteomic studies, nor found in live cell experiments, both carried out in A_1_AR overexpressing cells. In the latter two types of experiments, LUF7909 proved to be highly specific toward the A_1_AR.

Altogether, this work shows various methods to implement AfBPs within the broad field of GPCR research. This paves the way toward an investigation of more physiologically relevant processes, for example, the presence/absence of PTMs on GPCRs (through LC–MS/MS investigations) and receptor internalization (through confocal microscopy). This will ultimately help to get a better understanding of GPCRs under both physiological and pathological conditions, opening up new avenues for drug discovery in general.

## Methods

See the Supporting Information.

## Supporting Information Available

Additional tables and figures, experimental procedures, synthetic procedures, and NMR spectra (PDF)
Labeling the adenosine A1 receptor using an electrophilic covalent probe (xlsx)
