# Supplementary material for: A Chemical Biological Approach to Study G Protein-Coupled Receptors: Labeling the Adenosine A1 Receptor Using an Electrophilic Covalent Probe
Source: ACS Chem Biol. 2022 Oct 24;17(11):3131–9. doi: 10.1021/acschembio.2c00589 (PMC9679998; doi:10.1021/acschembio.2c00589)
Supplement: Supplementary file 1 — cb2c00589_si_001.pdf [file cb2c00589_si_001.pdf]

Supporting Information

A Chemical Biological Approach to Study G Protein-Coupled Receptors: Labeling the Adenosine A_1 Receptor using an Electrophilic Covalent Probe

Bert L. H. Beerkens,[1] Çağla Koç,[1] Rongfang Liu,[1] Bogdan I. Florea,[2] Sylvia E. Le Dévédec,[1] Laura H. Heitman,[1,3] Adriaan P. IJzerman,[1] and Daan van der Es*[1]

[1] Division of Drug Discovery and Safety, Leiden Academic Centre for Drug Research, Leiden University, Einsteinweg 55, 2333 CC Leiden, The Netherlands
[2] Department of Bioorganic Synthesis, Leiden Institute of Chemistry, Leiden University, Einsteinweg 55, 2333 CC Leiden, The Netherlands
[3] Oncode Institute, 2333 CC Leiden, The Netherlands
* Corresponding author, email: d.van.der.es@lacdr.leidenuniv.nl

Table of contents

Supporting Tables 3
Supporting Figures 6
Experimental procedures 16
General Chemistry 16
Synthetic Procedures 16
Computational Modelling 27
General Biology 28
Cell lines 28
Radioligands 28
Chemicals 28
Biologicals 28
Fat pads 28
Cell culture and membrane preparation 29
Preparation of adipocyte membranes from mouse gonadal fat pads[4–6] 29
Radioligand displacement assays 30
SDS-PAGE experiments of LUF7909 in membrane fractions 31
SDS-PAGE experiments of LUF7909 in live CHOhA_1AR and CHO cells 31
Affinity-based proteomics[11] 32
Nano-LC-MS Settings 33
LC-MS/MS Data processing 34
Click microscopy experiments using LUF7909 in CHOhA_1AR and CHO cells[15] 34
Image acquisition 35
NMR Spectra 36
References 61

Supporting Tables

Table S1. Time-dependent characterization of the affinity of **LUF7909.**

Compound	pKi (0 h)[a]	pKi (4 h)[b]	Ki shift[c]
**LUF7909**	7.8 ± 0.04	9.5 ± 0.01	44.0 ± 5.1

Values represent apparent pKi ± SEM (n = 3) of individual experiments each performed in duplicate. [a] Affinity determined from displacement of specific [^{3}H]DPCPX binding on CHO cell membranes stably expressing hA_1AR at 25 °C after 0.5 h co-incubation; [b] Affinity determined from displacement of specific [^{3}H]DPCPX binding on CHO cell membranes stably expressing hA_1AR at 25 °C with compounds pre-incubated for 4 h, followed up by a 0.5 h co-incubation with [^{3}H]DPCPX. [c] Ki shift determined by ratio Ki(0 h)/Ki(4 h).

Table S2. Concentration-dependent labeling by **LUF7909** in SDS-PAGE experiments. A concentration of 100 nM **LUF7909** shows both a high intensity (70% intensity compared to the band at 1000 nM) and a low degree of off-target labeling (11%).

Concentration LUF7909	Relative band intensity (%)[a]	A_1AR labeling vs. off-target labeling[b]
**1**	1 ± 0	31 ± 10
**3**	5 ± 1	84 ± 6
**10**	16 + 6	86 ± 5
**30**	44 ± 12	95 ± 1
**100**	70 ± 9	89 ± 4
**300**	90 ± 14	74 ± 8
**1000**	100	51 ± 13

Values represent the mean percentage ± SEM (n = 3). Band intensities were determined with ImageLab software, using the gel images from Figure S3A. [a] The adjusted volumes of the bands were taken and corrected for the amount of protein after Coomassie staining. The highest band intensity (1000 nM probe) was set to 100%; [b] The bands at approx. 45 kDa (A_1AR) and approx. 30 kDa were selected in each lane and band percentages were calculated by ImageLab. The value in the table shows the percentage of the upper band (band 45 kDa (%) + band 30 kDa (%) = 100 (%)).

Table S3. Detected A_1AR peptides upon affinity purification using LUF7909 in CHO membranes that overexpress the A_1AR.

Sequence	Length	m/z	Charges	Start position	End position	Peptide score[a]
MPPSISAF	8	848.4102	1	1	8	150.82
PPSISAF	7	717.3697	1	2	8	131.42
AVKVNQALRDATF	13	1431.783	3	33	45	58.781
INIGPQTY	8	904.4654	1	69	76	176.41
MVACPVLIL	9	1014.561	2	82	90	97.306
ALLAIAVDRY	10	1103.634	2	97	106	78.334
LAIAVDRY	8	919.5127	2	99	106	104.45
AIAVDRY	7	806.4287	2	100	106	103.29
VVTPRRAAVAIAGCW	15	1625.882	2;3	118	132	58.676
NNLSAVERAW	10	1158.578	2	147	156	131.44
NKKVSASSGDPQKY	14	1507.763	2;3	212	225	197.3
NKKVSASSGDPQKYY	15	1670.826	2;3	212	226	156.51
FCPSCHKPSIL	11	1344.632	3	259	269	47.559
CPSCHKPSIL	10	1197.563	2;3	260	269	128.6
LTHGNSAMNPIVY	13	1415.687	2	276	288	156.83
THGNSAMNPIVY	12	1302.603	2	277	288	157.96
LKIWNDHF	8	1071.55	2;3	300	307	120.65
KIWNDHF	7	958.4661	2	301	307	158.38
RCQPAPPIDEDLPEERPDD	19	2248.007	2;3	308	326	218.16

[a]As determined by MaxQuant.

Supporting Figures

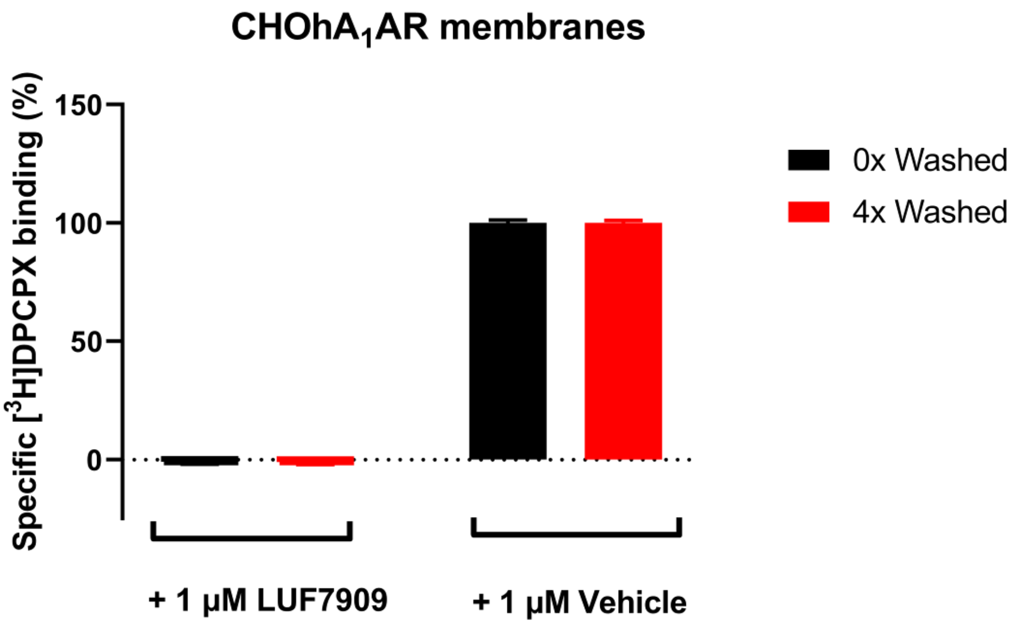

Figure S1 Wash-out assay reveals persistent binding of LUF7909 to the A_1AR. Membranes derived from CHO cells transiently transfected with the A_1AR were pre-incubated with buffer (vehicle) or 1 µM LUF7909, followed by a four cycle washing treatment or no washing at all before being exposed to $[^3H]$DPCPX in a standard radioligand binding assay. Data is expressed as the percentage of the vehicle group (100%) and represents the mean ± SEM of three individual experiments performed in duplicate.

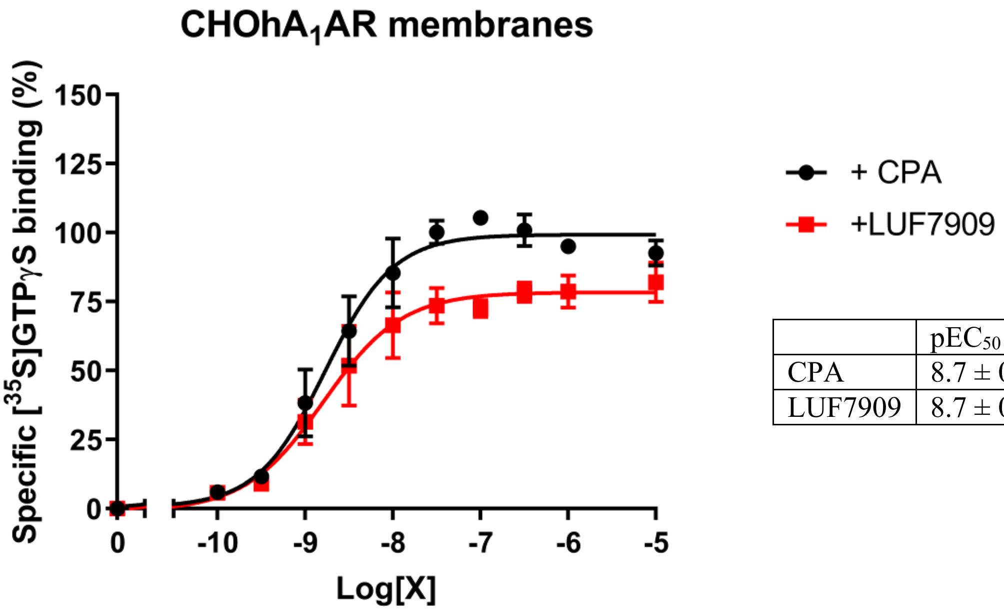

	pEC_{50}	E_{max}
CPA	8.7 ± 0.2	100 ± 3
LUF7909	8.7 ± 0.3	74 ± 3**

Figure S2 Functional characterization of LUF7909 in a [^{35}S]GTPγS binding assay. Concentration-dependent functional-effect curve of CPA and LUF7909 using membranes derived from CHO cells that were stably transfected with the hA_1AR. Data is expressed as a percentage of the maximal response induced by 100 nM CPA and the mean ± SEM of three individual experiments performed in duplicate. **$p < 0.01$ as compared to the E_{max} value of CPA, determined by a two-tailed unpaired Student's *t*-test.

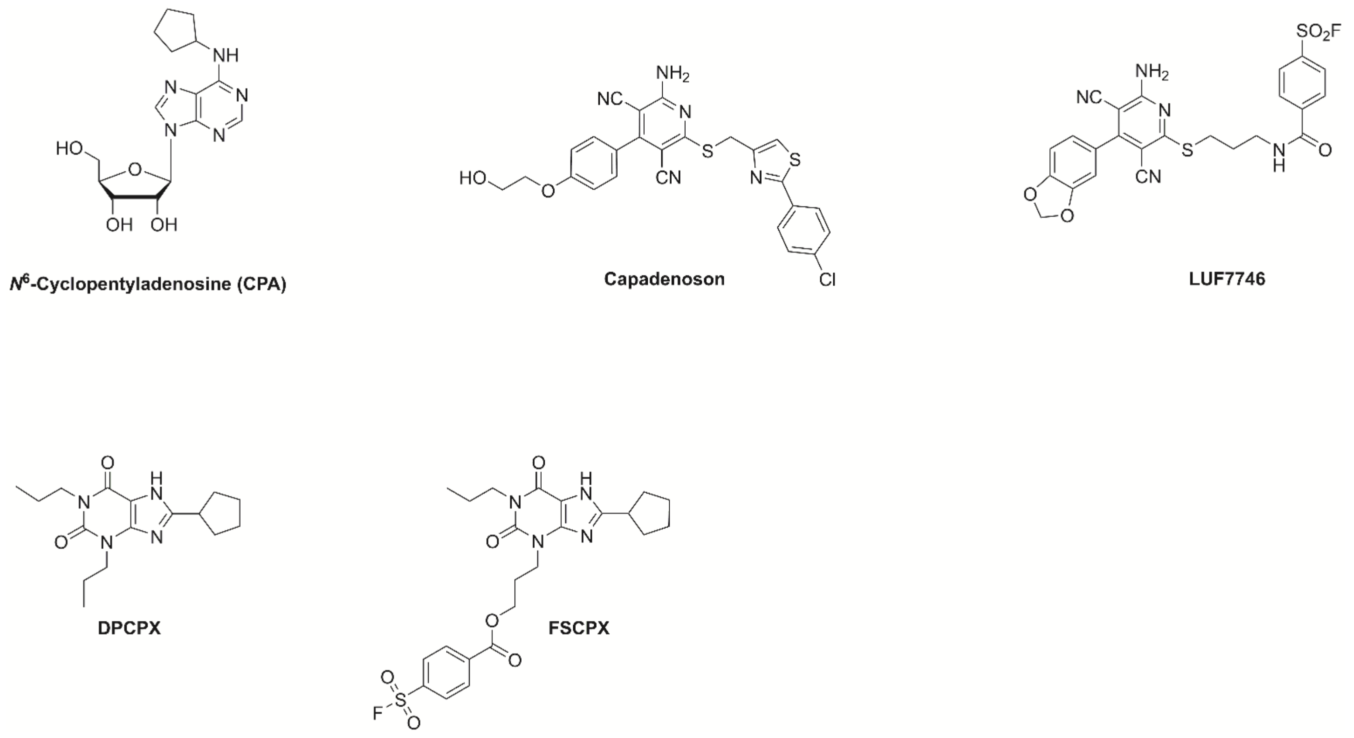

Figure S3. Molecular structures of the selective A_1AR ligands used in this study: full agonist N^6-cyclopentyladenosine (CPA), partial agonist Capadenoson, covalent partial agonist LUF7746, antagonist DPCPX and covalent antagonist FSCPX.

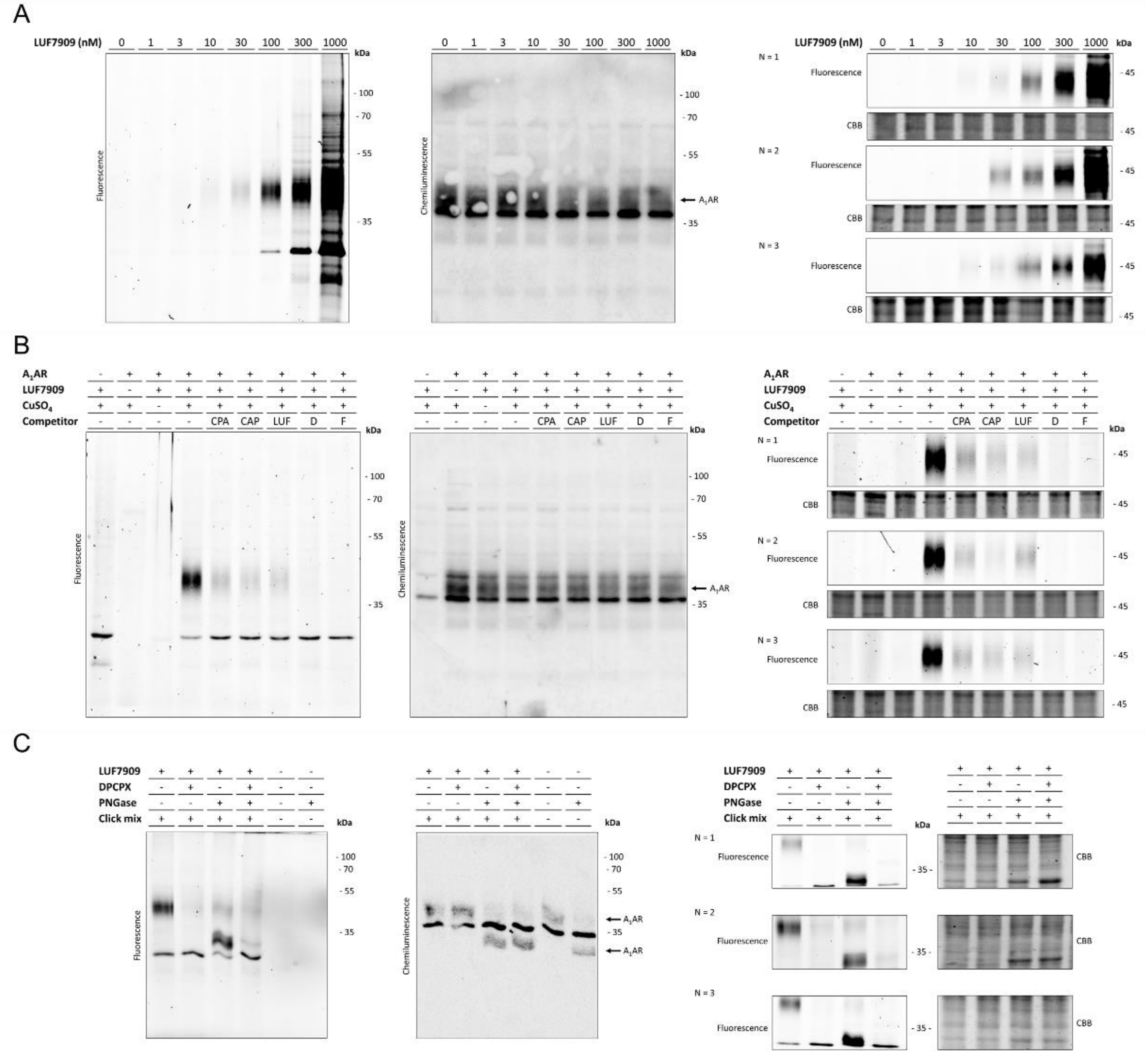

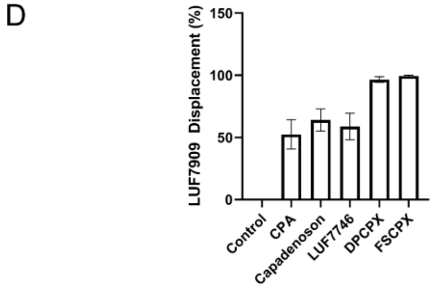

Competing ligand (1 μM)	LUF7909 Displacement (%)
None (control)	0 ± 0
CPA	53 ± 7
Capadenoson	64 + 5
LUF7746	59 ± 6
DPCPX	97 ± 1
FSCPX	100 ± 0

Figure S4. Verification of A_1AR labeling by **LUF7909**. Figures show SDS-PAGE and Western Blot experiments (left side), as well as N=3 data (right side). Conditions for A-D were the same as in Figure 4. The gel was transferred to a 0.2 μm PVDF blot using a Trans-Blot Turbo Transfer System (Bio-Rad)(2.5 A, 7 min) or stained with Coomassie Brilliant Blue (CBB). Blots were blocked with 5% BSA in TBST (1 h, rt), incubated with primary antibody (rabbitαratA_1AR 1:5000 in 1% BSA in TBST)(4 °C, overnight), washed (3 x TBST), incubated with secondary antibody (goatαrabbit-HRP 1:2000 in 1% BSA in TBST)(1h , rt), washed (2 x TBST, 1 x TBS), activated with luminol enhancer and peroxide (3 min, rt, dark) and subsequently scanned on fluorescence and chemiluminescence. (D) Quantification of the amount of **LUF7909** displaced by 1 μM of the respective covalent ligand. Values represent the mean percentage ± SEM (n = 3). Band intensities were determined with ImageLab software using the gel images from Figure S4B. The adjusted volumes of the bands were taken and corrected for the amount of protein after Coomassie staining. The band intensity of lane 4 (no competitor) was set to 0%.

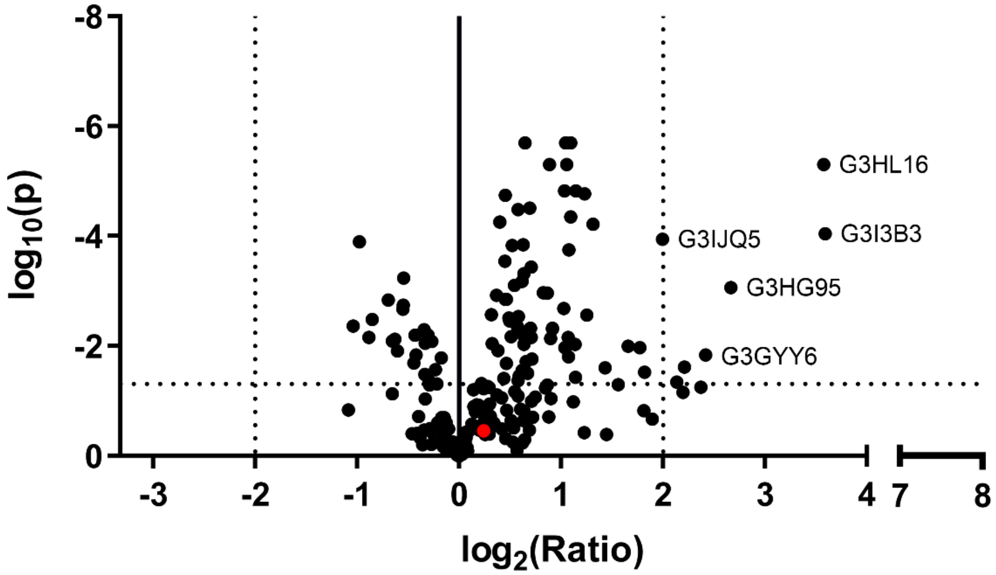

Figure S5. The adenosine A_1 receptor does not show a strong enrichment upon pre-incubation of the samples with **LUF7746**. Volcano plot of affinity purification experiments comparing samples treated with 1 μM **LUF7909** to samples that were pre-treated with 10 μM of **LUF7746**. Plotted are the enrichment ratio ($\log_2$(Ratio)) and the probability ($\log_{10}$(p)) as determined in a multiple *t* test. All data originate from six technical replicates. The Uniprot codes are given for proteins that meet a threshold value of ratio>2 and p-value<0.05 (dotted lines). These are the Splicing factor 3B subunit 1 (G3HL16), Nuclear pore complex protein Nup96 (G3I3B3), Lamin-A/C (G3HG95), Catalase (G3GYY6) and Protein RCC2 (G3IJB6). The adenosine A_1 receptor (P30542) is highlighted in red.

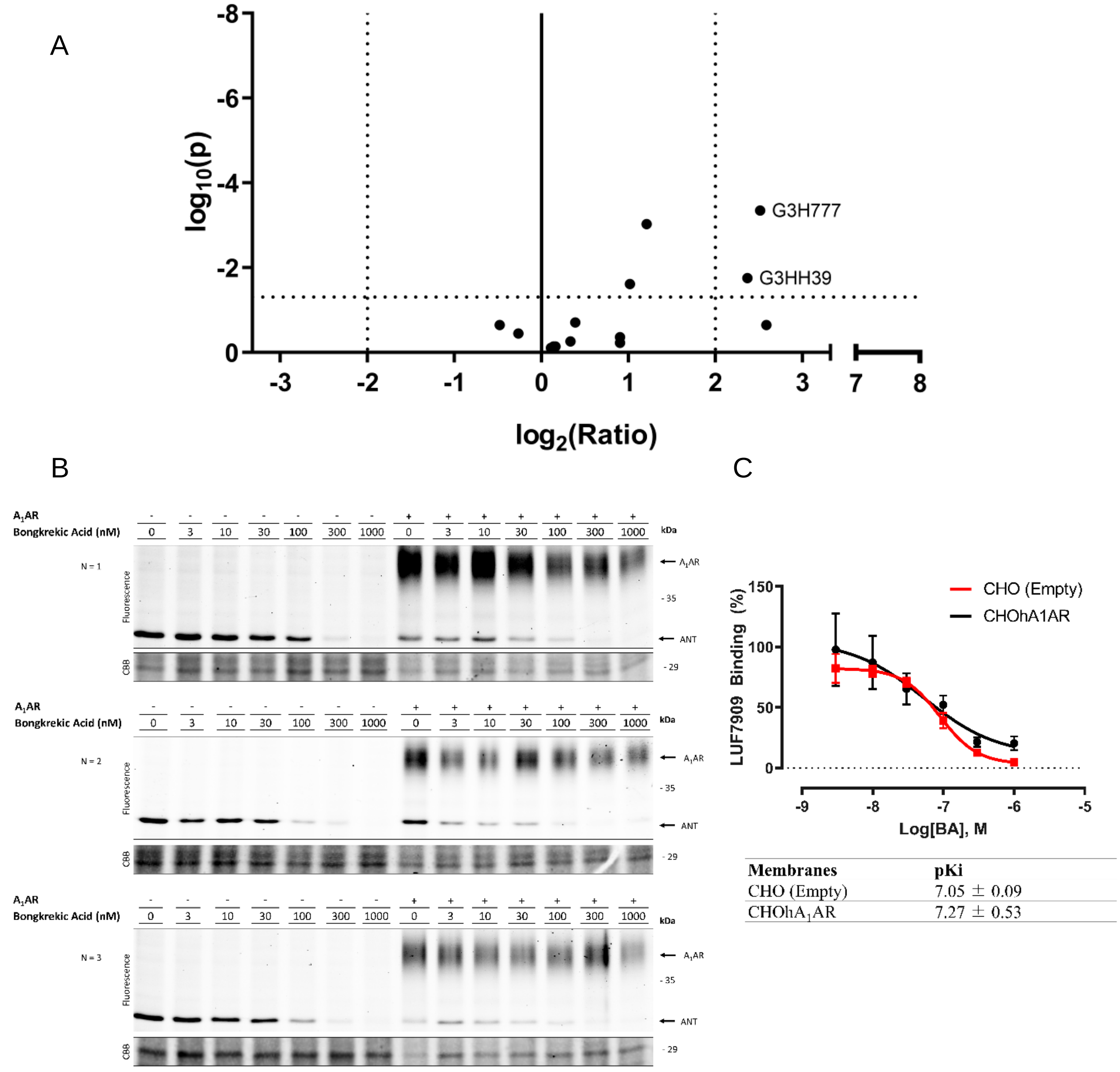

Membranes	pKi
CHO (Empty)	7.05 ± 0.09
CHOhA$_1$AR	7.27 ± 0.53

Figure S6. Off-target investigation of the proteins labeled by **LUF7909**. (A) Volcano plot of affinity purification experiments comparing samples of CHO membrane fractions (not overexpressing the A$_1$AR) treated with 1 µM **LUF7909** to samples treated with 1% DMSO. Plotted are the enrichment ratio (log$_2$(Ratio)) and the probability (log$_{10}$(p)) as determined in a multiple *t* test. All data originate from six technical replicates. The Uniprot codes are given for proteins that meet a threshold value of ratio>2 and p-value<0.05 (dotted lines). These are the Adenine Nucleotide Translocator (ANT)(G3H777) and Elongation factor 1-alpha 1 (G3HH39). (B) SDS-PAGE experiments show a concentration-dependent inhibition of the lower band by the ANT-inhibitor bongkrekic acid (**BA**) (N=3). CHO membrane fractions with and without overexpression of the A$_1$AR were pre-incubated with various concentrations of **BA**, prior to incubation with 100 nM of **LUF7909**. The samples were then clicked to AF647-N$_3$, denatured, resolved by SDS-PAGE and scanned using in-gel fluorescence. Coomassie Brilliant Blue (CBB) was used as protein loading control. (C) Quantification of **LUF7909** displacement by **BA**. Values represent the mean percentage ± SEM (graph) or apparent affinity ± SEM (table) (n = 3). Band intensities were determined with ImageLab software using the gel images from Figure S6B. The adjusted volumes of the bands were taken and corrected for the amount of protein after Coomassie staining. The band intensity of lane 1 (no **BA**) was set to 100%.

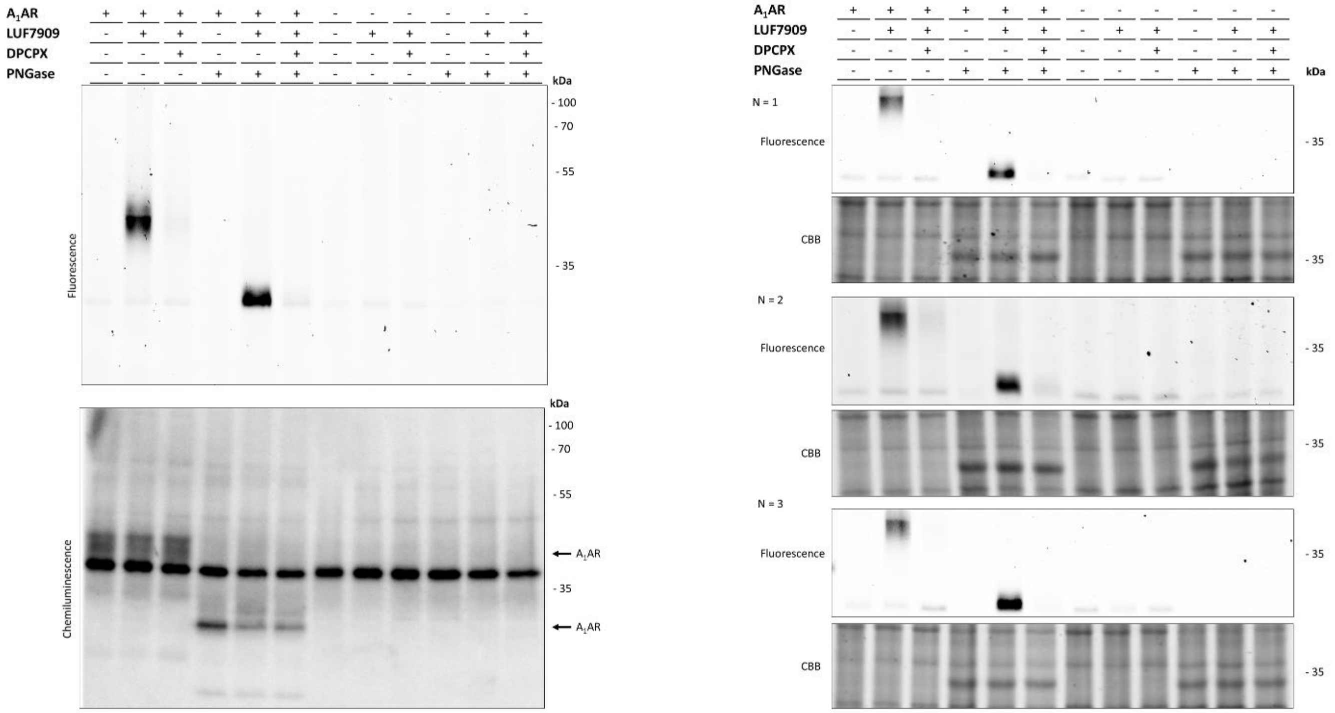

Figure S7 Additional SDS-PAGE experiments supporting Figure 6. Figure shows SDS-PAGE and Western Blot experiments (left side), as well as N=3 data (right side). Live CHOhA$_1$AR and non-transfected CHO cells were pre-treated with DPCPX (1 µM) or 1% DMSO and incubated with **LUF7909** (100 nM) or 1% DMSO (control). Membranes were collected and incubated with click mix containing AF647-N$_3$. The samples were then subjected to SDS-PAGE and analysed by in-gel fluorescence scanning. The gel was transferred to a 0.2 µm PVDF blot using a Trans-Blot Turbo Transfer System (Bio-Rad)(2.5 A, 7 min) or stained with Coomassie Brilliant Blue (CBB). The blot was blocked with 5% BSA in TBST (1 h, rt), incubated with primary antibody (rabbitαratA$_1$AR 1:5000 in 1% BSA in TBST)(4 °C, overnight), washed (3 x TBST), incubated with secondary antibody (goatαrabbit-HRP 1:2000 in 1% BSA in TBST)(1h , rt), washed (2 x TBST, 1 x TBS), activated with luminol enhancer and peroxide (3 min, rt, dark) and subsequently scanned on fluorescence and chemiluminescence.

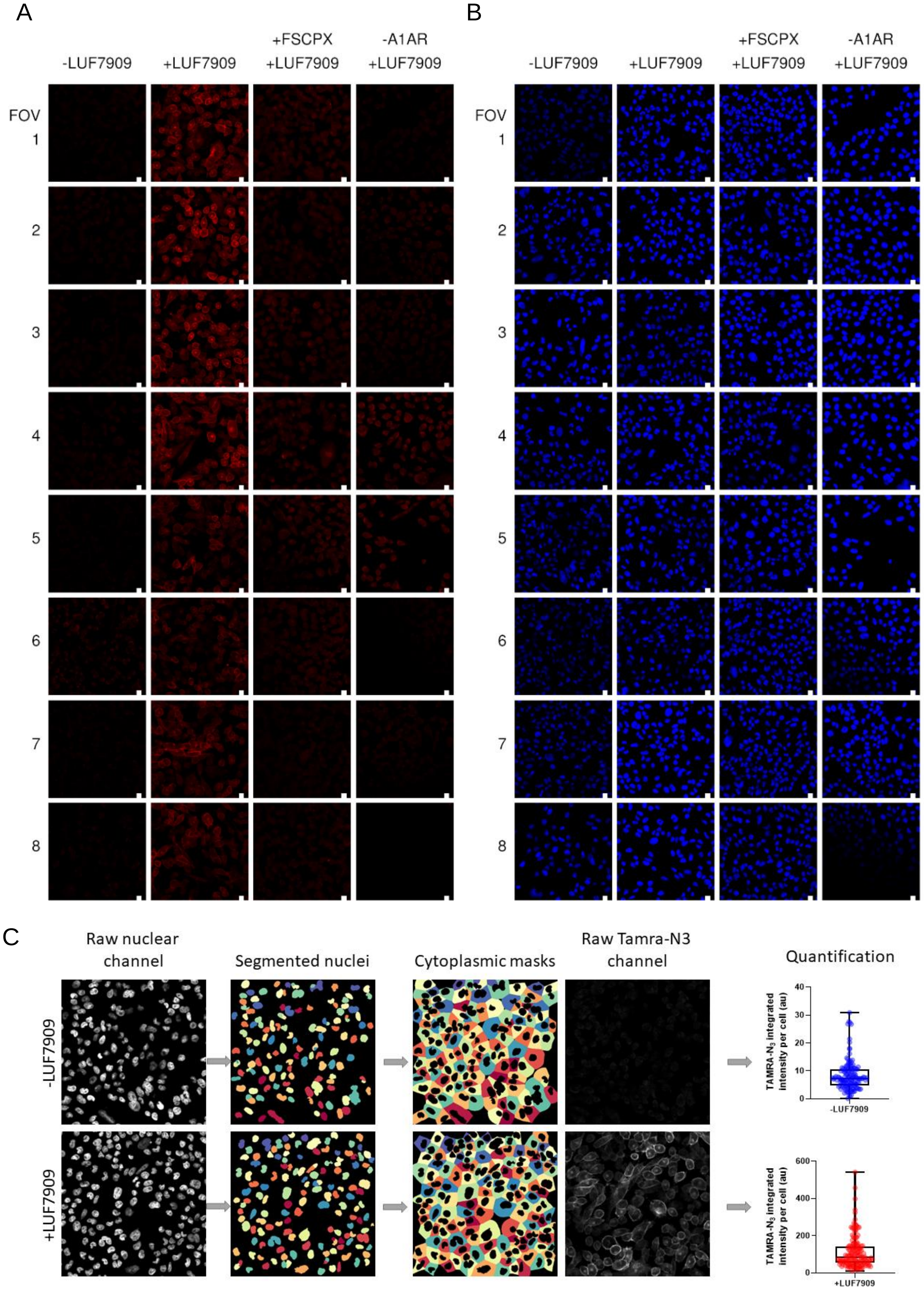
A
B
-LUF7909
+LUF7909
+FSCPX
+LUF7909
-A1AR
+LUF7909
FOV
1
2
3
4
5
6
7
8
C
Raw nuclear channel
Segmented nuclei
Cytoplasmic masks
Raw Tamra-N3 channel
Quantification
-LUF7909
+LUF7909
TAMRA-N3 integrated intensity per cell (au)
0
10
20
30
40
200
400
600

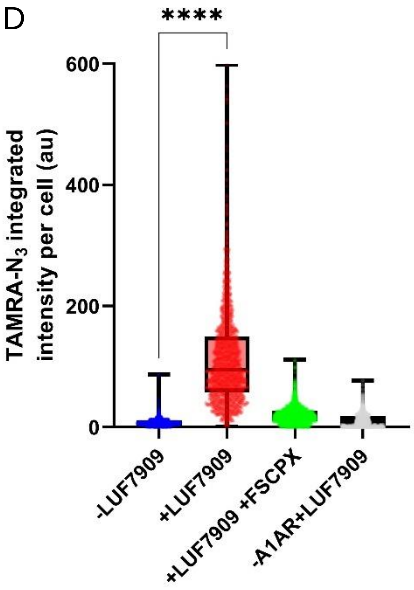

Figure S8. Additional confocal microscopy images supporting Figure 6C. CHO cells with or without overexpression of the A_1AR were pre-treated for 1 h with irreversible antagonist FSCPX (1 µM) or 1% DMSO and incubated for 1 h with LUF7909 (100 nM) or 1% DMSO (control). The cells were then fixed and stained with TAMRA-N_3 (panel A; shown in the red channel) and DAPI (panel B; shown in the blue channel). FOV = Field of view. Images were acquired automatically from two biological experiments performed in duplo shown are four of the FOVs from each of the imaged plates, selected based on a similar amount of cells present in the FOV (FOV 1-4: experiment 1; FOV 5-8: experiment 2). Scale bar = 10 µm. Panel was created using OMERO. (C) Example of automated image analysis of the total intensity of the Tamra-N_3 signal per single cell with and without treatment with LUF7909. Total fluorescence intensity in the cytoplasm of single cells was quantified using CellProfiler. (D) Comparison of the fluorescence intensities between treatment conditions in experiment 2. One single dot represent one single cell. A significant increase in intensity is observed for the cells containing the A_1AR, treated with LUF7909, without competing ligand versus those cells not treated with LUF7909.

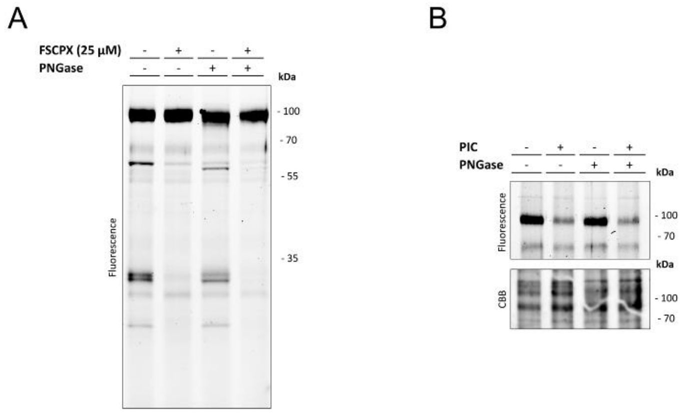

Figure S9 Labeling of LUF7909 in membranes derived from rat adipocytes. The membranes were pre-incubated with competing ligand (25 µM FSCPX or 1 µL protease inhibitor cocktail (PIC), prior to incubation with 100 nM LUF7909. The samples were then incubated with PNGase, clicked to AF647-N_3, denatured and resolved by SDS-PAGE. The gels were scanned using in-gel fluorescence. (A) Competition with 25 µM of covalent antagonist FSCPX; (B) Intensity of the band at 90 kDa is reduced upon pre-incubation with 1 µL of PIC (Sigma Aldrich, cat # p8340).

Experimental procedures

General Chemistry

All commercially available reagents and solvents were obtained from Sigma Aldrich, Fisher Scientific, VWR chemicals, and Biosolve. All reactions were carried out under a N_2 atmosphere in oven-dried glassware. Thin layer chromatography was performed on TLC Silica gel 60 F254 (Merck) and visualized using UV irradiation. Silica gel flash chromatography was performed using 60-200 µm 60 Å silica gel (VWR Chemicals). ^{1}H-NMR spectra were recorded on a Bruker AV-300 (300 MHz), Bruker AV-400 (400 MHz) or a Bruker AV-500 spectrometer (500 MHz). ^{13}C-NMR spectra were recorded on a Bruker AV-400 (101 MHz) or a Bruker AV-500 (126 MHz) spectrometer. ^{19}F-NMR spectra were recorded a Bruker AV-500 spectrometer (471 MHz). Chemical shift values are reported in ppm (δ) using tetramethylsilane or solvent resonance as the internal standard. Coupling constants (J) are reported in Hz. Multiplicities are indicated by s (singlet), d (doublet), t (triplet), q (quartet), p (pentet) or m (multiplet) followed by the number of represented hydrogen atoms. Compound purity was determined by LC-MS, using the LCMS-2020 system of Shimadzu coupled to a Gemini® 3 µm C18 110Å column (50 x 3 mm). In brief, compounds were dissolved in H_2O:MeCN:*t*-BuOH 1:1:1, injected onto the column and eluted with a linear gradient of H_2O:MeCN 90:10 + 0.1% formic acid → H_2O:MeCN 10:90 + 0.1% formic acid over the course of 15 minutes. High-resolution mass spectrometry (HRMS) was performed on a X500R QTOF mass spectrometer (SCIEX). Reaction schemes were created with ChemDraw Professional version 16.0.0.82 (PerkinElmer).

Synthetic Procedures

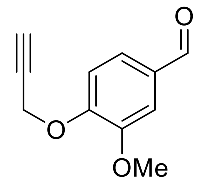

3-Methoxy-4-(prop-2-yn-1-yloxy)benzaldehyde (6a)
3-methoxy-4-hydroxy benzaldehyde (3.80 g, 25.0 mmol, 1.0 eq), propargyl bromide (80% in toluene) (8.30 ml, 74.5 mmol, 3.0 eq) and K_2CO_3 (5.18 g, 37.5 mmol, 1.5 eq) were dissolved acetone (250 mL). The mixture was refluxed overnight, after which a precipitate had formed. The precipitate was filtrated, dried under reduced pressure and purified by flash column chromatography (PE/EtOAc 95:5 → 70:30) to yield **6a** (3.80 g, 20.0 mmol, 80%) as a white solid.
TLC (PE/EtOAc 7:3): R_f = 0.57.
^{1}H NMR (400 MHz, $CDCl_3$): δ [ppm] = 9.87 (s, 1H), 7.46 (dd, *J* = 8.2, 1.9 Hz, 1H), 7.43 (d, *J* = 1.9 Hz, 1H), 7.14 (d, *J* = 8.2 Hz, 1H), 4.86 (d, *J* = 2.4 Hz, 2H), 3.94 (s, 3H), 2.56 (t, *J* = 2.4 Hz, 1H).

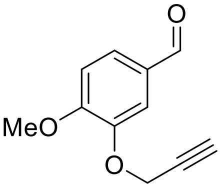

4-Methoxy-3-(prop-2-yn-1-yloxy)benzaldehyde (6b)
3-hydroxy-4-methoxybenzaldehyde (608 mg, 4.00 mmol, 1.0 eq) and K_2CO_3 (830 mg, 6.01 mmol) were dissolved in acetone (20 mL). Propargylbromide (80% in toluene) (1.33 mL, 12.0 mmol, 3.0 eq) was

added and the solution was refluxed for 1 h at 80 °C and allowed cooled down to rt overnight. Water (40 mL) was added and the mixture was extracted with EtOAc (3 x 40 mL). The organic layers were combined and washed with brine (1 x 50 mL), dried over $MgSO_4$ and evaporated to yield **6b** (759 mg, 3.99 mmol, 100 %) as an off-white oil.

TLC (PE/EtOAc 3:2): $R_f = 0.67$.

^{1}H NMR (400 MHz, $CDCl_3$): δ [ppm] = 9.66 (s, 1H), 7.42 – 7.27 (m, 2H), 6.83 (d, *J* = 8.1 Hz, 1H), 4.64 (d, *J* = 2.5 Hz, 2H), 3.76 (s, 3H), 2.50 (t, *J* = 2.4 Hz, 1H).

^{13}C NMR (101 MHz, $CDCl_3$): δ [ppm] = δ 190.3, 154.5, 146.9, 129.5, 126.9, 111.5, 110.7, 77.6, 76.4, 56.2, 55.8.

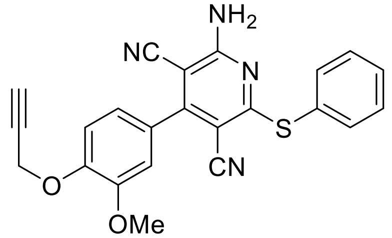

2-Amino-4-(3-methoxy-4-(prop-2-yn-1-yloxy)phenyl)-6-(phenylthio)pyridine-3,5-dicarbonitrile (7a)

Et_3N (140 µL. 1.00 mmol, 0.05 eq) was added dropwise to a suspension of **6a** (3.80 g, 20.0 mmol, 1.0 eq) and malononitrile (2.73 g, 41.3 mmol, 2.1 eq) in EtOH (30 mL). The mixture was brought to 50°C. upon which thiophenol (2.18 ml, 21.3 mmol, 1.1 eq) was added. The mixture was then stirred at 50°C for 4 h, after which TLC revealed full consumption of the aldehyde. The mixture was cooled down to rt and the formed precipitate was collected by filtration to yield **7a** (3.87 g, 9.38 mmol, 47%) as an off-white solid.

TLC (PE/EtOAc 7:3): $R_f = 0.27$.

^{1}H NMR (400 MHz, $(CD_3)_2SO$): δ [ppm] = 7.78 (s, 2H), 7.63 – 7.58 (m, 2H), 7.55 – 7.46 (m, 3H), 7.28 – 7.23 (m, 1H), 7.21 – 7.11 (m, 2H), 4.90 (d, *J* = 1.8 Hz, 2H), 3.81 (s, 3H), 3.64 (t, *J* = 2.1 Hz, 1H).

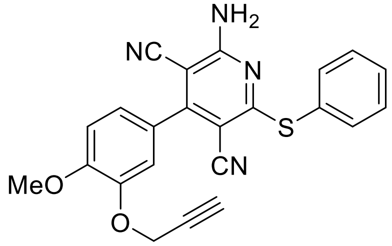

2-Amino-4-(4-methoxy-3-(prop-2-yn-1-yloxy)phenyl)-6-(phenylthio)pyridine-3,5-dicarbonitrile (7b)

Malononitrile (264 mg, 3.99 mmol, 1.0 eq) and piperidine (catalytic amount) were added to a solution of **6b** (759 mg, 3.99 mmol, 1.0 eq) in EtOH (5 mL) at 50 °C. The mixture immediately became a yellow suspension. The mixture was refluxed at 50 °C for 5 h and afterwards cooled down to rt. The formed precipitate was filtrated and dried under reduced pressure to yield 611 mg (2.56 mmol) of the malononitrile-substituted intermediate. This crude intermediate was then re-dissolved in EtOH (10 mL) by heating the mixture to 65 °C. Malononitrile (190 mg, 2.88 mmol, 1.4 eq) and triethylamine (catalytic amount) were added and the solution was refluxed for 45 min at 65 °C. Thiophenol (288 µL, 2.82 mmol, 0.7 eq) was added and the solution was refluxed for 3 h at 65 °C, upon which TLC showed full consumption of the starting material. The mixture was cooled down to rt and allowed to crystallize overnight. The formed precipitate was collected by filtration and dried under reduced pressure to yield **7b** (500 mg, 1.21 mmol, 30% yield).

TLC (PE/EtOAc 7:3): R_f = 0.22.
^{1}H NMR (300 MHz, $CDCl_3$): δ [ppm] = 7.59 – 7.52 (m, 2H), 7.50 – 7.44 (m, 3H), 7.25 – 7.20 (m, 2H), 7.04 (d, *J* = 9.0 Hz, 1H), 5.43 (s, 2H), 4.83 (d, *J* = 2.4 Hz, 2H), 3.95 (s, 3H), 2.59 (t, *J* = 2.4 Hz, 1H).
LCMS (ESI, m/z): $[M+H]^+$: 413.15.
HPLC: 100%, RT 10.893 min.

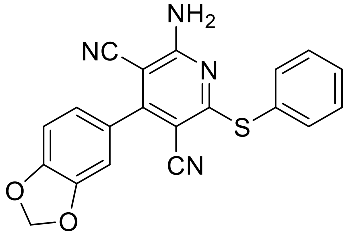

2-Amino-4-(benzo[d][1,3]dioxol-5-yl)-6-(phenylthio)pyridine-3,5-dicarbonitrile (7c)
Piperonal (3.00 g, 20.0 mmol, 1 eq) was added to a solution of malononitrile (1.32 g, 20.0 mmol, 1.0 eq) in EtOH (20 ml). Piperidine (catalytic amount) was added and the solution was refluxed at 75 °C for 3 h, before slowly cooling down to RT. The formed yellow precipitate was collected by filtration, washed with EtOH and water and dried under reduced pressure to yield the malononitrile-substituted intermediate (3.72 g, 18.80 mmol). 1 gram of the crude intermediate was taken for the further steps (1.00 g, 5.05 mmol, 1.0 eq) and dissolved in EtOH (10 ml). Malononitrile (364mg, 5.51 mmol, 1.1 eq), thiophenol (567 µL, 5.56 mmol, 1.1 eq) and triethylamine (catalytic amount) were added and the mixture was refluxed at 75 °C for 10 h before slowly cooling down to rt. The formed yellow precipitate was collected by filtration and the residue was purified by automated flash column chromatography (DCM/MeOH 99:1 → 95:5). The precipitate and the purified filtrate were combined to yield **7c** (885 mg, 2.38 mmol, 44% over two steps) as a yellow solid.
TLC (PE/EtOAc 7:3): R_f = 0.27.
^{1}H NMR (300 MHz, $CDCl_3$): δ [ppm] = 7.58 – 7.52 (m, 2H), 7.50 – 7.43 (m, 3H), 7.04 (dd, *J* = 8.0, 1.8 Hz, 1H), 6.99 (d, *J* = 1.7 Hz, 1H), 6.96 (d, *J* = 8.0 Hz, 1H), 6.08 (s, 2H), 5.43 (s, 2H).
LCMS (ESI, m/z): $[M+H]^+$: 373.10.
HPLC: 100%, RT 10.924 min.

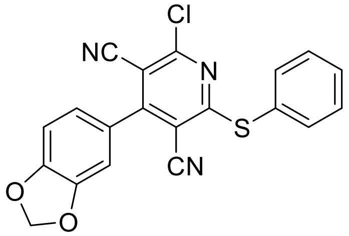

4-(Benzo[d][1,3]dioxol-5-yl)-2-chloro-6-(phenylthio)pyridine-3,5-dicarbonitrile (8)
Isopentyl nitrite (403 µl, 3.00 mmol, 1.4 eq) and copper(II)chloride (402 mg, 2.99 mmol, 1.4 eq) were added to a solution of **7c** (797 mg, 2.14 mmol, 1.0 eq) in acetonitrile (10 ml) The mixture was refluxed at 60 °C for 20 h and afterwards slowly cooled down to rt. 1 M HCl (30 mL) was added and the resulting green aqueous layer was extracted with DCM (4 x 30 mL). The organic layers were combined, dried over $MgSO_4$ and concentrated under reduced pressure. The residue was purified by flash column chromatography (PE/EtOAc 9:1 → 7:3) to yield **8** (606 mg, 1,547 mmol, 72 % yield).
TLC (PE/EtOAc 7:3): R_f = 0.63.
^{1}H NMR (400 MHz, $CDCl_3$): δ [ppm] = 7.64 – 7.44 (m, 5H), 7.06 (dd, *J* = 8.0, 1.9 Hz, 1H), 7.01 – 6.98 (m, 2H), 6.10 (s, 2H).

^{13}**C NMR** (101 MHz, $CDCl_3$) δ 169.3, 158.5, 156.1, 150.8, 148.6, 135.7, 130.8, 129.8, 125.8, 125.2, 123.9, 113.7, 113.4, 109.3, 109.0, 106.3, 105.0, 102.3.
LCMS (ESI, m/z): $[M+H]^+$: 392.00.
HPLC: 100%, RT 11.892 min.

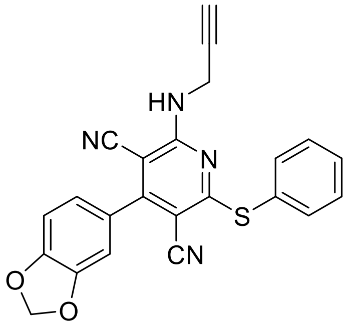

4-(Benzo[d][1,3]dioxol-5-yl)-2-(phenylthio)-6-(prop-2-yn-1-ylamino)pyridine-3,5-dicarbonitrile (9)
Propargyl amine (90 µL, 1.41 mmol, 2 eq) was added to a solution of **8** (274 mg, 0,699 mmol) in dry THF (3.5 ml) and the mixture was stirred at rt overnight. Water (25 mL) was then added and the aqueous layer was extracted with DCM (2 x 25 mL). The organic layers were combined and washed with 1 M HCl (2 x 25 mL) and brine (25 mL), dried over $MgSO_4$, filtered and concentrated under reduced pressure to yield **9** (265 mg, 0.65 mmol, 92%) as a yellow solid.
TLC (Pentane/EtOAc 8:2): $R_f = 0.31$.
^{1}**H NMR** (500 MHz, $CDCl_3$): δ [ppm] = 7.59 (dd, J = 7.9, 1.7 Hz, 2H), 7.53 – 7.41 (m, 3H), 7.01 (dd, J = 8.0, 1.9 Hz, 1H), 6.97 (d, J = 1.8 Hz, 1H), 6.94 (d, J = 8.0 Hz, 1H), 6.05 (s, 2H), 5.83 (t, J = 5.5 Hz, 1H), 3.74 (dd, J = 5.5, 2.5 Hz, 2H), 2.18 (t, J = 2.5 Hz, 1H).
^{13}**C NMR** (126 MHz, $CDCl_3$): δ [ppm] = 169.3, 157.7, 156.9, 150.0, 148.2, 136.2, 130.1, 129.3, 127.2, 126.7, 123.3, 115.3, 115.1, 109.0, 108.9, 102.0, 95.1, 88.1, 78.9, 71.9, 30.9.

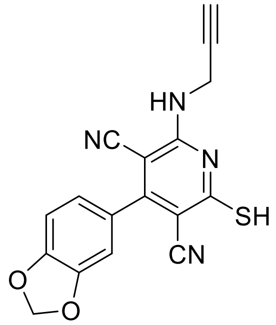

4-(Benzo[d][1,3]dioxol-5-yl)-2-mercapto-6-(prop-2-yn-1-ylamino)pyridine-3,5-dicarbonitrile (10)
Potassium thioacetate (178 mg, 1.56 mmol, 2.0 eq) was added to a solution of **9** (321 mg, 0.78 mmol, 1.0 eq) in dry DMF (4 mL). After 6 h of stirring, 2.0 equivalent of potassium thioacetate (178 mg, 1.56 mmol) was added and the mixture was stirred for another 2 h, upon which the TLC indicated full consumption of starting material. EtOAc (50 mL) was added and the organic layer was washed with brine (3 x 50 mL), dried over $MgSO_4$, filtered and concentrated under reduced pressure to yield **10** as a yellow/brown solid (258 mg, 0.77 mmol, 99%), which was used in the next steps without further purification.
TLC (DCM:MeOH 95:5): $R_f = 0.18$.
^{1}**H NMR** (500 MHz, $(CD_3)_2SO$) δ [ppm] = 8.71 (t, J = 5.6 Hz, 1H), 7.22 (d, J = 1.8 Hz, 1H), 7.18 – 7.09 (m, 2H), 6.21 – 6.11 (m, 3H), 4.08 (dd, J = 5.7, 2.5 Hz, 2H), 2.81 (t, J = 2.4 Hz, 1H).

^{13}C NMR (126 MHz, $(CD_3)_2SO$) δ [ppm] = 162.4, 158.1, 157.0, 149.3, 147.5, 126.7, 123.4, 114.9, 114.6, 109.0, 108.7, 101.9, 93.8, 89.8, 89.1, 72.0, 30.6.
LCMS (ESI, m/z): $[M+H]^+$: 334.95.

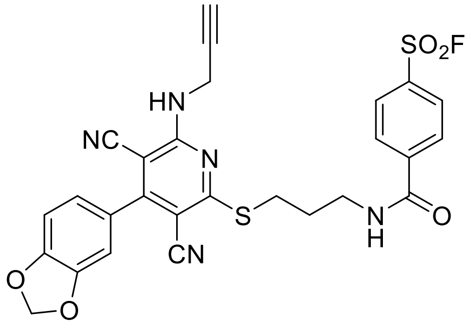

4-((3-((4-(Benzo[d][1,3]dioxol-5-yl)-3,5-dicyano-6-(prop-2-yn-1-ylamino)pyridin-2-yl)thio)propyl)carbamoyl)benzenesulfonyl fluoride (1)
19 (350 mg, 1.08 mmol, 1.4 eq) and $NaHCO_3$ (97 mg, 1.16 mmol, 1.5 eq) were added to a solution of **10** (258 mg, 0.77 mmol, 1.0 eq) in dry DMF (3.4 mL). The mixture was stirred for 2 days, after which another 1.5 eq of $NaHCO_3$ (97 mg, 1.16 mmol) was added. The mixture was then stirred at 50 °C for two days. DCM (50 mL) was added and the organic layer was washed with brine (3 x 50 mL), dried over $MgSO_4$, filtered and concentrated under reduced pressure. The residue was purified by silica column chromatography (DCM:MeOH 99.5:0.5 → 99:1) and recrystallization in DCM to yield **1** as a yellow solid (177 mg, 0.31 mmol, 40%).
TLC (DCM:MeOH 99:1): R_f = 0.38.
^{1}H NMR (500 MHz, $(CD_3)_2SO$) δ [ppm] = 8.95 (t, *J* = 5.6 Hz, 1H), 8.56 (t, *J* = 5.6 Hz, 1H), 8.26 (d, *J* = 8.6 Hz, 2H), 8.19 (d, *J* = 8.5 Hz, 2H), 7.18 (d, *J* = 1.8 Hz, 1H), 7.10 (d, *J* = 8.0 Hz, 1H), 7.05 (dd, *J* = 8.1, 1.8 Hz, 1H), 6.15 (s, 2H), 4.21 (dd, *J* = 5.6, 2.4 Hz, 2H), 3.46 (q, *J* = 6.4 Hz, 2H), 3.38 (t, *J* = 6.9 Hz, 2H), 3.09 (t, *J* = 2.4 Hz, 1H), 2.04 (p, *J* = 6.9 Hz, 2H).
^{13}C NMR (126 MHz, $(CD_3)_2SO$) δ [ppm] = 167.2, 164.5, 157.6, 157.0, 149.0, 147.4, 141.5, 133.4 (d, *J* = 23.8 Hz), 129.1, 128.6, 127.2, 123.1, 115.4, 115.0, 109.0, 108.6, 101.8, 94.4, 87.4, 80.8, 72.8, 38.6, 30.9, 29.1, 27.7.
^{19}F NMR (471 MHz, $(CD_3)_2SO$) δ [ppm] = 65.94.
HRMS (ESI, m/z): $[M+H]^+$, calculated: 578.0963, found: 578.0970.
HPLC: 99%, RT 11.262 min.

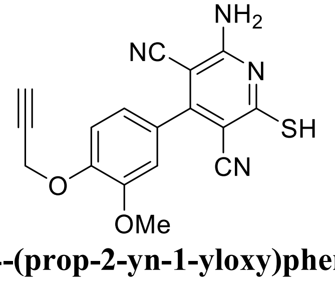

2-Amino-6-mercapto-4-(3-methoxy-4-(prop-2-yn-1-yloxy)phenyl)pyridine-3,5-dicarbonitrile (11a)
Potassium thioacetate (65 mg, 0.57 mmol, 2.4 eq) was added to a solution of **7a** (99 mg, 0.24 mmol, 1.0 eq) in dry DMF (2 mL). The mixture was stirred for 6 h, after which extra potassium thioacetate (65 mg, 0.57 mmol, 2.4 eq) was added. The mixture was stirred overnight and another 2.4 equivalent of potassium thioacetate (65 mg, 0.57 mmol) was added. The mixture was stirred for 4 h, after which full consumption of starting materials was observed by LCMS. 2 M NaOH (5 mL) was added and the mixture was stirred at rt over the weekend. Water (10 mL) and 1 M HCl (10 mL) were then added. Immediately a yellow precipitate formed, which was collected by extraction with EtOAc (3 x 20 mL).

The organic layers were combined, dried over $MgSO_4$, filtered and concentrated under reduced pressure to yield **11a** (60 mg, 0.18 mmol, 74%) a yellow solid, which was used in the next steps without further purification.
TLC (DCM:MeOH 95:5): R_f = 0.15.
^{1}H NMR (500 MHz, $(CD_3)_2SO$) δ [ppm] = 12.98 (s, 1H), 7.95 (s, 2H), 7.20 – 7.17 (m, 1H), 7.16 (s, 1H), 7.09 (dd, *J* = 8.4, 2.1 Hz, 1H), 4.89 (d, *J* = 2.4 Hz, 2H), 3.80 (s, 4H), 3.62 (t, *J* = 2.4 Hz, 1H).
^{13}C NMR (126 MHz, $(CD_3)_2SO$) δ [ppm] = 179.3, 158.4, 154.5, 148.5, 148.2, 127.0, 120.9, 116.8, 114.8, 113.0, 112.3, 102.4, 81.6, 79.0, 78.7, 55.9, 55.8.
LCMS (ESI, m/z): $[M+H]^+$: 337.00.

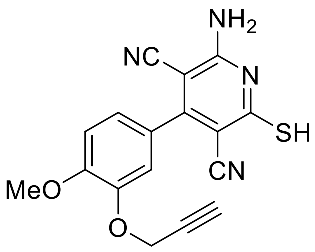

2-Amino-6-mercapto-4-(4-methoxy-3-(prop-2-yn-1-yloxy)phenyl)pyridine-3,5-dicarbonitrile (11b)
Potassium thioacetate (276 mg, 2.42 mmol, 2.0 eq) was added to a solution of **7b** (500 mg, 1,21 mmol, 1.0 eq) in dry DMF (6 mL). The mixture was stirred at rt overnight and the next day another equivalent of potassium thioacetate (138 mg, 1.21 mmol, 1 eq) was added. The mixture was stirred over 3 days, upon which no starting material was visible anymore on TLC. 2 M NaOH (6 mL) was added to hydrolyze the formed thioacetate. After stirring for 8 h at rt, another 6 mL of 2 M NaOH was added and the mixture was stirred overnight. Water (62 mL) and 1 M HCl (24 mL) were added to acidify the mixture. The product was allowed to crystallize overnight. The formed precipitate was collected by filtration, dried under reduced pressure and recrystallized in a mixture of EtOH and MeOH to yield **11b** (253 mg, 0.75 mmol, 62%) as a yellow solid.
TLC (DCM/MeOH 95:5): R_f = 0.17.
^{1}H NMR (400 MHz, CD_3OD): δ [ppm] = 7.30 – 7.09 (m, 3H), 4.79 (d, *J* = 2.5 Hz, 2H), 3.92 (s, 3H), 2.97 (t, *J* = 2.4 Hz, 1H).

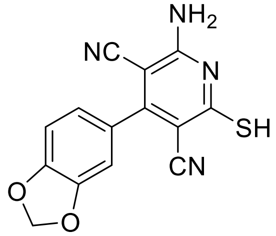

2-Amino-4-(benzo[d][1,3]dioxol-5-yl)-6-mercaptopyridine-3,5-dicarbonitrile (11c)
Potassium thioacetate (863 mg, 7.56, mmol, 2.0 eq) was added to **7c** (1409 mg, 3.78 mmol, 1.0 eq) in dry DMF (19 mL). The solution was stirred at rt overnight, after which no starting material was observed anymore by TLC. Therefore 2 M NaOH (20 mL) was added to hydrolyze the formed thioacetate and the mixture was stirred for 7 h. Water (20 mL) was added to quench the and 1 M HCl (40 mL) was slowly added to bring the solution to a pH of 5. Upon addition, a yellow precipitate started to form. Another 80 mL of water was then added and the product was crystallized over 3 days. Afterwards the crystals were collected by filtration and re-crystallized in EtOH and MeOH to yield **11c** (877 mg, 2.96 mmol, 78%) as a yellow solid.
TLC (DCM/MeOH 95:5): R_f = 0.23.
^{1}H NMR (400 MHz, CD_3OD): δ [ppm] = 7.12 – 6.89 (m, 3H), 6.07 (s, 2H).

LCMS (ESI, m/z): $[M+H]^+$: 296.95.
HPLC: 84%, RT 8.200 min.

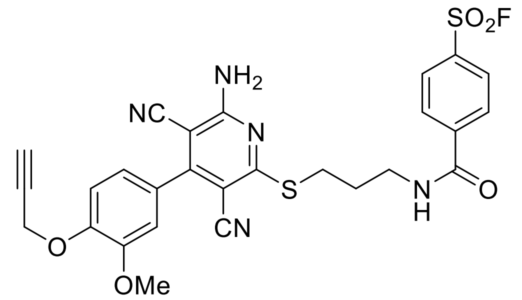

4-((3-((6-Amino-3,5-dicyano-4-(3-methoxy-4-(prop-2-yn-1-yloxy)phenyl)pyridin-2-yl)thio)propyl)carbamoyl)benzenesulfonyl fluoride (2)
19 (50 mg, 0.15 mmol, 1.4 eq) and $NaHCO_3$ (19 mg, 0.23 mmol, 1.5 eq) were added to a solution of **11a** in dry DMF (2 mL) and the mixture was stirred overnight. DCM was added and the organic layer was washed with brine (3 x 30 mL), dried over $MgSO_4$, filtered and concentrated. The residue was purified by silica column chromatography (DCM:MeOH 99.5:0.5 → 95:5) and recrystallization in DCM to yield **2** as a white solid (46 mg, 0.08 mmol, 53% yield).
TLC (DCM:MeOH 99:1): $R_f = 0.30$.
^{1}H NMR (500 MHz, $(CD_3)_2SO$) δ [ppm] = 8.93 (t, J = 5.6 Hz, 1H), 8.26 (d, J = 8.6 Hz, 2H), 8.18 (d, J = 8.5 Hz, 2H), 7.97 (s, 2H), 7.22 (d, J = 2.1 Hz, 1H), 7.18 (d, J = 8.4 Hz, 1H), 7.10 (dd, J = 8.3, 2.1 Hz, 1H), 4.89 (d, J = 2.4 Hz, 2H), 3.81 (s, 3H), 3.62 (t, J = 2.4 Hz, 1H), 3.45 (q, J = 6.5 Hz, 2H), 3.30 (t, J = 7.0 Hz, 2H), 1.97 (p, J = 6.9 Hz, 2H).
^{13}C NMR (126 MHz, $(CD_3)_2SO$) δ [ppm] = 167.0, 164.6, 159.8, 157.9, 148.6, 148.1, 141.5, 133.4 (d, J = 23.7 Hz), 129.1, 128.6, 126.8, 121.2, 115.7, 115.5, 113.2, 112.6, 93.7, 85.7, 79.0, 78.7, 55.9, 55.8, 38.5, 28.6, 27.3.
^{19}F NMR (471 MHz, $(CD_3)_2SO$) δ [ppm] = 65.94.
HRMS (ESI, m/z): $[M+H]^+$, calculated: 580.1117, found: 580.1119.
HPLC: 95%, RT 10.910 min.

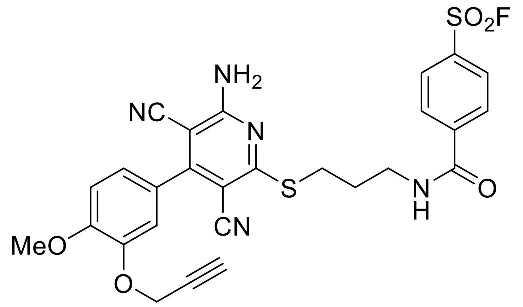

4-((3-((6-Amino-3,5-dicyano-4-(4-methoxy-3-(prop-2-yn-1-yloxy)phenyl)pyridin-2-yl)thio)propyl)carbamoyl)benzenesulfonyl fluoride (3)
$NaHCO_3$ (43 mg, 0.51 mmol, 1.5 eq) and **19** (150 mg, 0.46 mmol, 1.4 eq) in dry DMF (3 ml) were added to **11b** (115 mg, 0.34 mmol, 1.0 eq) and the mixture was stirred for two days at RT. DCM (20 mL) was then added and the mixture was washed with water (20 mL), dried over $MgSO_4$, filtered and concentrated under reduced pressure. The residue was purified by flash column chromatography (Pentane/EtOAc 1:1 → 1:4) to yield **3** (60 mg, 0.10 mmol, 29%) as an off-white solid.
TLC (Pentane/EtOAc 7:3): $R_f = 0.31$.

^{1}H NMR (500 MHz, $CDCl_3$): δ [ppm] = 8.08 – 8.00 (m, 4H), 7.20 – 7.16 (m, 2H), 7.02 (d, *J* = 8.1 Hz, 1H), 6.99 (t, *J* = 6.0 Hz, 1H), 6.04 (s, 2H), 4.80 (d, *J* = 2.4 Hz, 2H), 3.59 (q, *J* = 6.6 Hz, 2H), 3.24 (t, *J* = 7.1 Hz, 2H), 2.59 (t, *J* = 2.4 Hz, 1H), 2.10 (p, *J* = 7.1 Hz, 2H).
^{13}C NMR (126 MHz, $CDCl_3$): δ [ppm] = 168.6, 165.7, 159.7, 157.6, 152.0, 146.9, 141.0, 135.4 (d, *J* = 25.1 Hz), 128.8, 128.5, 125.3, 123.3, 115.7, 115.6, 114.7, 111.8, 96.1, 86.7, 78.0, 77.0, 57.2, 56.1, 39.6, 28.8, 28.1.
^{19}F NMR (471 MHz, $CDCl_3$): δ [p5pm] = 65.72.
HRMS (ESI, m/z): $[M+H]^+$, calculated: 580.1119, found: 580.1113.
HPLC: 96%, RT 10.861 min.

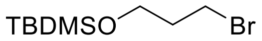

(3-Bromopropoxy)(tert-butyl)dimethylsilane (13)
A solution of TBDMS-Cl (50 wt% in toluene)(7.83 mL, 22.50 mmol, 1.5 eq) was added to a solution of 3-bromopropanol(1.36 ml, 15.00 mmol) in dry DMF (10 mL). 1H-imidazole (2.04 g, 30.00 mmol) was added the mixture was stirred for 5 h. DCM (100 mL) was then added and the mixture was washed with water (3 x 100 mL) and brine (1 x 100 mL), dried over $MgSO_4$ and concentrated under reduced pressure to yield crude **13** (7.60 g, 30.00 mmol, quant).
^{1}H NMR (300 MHz, $CDCl_3$): δ [ppm] = 3.73 (t, *J* = 5.7 Hz, 2H), 3.51 (t, *J* = 6.4 Hz, 2H), 2.04 (p, *J* = 6.5, 6.1 Hz, 2H), 0.89 (s, 9H), 0.06 (s, 6H).
^{13}C NMR (75 MHz, $CDCl_3$): δ [ppm] = 60.5, 35.7, 30.8, 26.0, 25.8, -5.2.

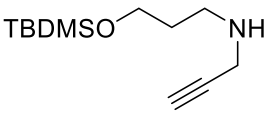

N-(3-((*tert*-Butyldimethylsilyl)oxy)propyl)prop-2-yn-1-amine (14)
A solution of **13** (3.80 g, 15.00 mmol, 1.0 eq) and DIPEA (5.22 mL, 30.00 mmol, 2.0 eq) in acetonitrile (15 mL) was added dropwise to propargylamine (4.80 ml, 75.00 mmol, 3 eq) over a time period of 5 h using a syringe pump. After 5 h, the mixture had turned orange and LCMS analysis revealed mono- and disubstituted product. EtOAc (200 mL) was added and the mixture was washed with brine (3 x 150 mL), dried over $MgSO_4$, filtered and concentrated under reduced pressure. The residue was purified by flash column chromatography (Pentane/EtOAc 6:4 → 4:6) to yield **14** (1.78 g, 7.81 mmol, 52%) as a brown oil.
TLC (EtOAc): R_f = 0.48.
^{1}H NMR (400 MHz, $CDCl_3$): δ [ppm] = 3.70 (t, *J* = 6.1 Hz, 2H), 3.42 (d, *J* = 2.4 Hz, 2H), 2.78 (t, *J* = 6.8 Hz, 2H), 2.20 (t, *J* = 2.4 Hz, 1H), 1.71 (p, *J* = 6.4 Hz, 2H), 0.89 (s, 9H), 0.05 (s, 6H).
^{13}C NMR (101 MHz, $CDCl_3$): δ [ppm] = δ 82.4, 71.3, 61.9, 46.3, 38.4, 32.8, 28.2, 18.4, -3.3.
LCMS (ESI, m/z): $[M+H]^+$: 228.10.

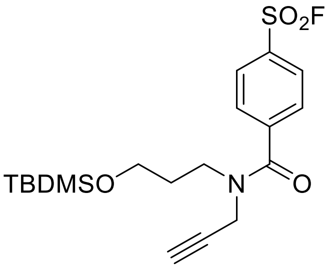

4-((3-((*tert*-Butyldimethylsilyl)oxy)propyl)(prop-2-yn-1-yl)carbamoyl)benzenesulfonyl fluoride (15)

EDC·HCl (1.65 g, 8.59 mmol, 1.1 eq) and **14** (1776 mg, 7,81 mmol, 1.0 eq) in dry DMF (10 mL) were added to a solution of 4-fluorosulfonyl benzoic acid (1.75 g, 8.59 mmol, 1.1 eq) in dry DMF (10 mL) and the mixture was stirred at rt. After stirring for 1 h, DIPEA (2.72 mL, 15.62 mmol, 2.0 eq) was added and the mixture was stirred overnight. The next day additional DIPEA (1.36 mL, 7.81 mmol, 1.0 eq) and **2** (616 mg, 3.02 mmol, 0.4 eq) were added and the mixture was stirred overnight. DIPEA (1.36 mL, 7.81 mmol, 1.0 eq) was added and the mixture was stirred for another night. As no further progress of the reaction was observed, water (100 mL) was added and the aqueous layer was extracted with DCM (100 mL). The organic layer was washed with water (2 x 100 mL) and the aqueous layers were combined and back-extracted with DCM (100 mL). The organic layers were combined, washed with brine (100 mL), dried over $MgSO_4$, filtered and concentrated under reduced pressure. The residue was purified by flash column chromatography (Pentane/EtOAc 95:5 → 75:25) to yield yellow oil **15** (1.88 mg, 4.53 mmol, 58%) as a mixture of two rotamers, as determined by ^{1}H NMR, ^{13}C NMR, ^{19}F NMR and LCMS measurements.
TLC (Pentane/EtOAc 8:2): $R_f = 0.77$.
^{1}H NMR (500 MHz, $CDCl_3$): δ [ppm] = 8.08 – 7.92 (m, 2H), 7.66 (dd, *J* = 49.1, 8.0 Hz, 2H), 4.11 (d, *J* = 216.9 Hz, 2H), 3.78 – 3.54 (m, 2H), 3.45 (dt, *J* = 37.9, 6.6 Hz, 2H), 2.31 (d, *J* = 43.6 Hz, 1H), 1.83 (d, *J* = 79.6 Hz, 2H), 0.94 – 0.55 (m, 9H), 0.14 – -0.25 (m, 6H).
^{13}C NMR (126 MHz, $CDCl_3$): δ [ppm] = 168.8, 168.5, 143.2, 143.1, 133.9, 133.7, 128.7, 128.0, 78.1, 73.6, 72.5, 60.5, 59.6, 46.0, 43.3, 39.5, 34.0, 31.2, 30.8, 30.3, 25.9, 25.9, 25.7, 25.6, -5.4, -5.6.
^{19}F NMR (471 MHz, $CDCl_3$): δ [ppm] = 65.81, 65.71.
LCMS (ESI, m/z): $[M+H]^+$: 414.05.
HPLC: 99%, RT 10.437 min.

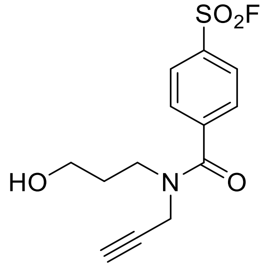

4-((3-Hydroxypropyl)(prop-2-yn-1-yl)carbamoyl)benzenesulfonyl fluoride (16)
$Et_3N·3HF$ (1.01 mL, 6.18 mmol, 6 eq) was added to a solution of **15** (426 mg, 1,03 mmol, 1.0 eq) in dry THF (5 mL). The mixture was stirred overnight at room temperature, after which DCM (50 mL) was added. The mixture was then washed with brine (2 x 50 mL). The aqueous layers were combined and back-extracted with DCM (50 mL). The organic layers were combined and concentrated under reduced pressure. The residue was purified by flash column chromatography (DCM/MeOH 99.5:0.5 → 98:2) to yield **16** (283 mg, 0.95 mmol, 92%) as a colorless oil.
TLC (DCM/MeOH 98:2): $R_f = 0.38$.
^{1}H NMR (400 MHz, $CDCl_3$): δ [ppm] = 8.06 (d, *J* = 8.1 Hz, 2H), 7.81 – 7.58 (m, 2H), 3.98 – 3.81 (m, 2H), 3.76 (t, *J* = 6.5 Hz, 2H), 3.65 – 3.47 (m, 2H), 2.51 – 2.27 (m, 1H), 1.87 (p, *J* = 6.1 Hz, 2H).
^{13}C NMR (101 MHz, $CDCl_3$): δ [ppm] = δ 169.8, 142.3, 134.3 (d, *J* = 25.0 Hz), 128.9, 128.1, 77.9, 74.2, 58.8, 42.6, 39.5, 29.7.
^{19}F NMR (471 MHz, $CDCl_3$): δ [ppm] = 65.70.
LCMS (ESI, m/z): $[M+H]^+$: 299.95.
HPLC: 100%, RT 8.158 min.

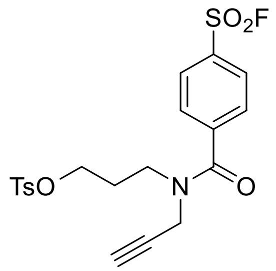

3-(4-(Fluorosulfonyl)-N-(prop-2-yn-1-yl)benzamido)propyl 4-methylbenzenesulfonate (17)

TsCl (906 mg, 4.75 mmol, 5.0 eq) and Et_3N (264 µL, 1.90 mmol, 2.0 eq) were added to a solution of **16** (283 mg, 0.95 mmol, 1.0 eq) in dry DMF (5 mL). The mixture was stirred for two days at rt, after which another 2 equivalents of Et_3N (264 µL, 1.90 mmol, 2.0 eq) were added. The mixture was stirred overnight and 2 equivalents of Et_3N (264 µL, 1.90 mmol, 2.0 eq) were added. The mixture was stirred for another 2 h and afterwards diluted with DCM (25 mL). The organic layer was washed with H_2O (25 mL) and brine (25 mL), dried over $MgSO_4$, filtered and concentrated under reduced pressure. The residue was purified by flash column chromatography (Pentane/Et_2O 7:3 → 3:7) to yield the colorless oil **17** (238 mg, 0.52 mmol, 55%) as a mixture of two rotamers, as determined by ^{1}H NMR, ^{13}C NMR, ^{19}F NMR and LCMS measurements.

TLC (Pentane/EtOAc 1:1): R_f = 0.46.

^{1}H NMR (500 MHz, $CDCl_3$)* δ [ppm] = 8.17 (d, *J* = 8.2 Hz, 2H), 7.96 (d, *J* = 8.5 Hz, 2H), 7.69 (d, *J* = 7.9 Hz, 2H), 7.11 (d, *J* = 7.9 Hz, 2H), 4.40 (t, *J* = 5.9 Hz, 2H), 4.05 – 3.95 (m, 2H), 3.37 – 3.25 (m, 2H), 2.51 (t, *J* = 2.5 Hz, 1H), 2.46 – 2.41 (m, 2H), 2.30 (s, 3H).

^{13}C NMR (126 MHz, $CDCl_3$)* δ [ppm] = 164.3, 142.6, 141.1, 136.7 (d, *J* = 25.3 Hz), 136.2, 130.9, 129.1, 128.5, 125.9, 78.1, 73.3, 62.6, 43.7, 36.9, 25.3, 21.4.

^{19}F NMR (471 MHz, $CDCl_3$)* δ [ppm] = 65.44.

LCMS (ESI, m/z): $[M+H]^+$: 453.95.

HPLC: 96%, RT 10.945 min.

*Only the values of the most abundant rotamer are given.

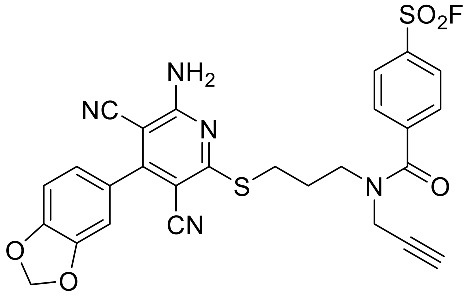

4-((3-((6-Amino-4-(benzo[d][1,3]dioxol-5-yl)-3,5-dicyanopyridin-2-yl)thio)propyl)(prop-2-yn-1-yl)carbamoyl)benzenesulfonyl fluoride (LUF7909) (4)

11 (238 mg, 0.52 mmol, 1.0 eq) in dry DMF (3 mL) was added to **14** (231 mg, 0.80 mmol, 1.5 eq). $NaHCO_3$ (44 mg, 0.52 mmol, 1.0 eq) was added and the mixture was stirred overnight at rt. The mixture was then diluted with DCM (25 mL). The organic layer was washed with brine (4 x 25 mL), dried over $MgSO_4$, filtered and concentrated under reduced pressure. The residue was purified by flash column chromatography (Pentane/Et_2O 4:6 → 1:9) to yield **3** (90 mg, 0.16 mmol, 31%) as an off-white solid.

TLC (Pentane/EtOAc 1:1): R_f = 0.47.

^{1}H NMR (500 MHz, $CDCl_3$, 20 °C): δ [ppm] = 8.09 (d, *J* = 7.8 Hz, 2H), 7.87 – 7.61 (m, 2H), 7.00 (dd, *J* = 8.0, 1.7 Hz, 1H), 6.95 (d, *J* = 1.7 Hz, 1H), 6.93 (d, *J* = 8.0 Hz, 1H), 6.28 – 5.82 (m, 4H), 4.20 (d, *J* = 256.2 Hz, 2H), 3.68 (d, *J* = 99.6 Hz, 2H), 3.12 (d, *J* = 114.7 Hz, 2H), 2.54 – 2.31 (m, 1H), 2.31 – 2.13 (m, 2H).

^{1}**H NMR** (500 MHz, $CDCl_3$, 59 °C): δ [ppm] = 8.08 (d, *J* = 7.9 Hz, 2H), 7.74 (d, *J* = 7.8 Hz, 2H), 7.00 (dd, *J* = 8.0, 1.8 Hz, 1H), 6.96 (d, *J* = 1.8 Hz, 1H), 6.93 (d, *J* = 8.0 Hz, 1H), 6.05 (s, 2H), 5.93 (s, 2H), 4.01 (s, 2H), 3.73 (s, 2H), 3.20 (s, 2H), 2.45 – 2.27 (m, 1H), 2.27 – 1.92 (m, 2H).
^{13}**C NMR** (126 MHz, $CDCl_3$): δ [ppm] = 169.1, 168.7, 159.7, 157.8, 150.0, 148.3, 142.5, 134.5 (d, *J* = 25.7 Hz), 129.1, 128.1, 126.8, 123.3, 115.6, 115.2, 109.0, 108.9, 102.0, 96.4, 86.7, 78.0, 74.4, 45.9, 40.1, 28.3, 27.0.
^{19}**F NMR** (471 MHz, $CDCl_3$): δ [ppm] = 65.78.
HRMS (ESI, m/z): $[M+H]^+$, calculated: 578.0963, found: 578.1054.
HPLC: 96%, RT 11.122 min.

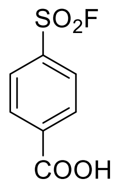

4-(Fluorosulfonyl)benzoic acid (18)
A solution of potassium bifluoride (2.34 g, 30,0 mmol, 3 eq) in water (20 mL) (1.5 M) was added to a solution of 4-(chlorosulfonyl)benzoic acid (2.21 g, 10,0 mmol, 1 eq) in dioxane (25 mL). The mixture was stirred for 3 h, after which TLC and LCMS showed full consumption of starting material. EtOAc (120 mL) was added and the organic layer was washed with water (2 x 150 mL) and brine (1 x 100 mL). The aqueous layers were combined and back-extracted with EtOAc (100 mL). The organic layers were collected, dried over $MgSO_4$, filtered and concentrated under reduced pressure to yield **18** (1.93 g, 9,47 mmol, 95%) as a white solid.
TLC (PE/EtOAc 3:2 + 1% AcOH): R_f = 0.34.
^{1}**H-NMR** (500 MHz, CD_3OD): δ [ppm] = 8.33 (d, *J* = 7.9 Hz, 2H), 8.18 (d, *J* = 8.3 Hz, 2H).
^{19}**F NMR** (471 MHz, CD_3OD): δ [ppm] = 63.37.

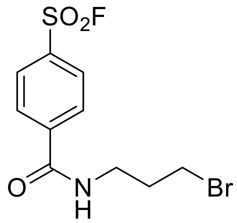

4-((3-Bromopropyl)carbamoyl)benzenesulfonyl fluoride (19)
3-bromopropylamine hydrobromide (2.18 g, 10.0 mmol, 1.3 eq) and PyBrop (4.26 g, 9.1 mmol, 1.2 eq) were added to a solution of **18** (1.55 g, 7.6 mmol, 1 eq) in anhydrous DMF (1 mL) under N_2. DiPEA (2.7 ml, 15.0 mmol, 2 eq) was added dropwise and the mixture was stirred at room temperature. After 3 days of stirring, TLC indicated starting material to be still present. Therefore an additional 0.6 eq of PyBrop (2,13 g, 4.6 mmol) and 1 eq of DiPEA (1.3 mL, 7.6 mmol) were added and the mixture was stirred for another 9 days at rt. The mixture was then diluted with EtOAc (350 mL) and the organic layer was washed brine (350 mL) and water (2 x 350 mL), dried over MgSO4, filtrated and concentrated under reduced pressure. The residue was purified by flash column chromatography (PE/EtOAc 2:1) to yield **19** (1.00 g, 3.1 mmol, 41%) as a white solid.
TLC (PE/EtOAc 2:1): R_f = 0.57.
^{1}**H NMR** (400 MHz, $CDCl_3$): δ [ppm] = 8.10 (d, *J* = 8.5 Hz, 2H), 8.01 (d, *J* = 8.0 Hz, 2H), 6.45 (s, 1H), 3.68 (q, *J* = 6.5 Hz, 2H), 3.51 (t, *J* = 6.3 Hz, 2H), 2.25 (p, *J* = 6.5 Hz, 2H).
^{19}**F NMR** (471 MHz, CDCl3): δ [ppm] = 65.75.

Computational Modelling

All calculations were performed in the Schrödinger Suite (release 2019-1) using the standard settings in Maestro (version 11.9).[1] The crystal structure of adenosine-bound human A_1AR (PDB: 6D9H) was used for docking LUF7909. The protein and ligand were prepared for docking using the protein preparation tool and LigPrep tool, respectively. Adenosine was removed and induced fit docking was performed to dock LUF7909. Docking poses were compared to the binding pose of LUF5833 in the human A_{2A}AR (PDB: 7ARO) using the superposition tool. The pose with the lowest scoring RMSD was further used in covalent docking calculations.[2] A custom reaction type was made to allow nucleophilic substitution of the phenolic OH onto the fluorosulfonyl group. This contained the following lines of code:

```
RECEPTOR_SMARTS_PATTERN 2,[c;r6]-[S,O;H1,-1]

RECEPTOR_SMARTS_PATTERN 2,[C]-[N;H2,H3]

LIGAND_SMARTS_PATTERN 1,[*][F,Cl,Br,I]

CUSTOM_CHEMISTRY ("<1>",("charge",0,(1)))

CUSTOM_CHEMISTRY ("<1>|<2>",("bond",1,(1,2)))

CUSTOM_CHEMISTRY ("<2>[F,Cl,Br,I]",("delete",2))
```

The covalent binding poses of LUF7909 were compared to the binding pose of LUF5833 in the human A_{2A}AR using the superposition tool, as well as evaluated by visual inspection.

General Biology

Cell lines

Chinese hamster ovary (CHO) cells stably expressing the human adenosine A_1 receptor (CHOhA_1AR) were kindly provided by Prof. S.J. Hill (University of Nottingham, UK). Human embryonic kidney 293 cells stably expressing the human adenosine A_{2A} receptor (HEK293hA_{2A}AR) were kindly provided by Dr. J. Wang (Biogen/IDEC, Cambridge, MA). CHO-spap cells stably expressing the wildtype (WT) hA_{2B} receptor (CHO-spap-hA_{2B}AR) were kindly provided by S.J. Dowell (Glaxo Smith Kline, UK). CHO cells stably expressing the human adenosine A_3 receptor (CHOhA_3AR) were a kindly provided by Dr. K.N. Klotz (University of Würzburg, Germany).

Radioligands

[3H]1,3-dipropyl-8-cyclopentyl-xanthine ([^{3}H]DPCPX, specific activity 137 Ci/mmol) and [^{3}H]4-(-2-[7-amino-2-(furan-2-yl)-[1,2,4]triazolo[1,5-a][1,3,5]triazin-5-ylamino)ethyl) phenol ([^{3}H]-ZM241385, specific activity 50 Ci/mmol) were purchased from ARC Inc. (St. Louis, USA). [^{3}H]8-(4-(4-(4-Chlorophenyl)piperazide-1-sulfonyl)phenyl)-1-propylxanthine ([^{3}H]PSB-603, specific activity 79 Ci/mmol) was purchased from Quotient Bioresearch. [^{3}H]8-Ethyl-4-methyl-2-phenyl-(8R)-4,5,7,8-tetrahydro-1H-imidazo[2,1-i]-purin-5-one ([^{3}H]PSB-11, specific activity 56 Ci/mmol) was obtained with the kind help of Prof. C.E. Müller (University of Bonn, Germany). [^{35}S]-guanosine 5'-(γ-thio)triphosphate ([^{35}S]GTPγS, specific activity 1250 Ci/mmol) was purchased from PerkinElmer.

Chemicals

5'-N-ethylcarboxamidoadenosine (NECA), N6-Cyclopentyladenosine (CPA) and adenosine deaminase (ADA) were purchased from Sigma Aldrich. 4-(-2-[7-amino-2-(furan-2-yl)-[1,2,4]triazolo[1,5-a][1,3,5]triazin-5-ylamino)ethyl) phenol (ZM241385) was a gift from Dr. S.M. Poucher (Astra Zeneca, Manchester, UK). 3-[4-[2-[[6-amino-9-[(2R,3R,4S,5S)-5-(ethylcarbamoyl)-3,4-dihydroxy-oxolan-2-yl]purin-2-yl]amino]ethyl]phenyl] propanoic acid (CGS21680) was purchased from Ascent Scientific (Bristol, UK). Bongkrekic acid was obtained from Enzo Life Sciences (cat # BML-CM113). Protease inhibitor cocktail was purchased from Sigma Aldrich (cat # p8340). Bicinchoninic acid (BCA) protein assay reagents were obtained from Pierce Chemical Company (Rockford, IL, USA).[3] AF647-N_3 was obtained from Jena Bioscience, Azide-Fluor-545 and Biotin-PEG3-Azide were ordered from Sigma Aldrich. Pierce™ Avidin agarose beads (cat # 11846734) and Pierce™ ECL Western Blotting Substrate (cat # 32209) were ordered from Thermo Scientific. All other chemicals were of analytical grade and obtained from standard commercial sources.

Biologicals

Collagenase type I was purchased from Sigma Aldrich (Cat # C0130), PNGase was purchased from Promega (cat # V4831), Chymotrypsin was ordered from Promega (cat # V1062) and Enolase digest was ordered from Waters (cat # 186002325). RabbitαratA_1AR antibody was ordered from Sigma Aldrich (cat # A268) and goatαrabbit-HRP antibody was purchased from Jackson ImmunoResearch Laboratories (cat # 115-035-003). Bovine Serum Albumin (BSA) was purchased from Acros Organics (cat # 268131000).

Fat pads

Gonadal fat pads from female mice were kindly donated by prof. Patrick C.M. Rensen and dr. Milena Schönke and obtained from APOE*3-Leiden.CETP mice (C57Bl/6J background). Mice were between 28 and 34 weeks old and 19.9-25.3 g at the time of sacrifice. All mouse experiments were performed in accordance with the Institute for Laboratory Animal Research Guide for the Care and Use of Laboratory

Animals after approval from the Central Animal Experiments Committee ("Centrale Commissie Dierproeven").

Cell culture and membrane preparation

CHO cells were grown in Dulbecco's Modified Eagle's Medium (DMEM) and Ham's F12 medium (1:1) supplemented with 10% (v/v) newborn calf serum 50 µg/mL streptomycin, 50 IU/mL penicillin and 5% CO_2. CHO cells were subcultured twice a week at a ratio of 1:15 on 10 cm Ø plates. CHOhA_1AR and CHOhA3AR cells were grown in DMEM and Ham's F12 medium (1:1) supplemented with 10% (v/v) newborn calf serum, 50 µg/mL streptomycin, 50 IU/mL penicillin, and 200 µg/mL G418 at 37 °C and 5% CO_2. CHOhA_1AR cells were subcultured twice a week at a ratio of 1:20 on 10 cm Ø plates. CHOhA_3AR cells were subcultured twice a week at a ratio of 1:8 on 10 cm Ø plates. HEK293hA_{2A}AR cells were grown in culture medium consisting of DMEM supplemented with 10% newborn calf serum, 50 µg/mL streptomycin, 50 IU/mL penicillin, and 500 µg/mL G418 at 37 °C and 7% CO_2. Cells were subcultured twice a week at a ratio of 1:8 on 10 cm Ø plates. CHO-spap-hA2BR cells were grown in DMEM and Ham's F12 medium (1:1) supplemented with 10% (v/v) newborn calf serum, 100 µg/mL streptomycin, 100 IU/mL penicillin, 1 mg/mL G418, and 0.4 mg/mL hygromycin at 37 °C and 5% CO_2. Cells were subcultured twice a week at a ratio of 1:20 on 10 cm Ø plates.

All cells were grown to 80-90% confluency and detached from plates by scraping them into 5 mL PBS. Detached cells were collected and centrifuged (5 min, 200 G). The supernatant was removed and the pellets were resuspended in cold Tris-HCl buffer, pH 7.4. A Heidolph Diax 900 homogenizer was used to homogenize the cell suspension. Membranes and the cytosolic fraction were separated by centrifugation in a Beckman Optima LE-80 K ultracentrifuge (Beckman Coulter, Fullerton, CA) (20 min, 100 000 G, 4 °C). The pellet was resuspended in Tris-HCl buffer, and homogenization and centrifugation steps were repeated. Tris-HCl buffer was used to resuspend the pellet, and ADA was added (0.8 U/mL) to break down endogenous adenosine. Membranes were stored in 250 µL and 500 µL aliquots at -80 °C. Total protein concentrations were measured using the BCA method.

Preparation of adipocyte membranes from mouse gonadal fat pads[4–6]

Gonadal fat pads of 6 mice were placed into a 10 Ø petri dish containing Krebs-Ringer-HEPES (KRH) buffer (100 mM NaCl, 4.7 mM KCl, 2.5 mM $CaCl_2$, 3.6 mM $NaHCO_3$, 1.19 mM $MgSO_4$, 1.18 mM KH_2PO_4, 5 mM dextrose, 5 mM pyruvic acid, 1 mM ascorbic acid and 5 mM HEPES (pH 7.4) containing 1% fatty acid free BSA (Tebu-Bio). The fat pads were minced with scissors, added to a 50 mL centrifuge tube and KRH buffer was added to a final volume of 46 mL. 50 mg Collagenase type I and 2 µM nicotinic acid in 4 mL KRH buffer were added and the mixture was digested for 1 h at 37 °C.[7] The resulting mixture was poured through a 200 µm cell strainer (pluriSelect Life Science) that was put onto a new 50 mL centrifuge tube. The filtrate was centrifuged (1 min, 400 G) and subsequently left for 5 min, upon which the adipocytes floated on top of the solution. The infranatant was removed and the adipocyte layer (±5 mL) was washed by addition of 45 mL KRH buffer and repetition of the centrifugation and floatation steps. The remaining 5 mL of adipocytes was dissolved in 45 mL of homogenization buffer (0.25 M sucrose, 1 mM EDTA and 10 mM Tris-HCl (pH 7.4)), put on ice and homogenized by 20 up-and-down strokes of a motor-driven pestle (700 rpm). The homogenate was kept cold and centrifuged (30 min, 15 000 G, 4°C). The upper fat layer (45 mL) was transferred to a new 50 mL centrifuge tube, homogenization buffer (5 mL) was added and the homogenization steps were repeated twice, yielding three supernatant fractions in total. The supernatant fractions were combined and centrifuged (30 min, 15 000 G, 4 °C) to pellet the membranes. The supernatant was removed and

the pellet was redissolved in assay buffer (50 mM Tris-HCl, pH 7.4). Adipocyte membranes from mice typically contain about 0.5-2 pmol of adenosine A_1 receptor per mg of membrane protein.[6]

Radioligand displacement assays

Single point radioligand displacement experiments were performed using 1 μM of competing ligand in the presence of 1.6 nM [^{3}H]DPCPX for CHOhA_1AR, 5.5 nM [^{3}H]ZM241385 for HEK293hA_{2A}AR, 1.5 nM [^{3}H]PSB-603 for CHO-spap-h$_{A2B}$AR, and 10 nM [^{3}H]PSB11 for CHOhA_3AR. Full curve radioligand displacement experiments on the CHOhA_1AR were performed using a concentration range of competing ligand, ranging from 10^{-11} to 10^{-6} M. Nonspecific binding was determined in the presence of 100 μM CPA for CHOhA_1AR, 100 μM NECA for HEK293hA_{2A}AR and CHOhA_3AR, and 10 μM ZM241385 for CHO-spap-hA_{2B}AR. Competing ligand (50 μL) and radioligand (50 μL) were co-incubated with membrane aliquots containing the respective receptor. Membrane aliquots containing 5 μg (CHOhA_1AR) total protein were incubated in a total volume of 100 μL assay buffer (50 mM Tris-HCl, pH 7.4) at 25 °C for 1 h. Membrane aliquots containing 30 μg (HEK293hA_{2A}AR) total protein were incubated in a total volume of 100 μL assay buffer (50 mM Tris-HCl, pH 7.4) at 25 °C for 1 h. Membrane aliquots containing 30 μg (CHO-spap-hA_{2B}R) total protein were incubated in a total volume of 100 μL assay buffer (0.1% CHAPS in 50 mM Tris-HCl, pH 7.4) at 25 °C for 2 h. Membrane aliquots containing 15 μg (CHOhA_3AR) total protein were incubated in a total volume of 100 μL assay buffer (50 mM Tris-HCl, pH 8.0, supplemented with 10 mM MgCl2, 1mM EDTA and 0.01% (w/v) CHAPS) at 25 °C for 2 h. In case of the full curve displacement experiments, CHOhA_1AR membranes and competing ligand were pre-incubated for either 0 or 4 h at 25 °C, prior to addition of radioligand and subsequent co-incubation (as described above). Incubations were terminated by rapid vacuum filtration to separate the bound and free radioligand through prewetted 96-well GF/B filter plates using a PerkinElmer Filtermate-harvester (Perkin Elmer, Groningen, the Netherlands). Filters were subsequently washed 12 times with ice-cold wash buffer: 50 mM Tris-HCl, pH 7.4 for CHOhA_1AR and HEK293hA_{2A}AR; 0.1% BSA in 50 mM Tris-HCl, pH 7.4 for CHO-spap-hA_{2B}AR; and 50 mM Tris-HCl supplemented with 10 mM MgCl2, 1mM EDTA, pH 8.0 for CHOhA_3AR. The plates were dried at 55 °C after which MicroscintTM-20 cocktail was added (Perkin Elmer, Groningen, The Netherlands). After 3 h the filter-bound radioactivity was determined by scintillation spectrometry using a 2450 MicroBeta Microplate Counter (Perkin Elmer, Groningen, The Netherlands).

Wash-out assay

Wash-out assays were performed as previously described, using 100 μg of protein in 100 μL cell membrane suspension.[8]

Functional [^{35}S]GTPγS binding assay

[^{35}S]GTP γS binding assays were performed as previously described, using CHO cells that were stably transfected with the A_1AR.[8]

Data Analysis

All experimental data were analyzed using the non-linear regression curve fitting program GraphPad Prism 7.0 (GraphPad Software Inc., San Diego, CA). IC_{50} values obtained from competition displacement binding data were converted into K_i values using the Cheng-Prusoff equation.[9] The K_D value of [^{3}H]DPCPX (1.6 nM) at CHOhA_1AR membranes was taken from Kourounakis *et al.*[10] The K_D

value (1.0 nM) of [^{3}H]ZM241385 at $hA_{2A}AR$ membranes, the K_D value (1.7 nM) of [^{3}H]PSB603 at CHO-spap-$hA_{2B}AR$ membranes, and the K_D value (17.3 nM) of [^{3}H]PSB11 at $CHOhA_3AR$ membranes were taken from in-house determinations.

SDS-PAGE experiments of LUF7909 in membrane fractions

18 μL of membrane fractions (1 mg/mL) were pre-incubated with 1 μL competing ligand (final concentration: 1 μM, unless stated otherwise) or DMSO (1%) for 1 h (rt, 650 rpm). 1 μl of LUF7909 was added (final concentration: 100 nM, unless stated otherwise) and the membranes were incubated for 1 h (rt, 650 rpm). 1 μL PNGase (10u) or MilliQ water was added and the membranes were incubated for 1 h (rt, 650 rpm). Click mix was prepared freshly by mixing 50 μL 100 mM $CuSO_4$, 30 μL 1 M sodium ascorbate (NaAsc), 10 μL 100 mM tris(3-hydroxypropyltriazolylmethyl)amine (THPTA) and 10 μL 100 μM AF647-N_3. 2.3 μL of click mix was added and the membranes were shaken for 1 h (rt, 650 rpm). 7.8 μL of 4 x Laemmli Sample Buffer (Bio-Rad) containing β-mercaptoethanol was added and the membranes were shaken for 2 h (rt, 650 rpm). The samples were then subjected to 12.5% SDS-PAGE (180 V, 100 min). In-gel fluorescence was measured on a Bio-Rad Universal Hood III using Cy3 (605/50 filter) or Cy5 (695/55 filter) settings. Gels were transferred to 0.2 μm PVDF blots using a Trans-Blot Turbo Transfer System (Bio-Rad)(2.5 A, 7 min) or stained with Coomassie Brilliant Blue. Gel images were analyzed with Image Lab software (Bio-Rad).

SDS-PAGE experiments of LUF7909 in live $CHOhA_1AR$ and CHO cells

$CHOhA_1AR$ or CHO medium (as described above) containing 1 μM DPCPX or 1% DMSO (control) was added to a 10 cm Ø plate containing the respective cells (~90% confluence). The cells were incubated for 1 h (37 °C, 5% CO_2). The medium was removed, replaced by medium containing 100 nM LUF7909 or 1% DMSO (control) and the cells were incubated for 1 h (37 °C, 5% CO_2). The cells were washed with PBS and membranes were collected (as described above). Membrane pellets were diluted to 1 mg/mL and 20 μL was taken per sample. Click mix was prepared freshly by mixing 50 μL 100 mM $CuSO_4$, 30 μL 1 M NaAsc, 10 μL 100 mM THPTA and 10 μL 100 μM AF647-N_3. 2.2 μL of click mix was added and the membranes were shaken for 1 h (rt, 650 rpm). 7.4 μL of 4 x Laemmli Sample Buffer containing β-mercaptoethanol was added and the membranes were shaken for 2 h (rt, 650 rpm). The samples were then subjected to 12.5% SDS-PAGE (180 V, 100 min) and in-gel fluorescence was measured on a Bio-Rad Universal Hood III. Gels were transferred to 0.2 μm PVDF blots using a Trans-Blot Turbo Transfer System (Bio-Rad)(2.5 A, 7 min) or stained with Coomassie Brilliant Blue. Gel images were analyzed with Image Lab software (Bio-Rad).

Western Blot experiments

Blots were blocked in 5% BSA in TBST (1 h, rt), prior to incubation with primary antibody: rabbitαratA_1AR (Sigma Aldrich, cat # A268) 1:5000 in 1% BSA in TBST (overnight, 4 °C). The blots were washed (3 x TBST) and incubated with secondary antibody: goatαrabbit-HRP (Jackson ImmunoResearch Laboratories, cat # 111-035-003) 1:2000 in 1% BSA in TBST. The blots were washed (2 x TBST, 1 x TBS), incubated with 1 mL of luminol enhancer solution and 1 mL of peroxide solution (Pierce™,, ThermoFisher cat # 32106)(3 min, rt, dark) and scanned using chemiluminescence and fluorescence. Blot images were analyzed with Image Lab software (Bio-Rad).

Affinity-based pull-down proteomics[11]

Probe Incubation

A) $CHOhA_1AR$ and CHO membranes

$CHOhA_1AR$ or CHO membrane fractions were resuspended in assay buffer (50 mM Tris-HCl, pH 7.4) and diluted to a concentration of 2 mg/mL. 25 µL of LUF7746 (final concentration: 10 µM), 10% SDS or 1% DMSO in assay buffer was added to 200 µL of membranes and the membranes were incubated for 1 h (rt, 650 rpm). 25 µL of LUF7909 (final concentration: 1 µM) or 1% DMSO in assay buffer was added and the membranes were incubated for 2 h (rt, 650 rpm).

B) Live $CHOhA_1AR$ cells

$CHOhA_1AR$ medium (as described above) containing 1 µM LUF7909 or 1% DMSO (control) was added to a 10 cm Ø plate containing $CHOhA_1AR$ cells (~90% confluence). The cells were incubated for 2 h (37 °C, 5% CO_2). The medium was removed, the cells were washed with PBS and membranes were collected (as described above). Membrane pellets were diluted to 2 mg/mL, 225 µL was taken per sample and 25 µL of 1% DMSO in assay buffer (50 mM Tris-HCl, pH 7.4) was added.

Click reaction, precipitation, reduction and alkylation

Click mix was prepared freshly by mixing 350 µL 100 mM $CuSO_4$, 210 µL 1 M NaAsc, 70 µL 100 mM THPTA and 70 µL 1 mM Biotin-PEG3-Azide. 27.5 µL of click mix was added per sample (from A or B) and the samples were incubated for 1 h (rt, 650 rpm). 92.5 µL 10% SDS (final SDS concentration: 2.5%) was added and the proteins were denatured for 1 h (rt, 650 rpm). Proteins were precipitated based on the method of Wessel and Flügge.[12] In brief, 800 µL MeOH, 400 µL $CHCl_3$ and 400 µL water were added and the proteins were pelleted by centrifugation (10 min, 1 500 G, rt). The upper (aqueous) layer was removed and 600 µL MeOH was added. The samples were centrifuged a second time (10 min, 1 500 G, rt) and supernatant was removed to yield a more purified protein fraction. The proteins were then resuspended in 500 µL 1% SDS containing 25 mM NH_4HCO_3. Probe sonication (Sonics Vibra-Cell, 3 x 5 s, 30% amplitude) was necessary to fully dissolve the membrane proteins. The proteins were reduced by addition of 10 µL 0.5 M dithiothreitol (DTT) (15 min, 65 °C, 700 rpm), alkylated by addition of 80 µL 0.25 M iodoacetamide (IAA) (30 min, rt, dark) and further reduced by addition of 10 µL 0.5 M DTT (15 min, rt, 700 rpm).

Pull-down

1400 µL of Avidin Agarose beads was divided over two 15 mL centrifuge tubes, washed with PBS (4 mL) and centrifuged (2 min, 2 500 G, rt). The supernatant was removed and the washing steps were repeated twice. The washed beads were resuspended in 2.3 mL PBS. 250 µL of the beads solution was added per protein-containing sample and the mixture was added to a 15 mL centrifuge tube containing 9.1 mL PBS (final SDS concentration: 0.05%). The tubes were incubated overnight while rotating at 4 °C. The next day, beads were pelleted (200 G, 2 min, rt), supernatant was removed and the beads were transferred to a 2 mL Eppendorf tube. The beads were washed subsequently with 1 mL 0.1% SDS in PBS, 1 mL PBS (3 x) and 1 mL on-bead digestion buffer (100 mM Tris-HCl pH 8.0, 100 mM NaCl, 10 mM $CaCl_2$ and 2% (v/v) acetonitrile),[11] samples were centrifuged (2 min, 2 500 G, rt) after each step and supernatant fractions were removed.

Digestion and desalting

The remaining beads were dissolved in 250 µL on-bead digestion buffer. 2 µL chymotrypsin (0.5 µg/µL in 1 mM HCl) (cat # V1062, Promega) was added and the proteins were digested overnight (1000 rpm, 37 °C). The samples were quenched by addition of 12.5 µL of formic acid and beads were removed by

centrifugation with Bio-spin columns (Bio-Rad) (2 min, 600 G, rt). Samples were purified using StageTips, as reported by Rappsilber *et al*. and van Rooden *et al*.[11,13] Briefly, StageTips were pre-conditioned with 50 µL MeOH, 50 µL of 0.5% (v/v) formic acid in H_2O:MeCN 2:8 and 50 µL of 0.5% (v/v) formic acid in H_2O. Peptide samples were then loaded on the StageTips and washed by addition of 100 µL of 0.5% (v/v) formic acid in H_2O and centrifugation (2 min, 600 G, rt). Peptides were eluted in low-binding Eppendorf tubes by addition of 0.5% (v/v) H_2O:MeCN 8:2 to the StageTips and subsequent centrifugation (2 min, 600 G, rt). The solvents were evaporated in an Eppendorf Concentrator Plus (60 °C).

Nano-LC-MS Settings

Desalted peptide samples were reconstituted in 50 µl 97:3:0.1 solution (H2O, MeCN, FA) containing 10 fmol/µl yeast enolase digest. A mix, quality control (QC), sample was made by taking a few microliters from each sample and carefully titrated to prevent overloading the nanoLC system and the automatic gain control (AGC) of the QExactive mass spectrometer.

The desalted peptides solution was separated on an UltiMate 3000 RSLCnano system set in a trap-elute configuration with a nanoEase M/Z Symmetry C18 100Å, 5µm, 180µm x 20 mm (Waters) trap column for peptide loading/retention and nanoEase M/Z HSS C18 T3 100Å, 1.8µm, 75 µm x 250 mm (Waters) analytical column for peptide separation. The column was kept at 40°C in a column oven. Samples were injected on the trap column at a flow rate of 15 µl/min for 2 min with 99%A, 1%B eluent. The 85 min LC method, using mobile phase A (0.1% formic acid (FA) in ULC-MS grade water (Biosolve)) and mobile phase B (0.1% FA in ULC-MS grade acetonitrile (MeCN, Biosolve)) controlled by a flow sensor at 0.3µl/min with average pressure of 400-500 bar (5500-7000 psi), was programmed as gradient with linear increment to 1% B from t0 to t2 min, 5%B at t5 min, 22%B at t55, 40%B at t64, 90%B at t65 to t74 and 1%B at t75 to t85 min. The eluent was introduced by electro-spray ionization (ESI) via the nanoESI source (Thermo) using stainless steel Nano-bore emitters (40 mm, OD 1/32", ES542, Thermo Scientific).

The QExactive HF was operated in positive mode with data dependent acquisition without the use of lock mass, default charge of 2+ and external calibration with LTQ Velos ESI positive ion calibration solution (88323, Pierce, Thermo) every 5 days to less than 2 ppm. The tune file for the survey scan was set to scan range of 350 – 1400 m/z, 60.000 resolution (m/z 200), 1 microscan, automatic gain control (AGC) of 1e6, max injection time of 50 ms, no sheath, aux or sweep gas, spray voltage ranging from 1.7 to 3.0 kV, capillary temp of 250°C and an S-lens value of 80. For the 10 data dependent MS/MS events the loop count was set to 10 and the general settings were resolution to 15,000, AGC target 1e5, max IT time 100 ms, isolation window of 1.6 m/z, no fixed first mass and normalized collision energy (NCE) of 28 eV. For individual peaks the data dependent settings were 5.00e4 for the minimum AGC target yielding an intensity threshold of 5.0e5 that needs to be reached prior of triggering an MS/MS event. No apex trigger was used, unassigned, +1 and charges >+8 were excluded with peptide match mode preferred, isotope exclusion on and dynamic exclusion of 20 sec.

In between experiments, routine wash and control runs were done by injecting 5 µl 97.3.0.1 solution, 5 µl of 10 fmol/µl BSA or enolase digest and 1 µl of 10 fmol/µl angiotensin III (Fluka, Thermo)/oxytocin (Merck) to check the performance of the platform on each component (nano-LC, the mass spectrometer (mass calibration/quality of ion selection and fragmentation) and the search engine).

LC-MS/MS Data processing

MaxQuant (version 1.6.17.0)[14] was used for peptide identification and quantification using a custom made fasta file consisting of the Chinese Hamster proteome from the Uniprot database (UPID: UP000001075, downloaded January 12, 2021), the BETAS background (BSA P02769, yeast enolase P00924, trypsin pig P00761, avidin P02701 and streptavidin P22629) and the adenosine A_1 receptor plus its isoform (P30542-1 and P30542-2). The following changes and additions were made to the standard settings of MaxQuant: The digestion enzyme was set to Chymotrypsin+ with 2 max. missed cleavages. Label-free quantification was chosen with an LFQ min. ratio count of 2. "Match between runs" was enabled. The minimum amount of peptides for protein identification was set to 3. The peptide length was set to be between 7 and 25 with a max. peptide mass of 4600 Da. Oxidation (M) was set as possible peptide modifications and Carbamidomethyl (C) was set as fixed peptide modification. Contaminants were included. An FDR of 0.01 was used for PSM FDR, Protein FDR and Site decoy FDR. Six technical replicates of four different conditions were analysed in the same MaxQuant analysis.

The "peptides.txt" and "proteingroups.txt" files were used for further analysis. Proteins labelled as contaminant were removed from the output files. The LFQ intensities of major proteins were further analysed in GraphPad Prism 8.1.1. for Windows (GraphPad Software Inc., San Diego, CA). Values of six technical replicates were used per condition. The $\log_2$(ratio) and p-values were determined by standard Volcano Plot settings, using multiple t-tests to calculate the p-values. Log_2(ratio) values show the ratio between the probe positive samples (+1 μM LUF7909) and the control samples (DMSO, or pre-incubation with 1%SDS or 10 μM LUF7746).

Click microscopy experiments using LUF7909 in CHOhA_1AR and CHO cells[15]

CHOhA_1AR and CHO cells were seeded in 96-wells plates and grown overnight in their respective medium (as described above). The next day, medium was replaced by medium containing 1 μM of DPCPX, 1 μM of FSCPX or 1% DMSO (control) and the cells were incubated for 1 h (37 °C, 5% CO_2). The medium was then replaced by medium containing 100 nM LUF7909 or 1% DMSO (control) and the cells were incubated for 1 h (37 °C, 5% CO_2). Excess probe was washed away with PBS and the cells were fixed by incubation with a solution of 4% PFA in 10% formalin for 10 min. The remaining fixative was washed away with PBS and 20 mM glycine in PBS and subsequently the cells were permeabilized by a 10 min incubation with 0.1% saponin in PBS. Remaining saponin was washed away with PBS and the fixed cells were stored at 4 °C until further steps were taken. Click mix was prepared freshly by mixing 100 μL 100 mM $CuSO_4$, 100 μL 1 M NaAsc, 100 μL 100 mM THPTA, 9.66 mL HEPES buffer (pH = 7.4) and 40 μL 1 mM Azide-Fluor-545. 100 μL of click mix was added per well and the fixed cells were incubated for 1 h (rt, dark). Remaining click mix was washed away with PBS and by incubation for 30 min with 1% BSA in PBS. The fixed cells were stored in PBS containing 300 nM DAPI until imaging by confocal microscopy.

Image acquisition

Microscopy was performed on a Nikon Eclipse Ti2 C2+confocal microscope (Nikon, Amsterdam, The Netherlands) and this system included an automated xy-stage, an integrated Perfect Focus System (PFS) and 408 and 561 lasers. The system was controlled by Nikon's NIS software. All images were acquired using a 20x objective with 0.7 NA, at a resolution of 1024×1024 pixels for the main figure and 512x512 pixels for the SI figure. The acquisition of 9 fields of view per well was done automatically using the NIS Jobs functionality. Representative images are shown in the figure and created by using OMERO.[16]

Quantification of the Tamra-N_3 signal at single cell level

CellProfiler (version 2.2.0) was used to create a binary image of the Dapi channel and to propagate the cytoplasmic area based on the Dapi binary. An overlay of the binary cytoplasm/Tamra-N_3 channel was generated to quantify per segmented pixel the Tamra-N_3 intensity. The sum of these intensities in the cytoplasm mask is referred to as the integrated Tamra-N_3 intensity in the cytoplasm. Segmentation results were further processed using Excel while GraphPadPrism 9 was used for data visualization and statistics.

NMR Spectra

^{1}H NMR ($(CD_3)_2SO$, 500 MHz)

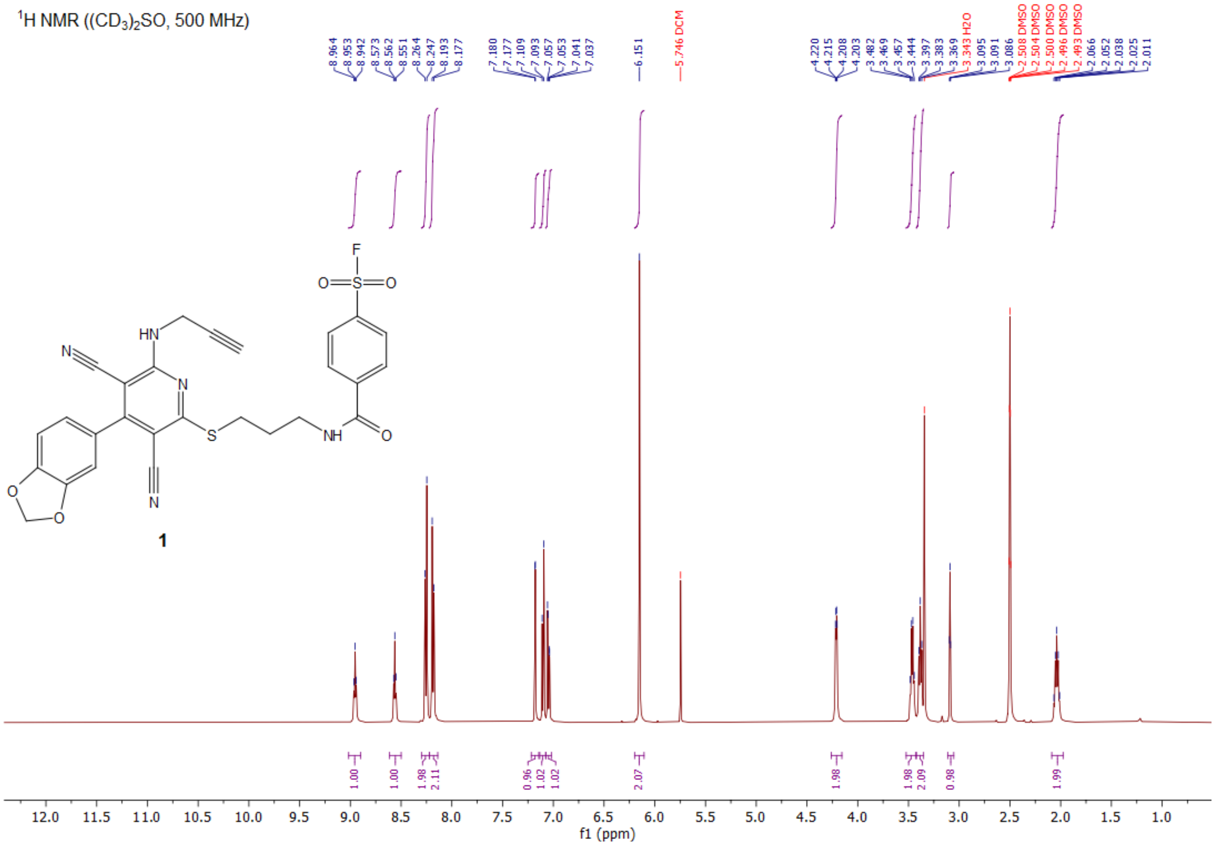

^{13}C NMR ($(CD_3)_2SO$, 126 MHz)

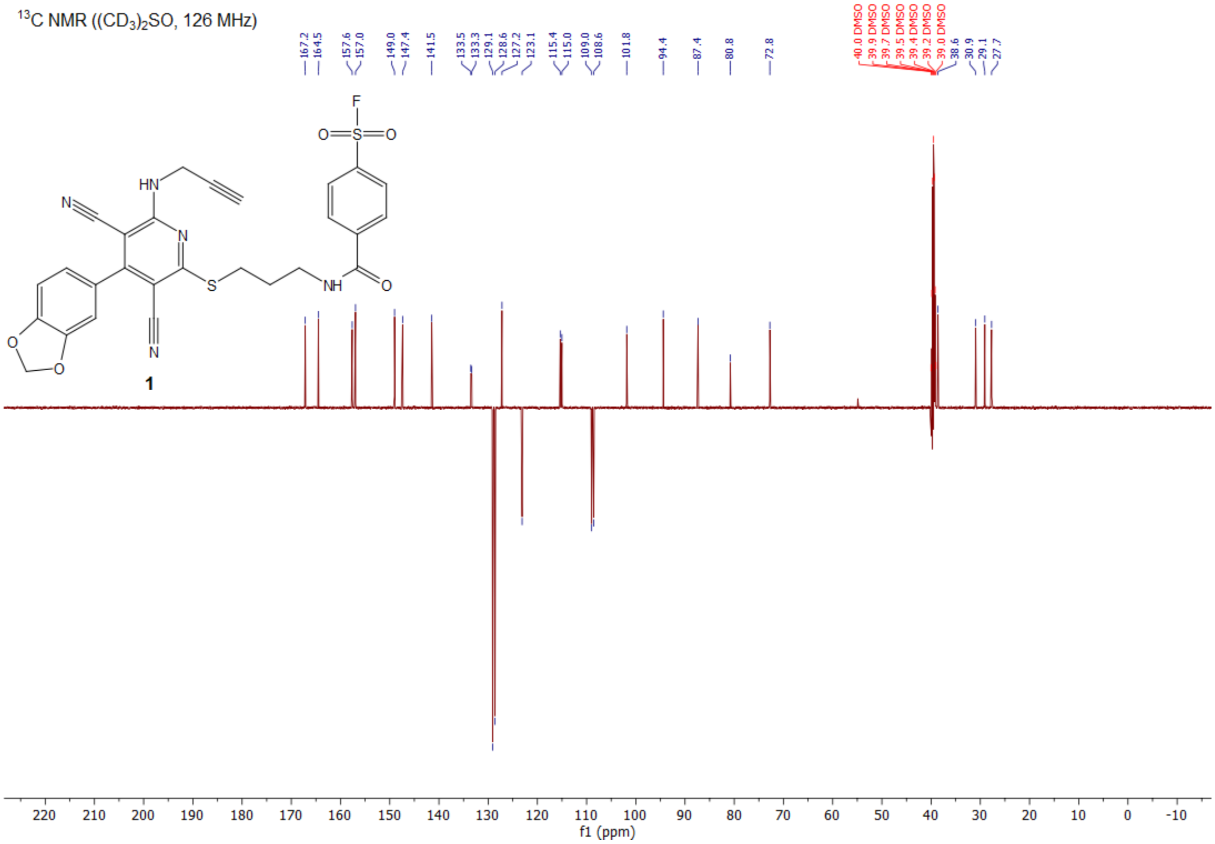

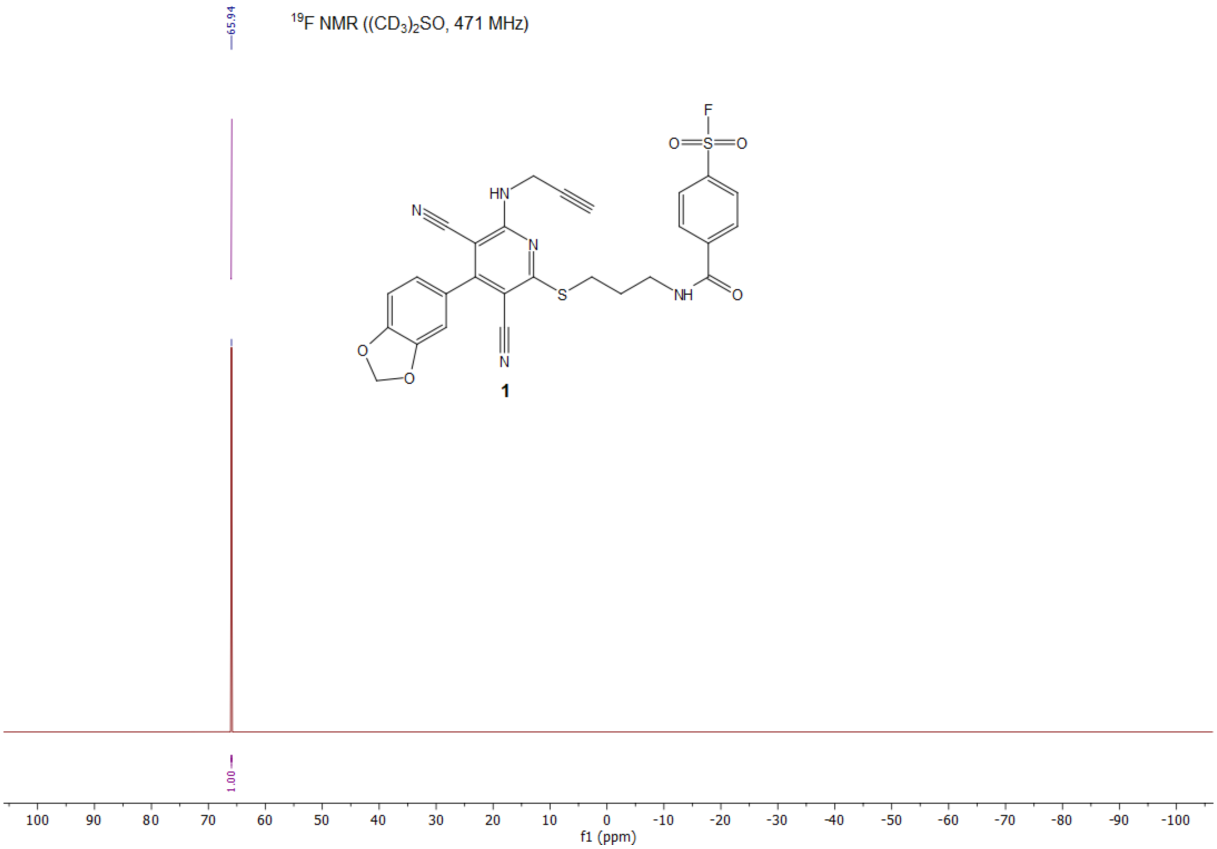

—65.94
^{19}F NMR ((CD$_3$)$_2$SO, 471 MHz)
F
O
S
O
HN
N
N
S
NH
O
O
O
N
1
1.00
100 90 80 70 60 50 40 30 20 10 0 -10 -20 -30 -40 -50 -60 -70 -80 -90 -100
f1 (ppm)

^{1}H NMR (($CD_3)_2SO$, 500 MHz)

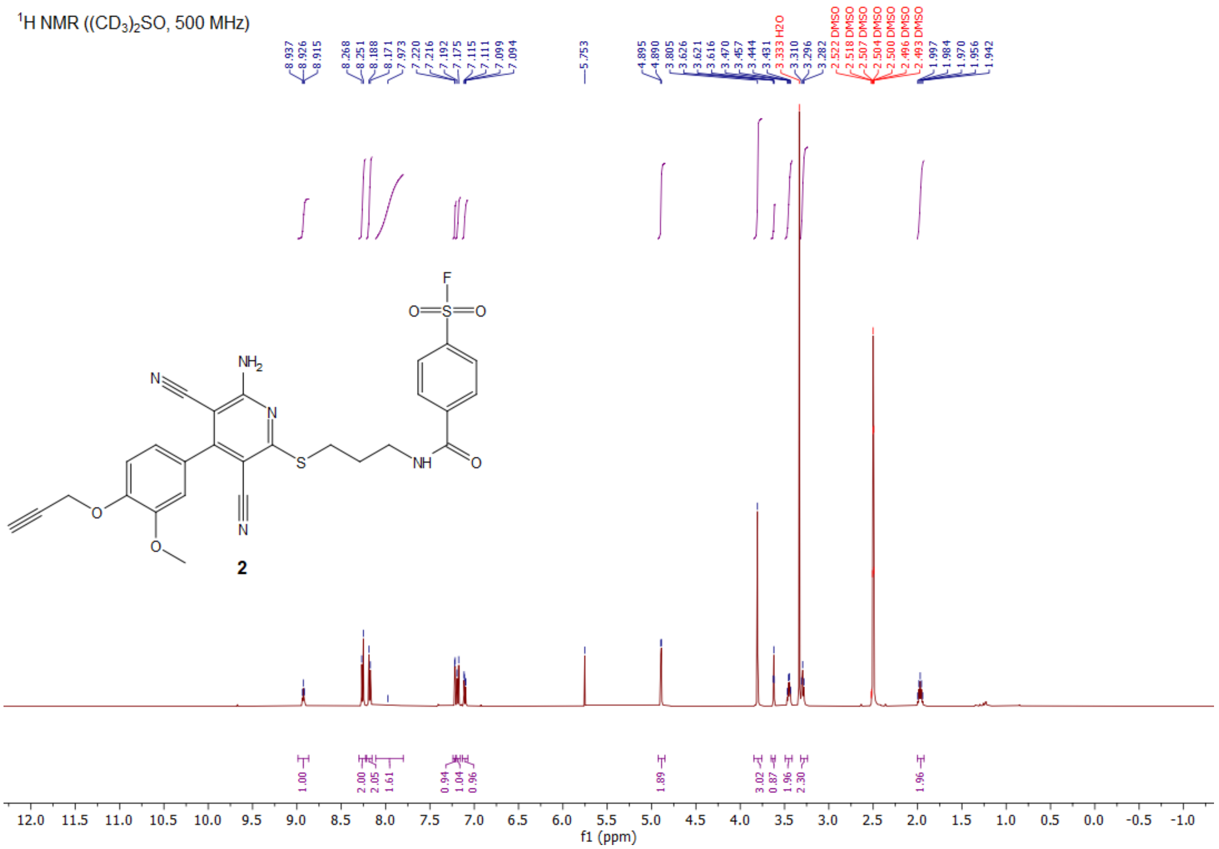

^{13}C NMR (($CD_3)_2SO$, 126 MHz)

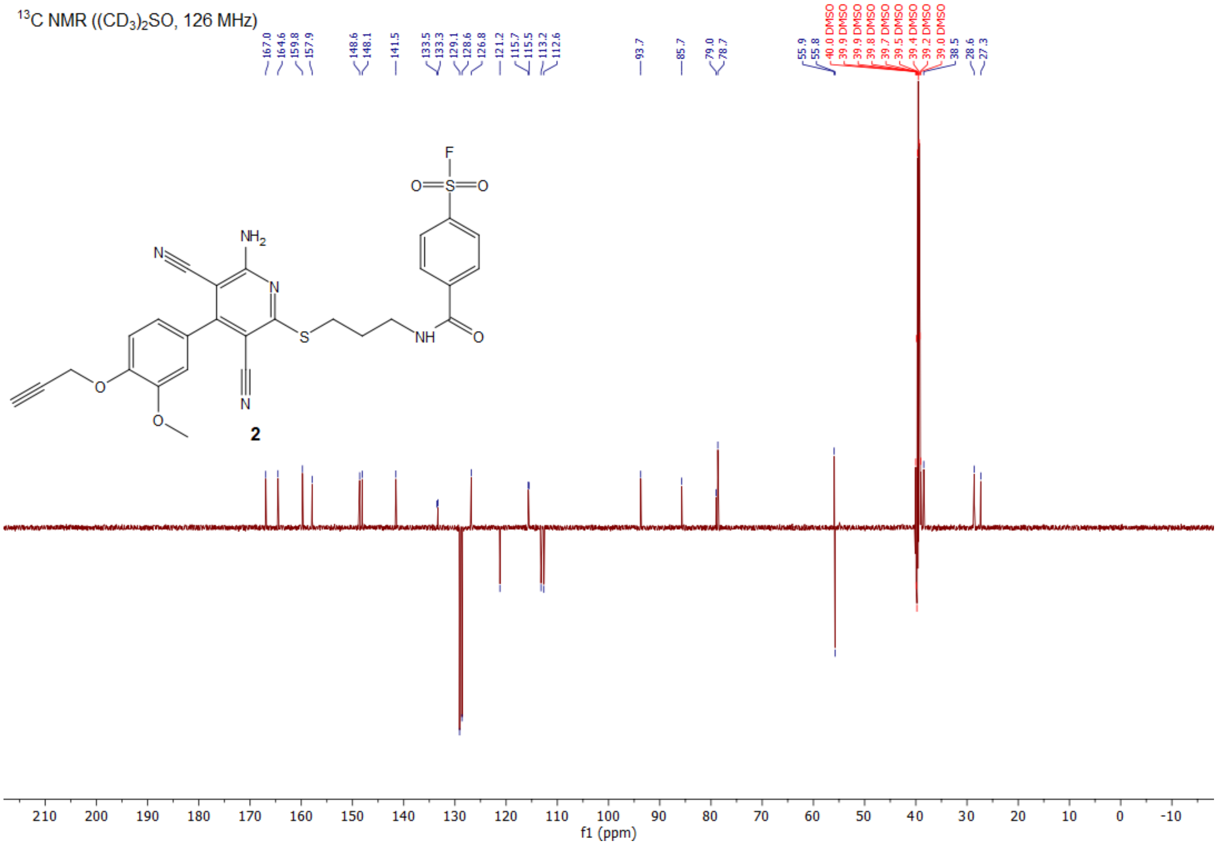

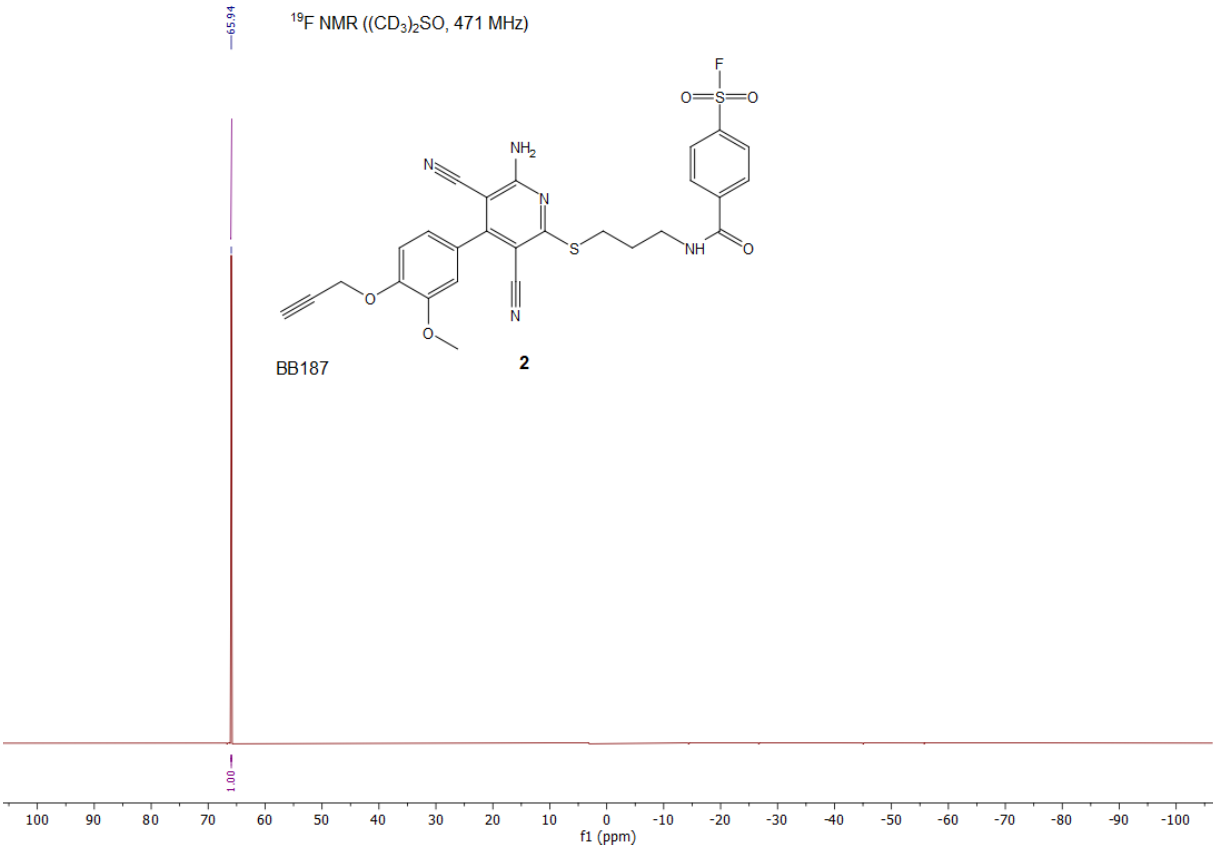

—65.94
^{19}F NMR ((CD3)2SO, 471 MHz)
F
O=S=O
NH2
N
N
S
NH
O
O
O
N
BB187
2
1.00
100 90 80 70 60 50 40 30 20 10 0 -10 -20 -30 -40 -50 -60 -70 -80 -90 -100
f1 (ppm)

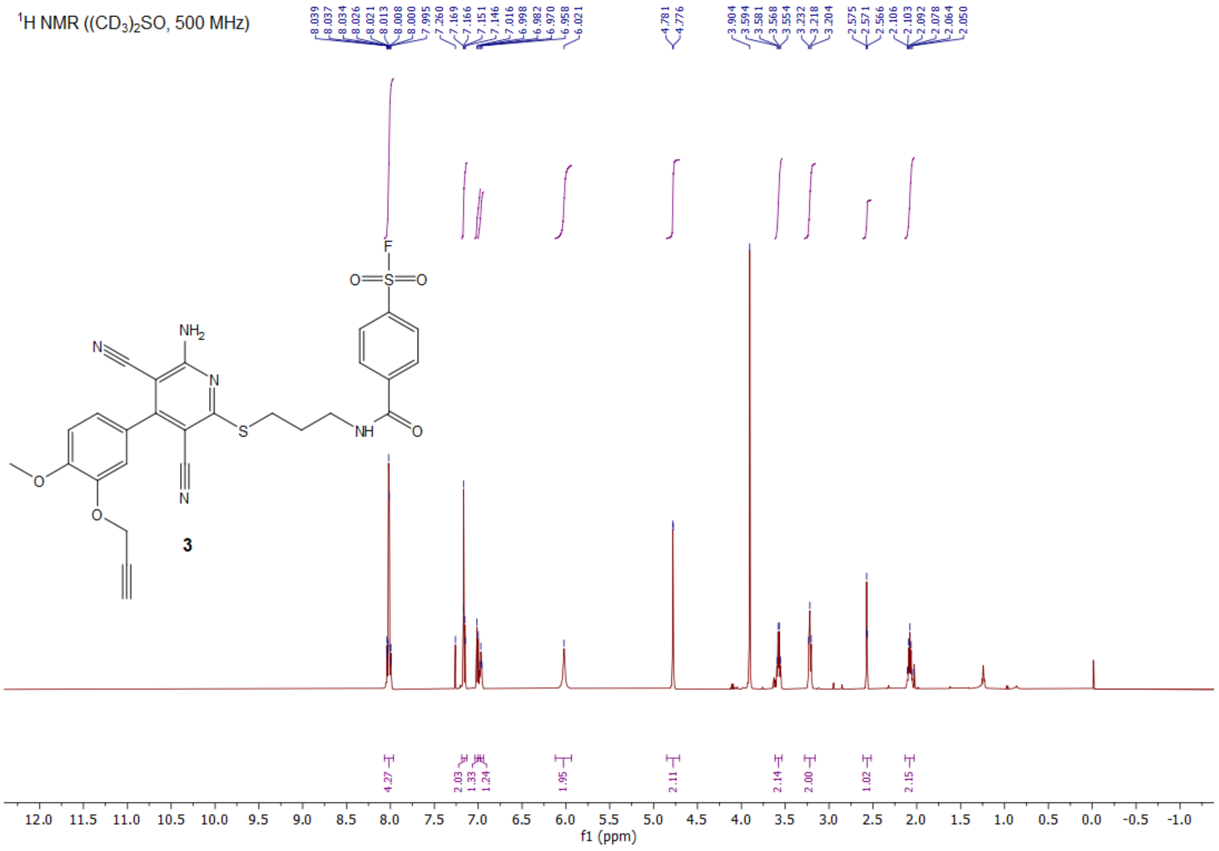

^{1}H NMR ((CD$_3$)$_2$SO, 500 MHz)
3
f1 (ppm)

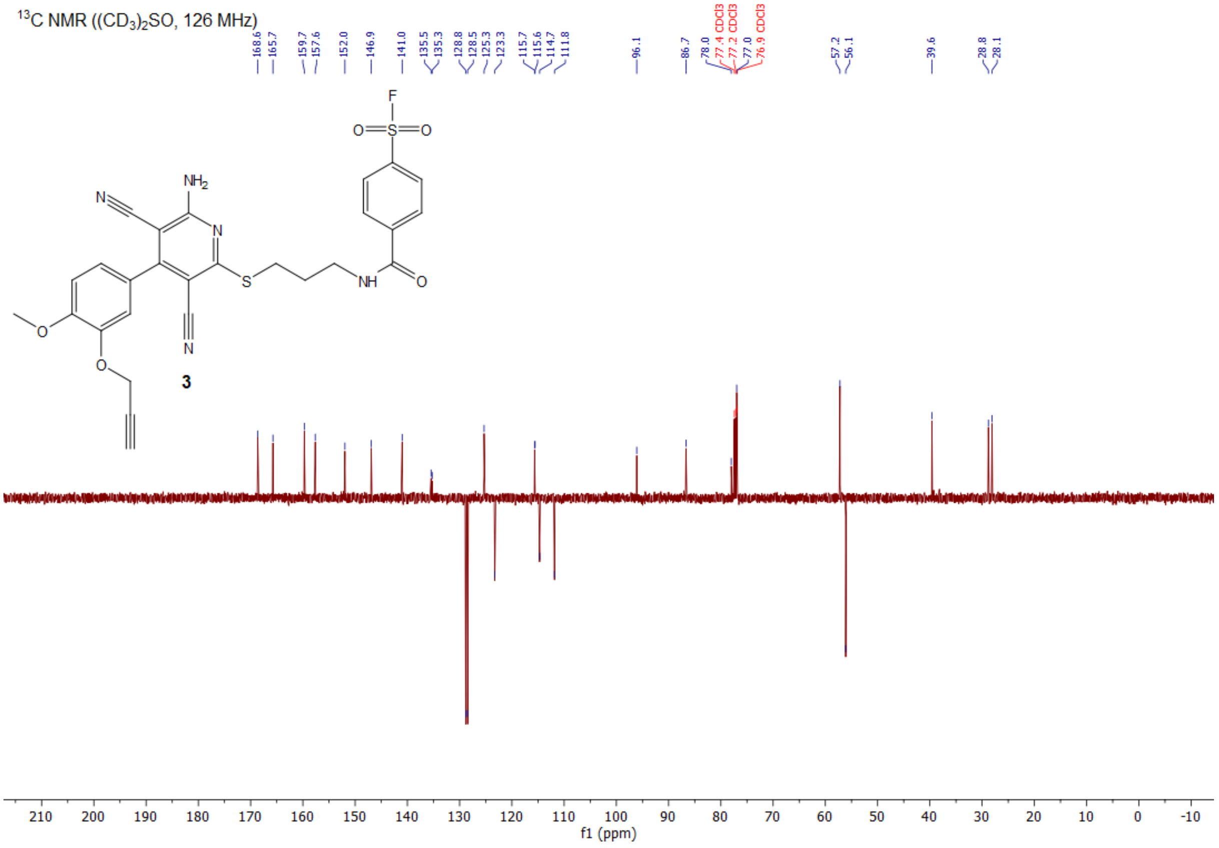

^{13}C NMR ((CD$_3$)$_2$SO, 126 MHz)
3
f1 (ppm)

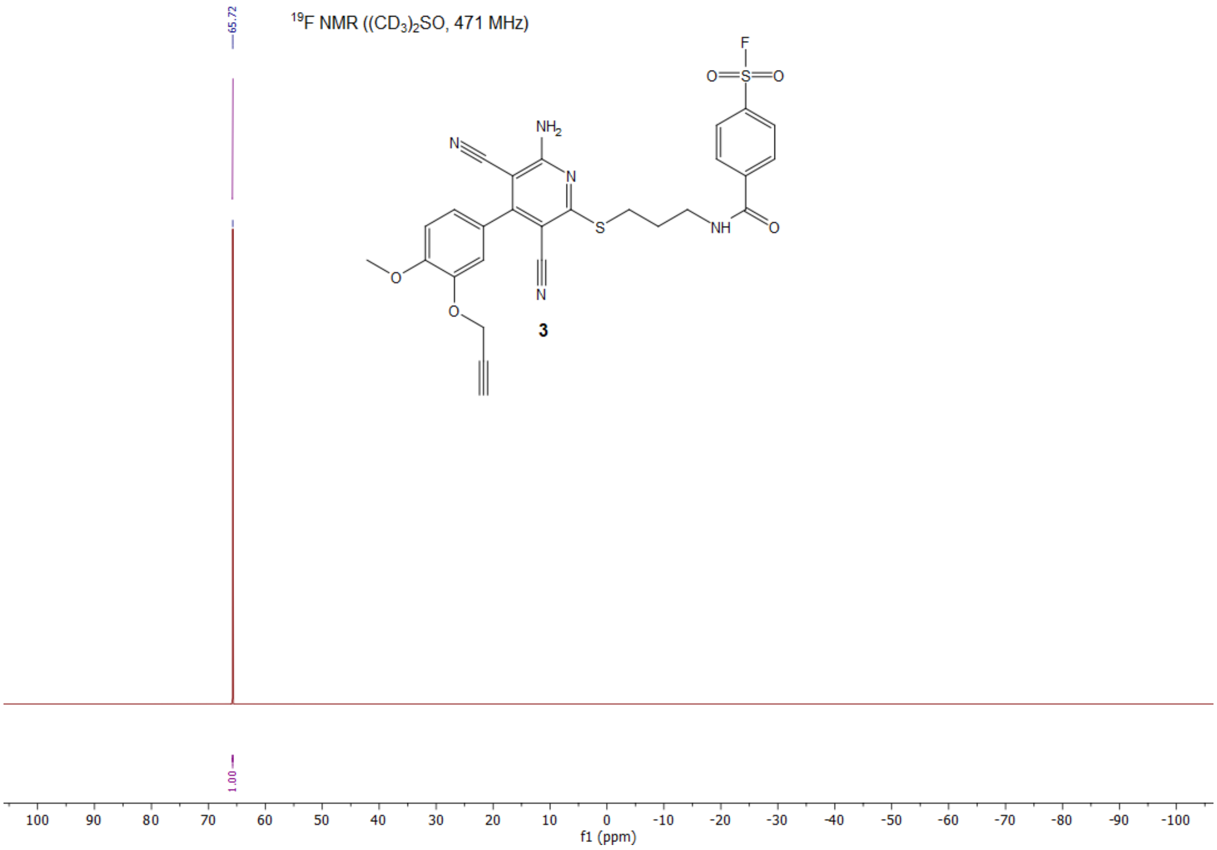

—65.72
19F NMR ((CD3)2SO, 471 MHz)
3
1.00
100 90 80 70 60 50 40 30 20 10 0 -10 -20 -30 -40 -50 -60 -70 -80 -90 -100
f1 (ppm)

^{1}H NMR ((CD$_3$)$_2$SO, 500 MHz, 20 °C)

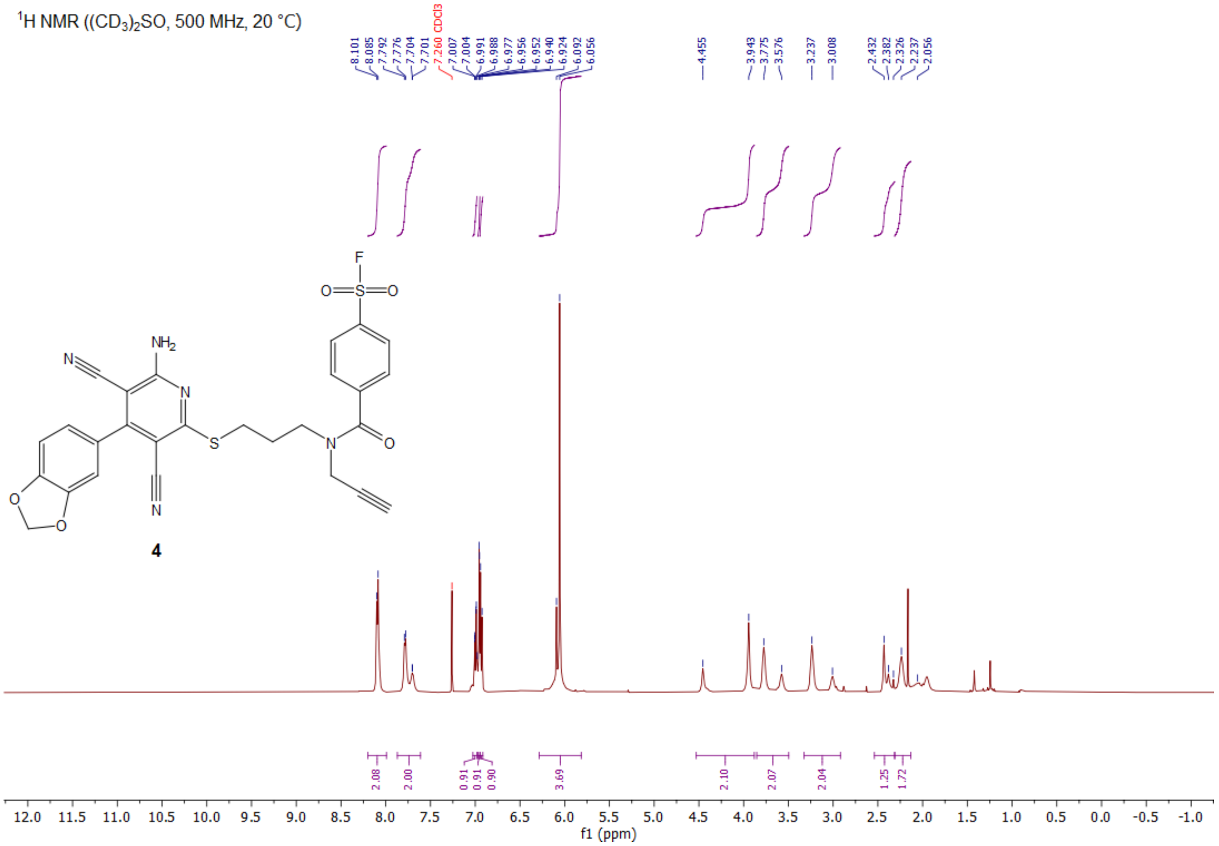

^{1}H NMR ((CD$_3$)$_2$SO, 500 MHz, 59 °C)

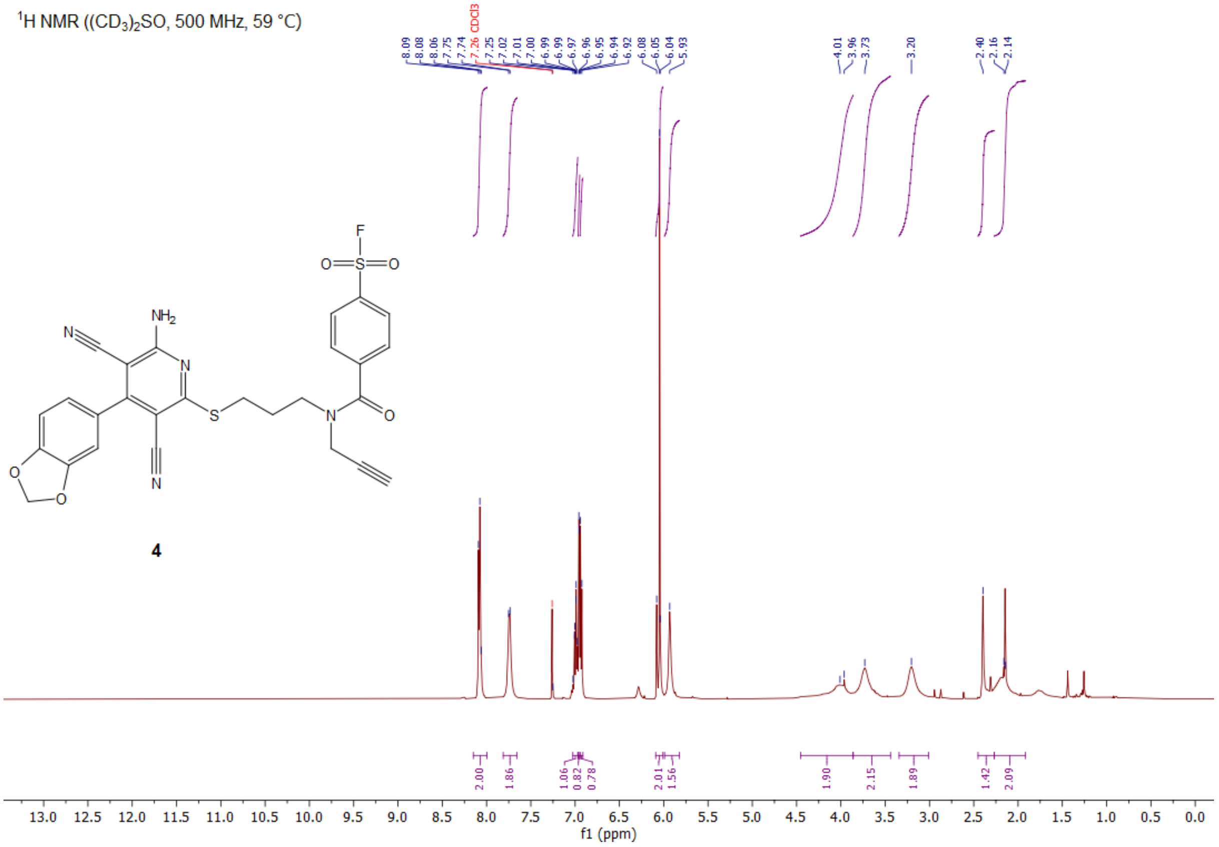

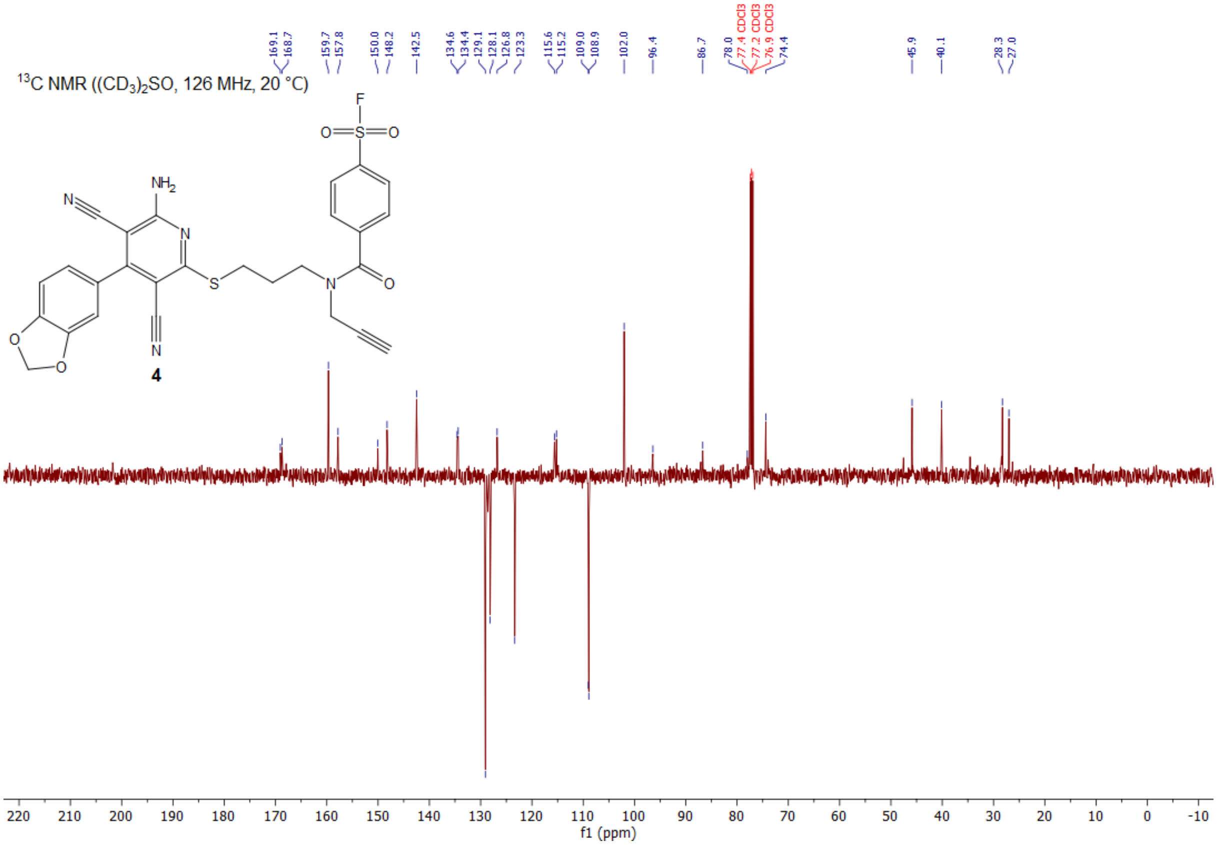

^{13}C NMR ((CD$_3$)$_2$SO, 126 MHz, 20 °C)
169.1
168.7
159.7
157.8
150.0
148.2
142.5
134.6
134.4
129.1
128.1
126.8
123.3
115.6
115.2
109.0
108.9
102.0
96.4
86.7
78.0
77.4 CDCl3
77.2 CDCl3
76.9 CDCl3
74.4
45.9
40.1
28.3
27.0
F
O
S
O
NH2
N
N
S
N
O
O
O
N
4
f1 (ppm)

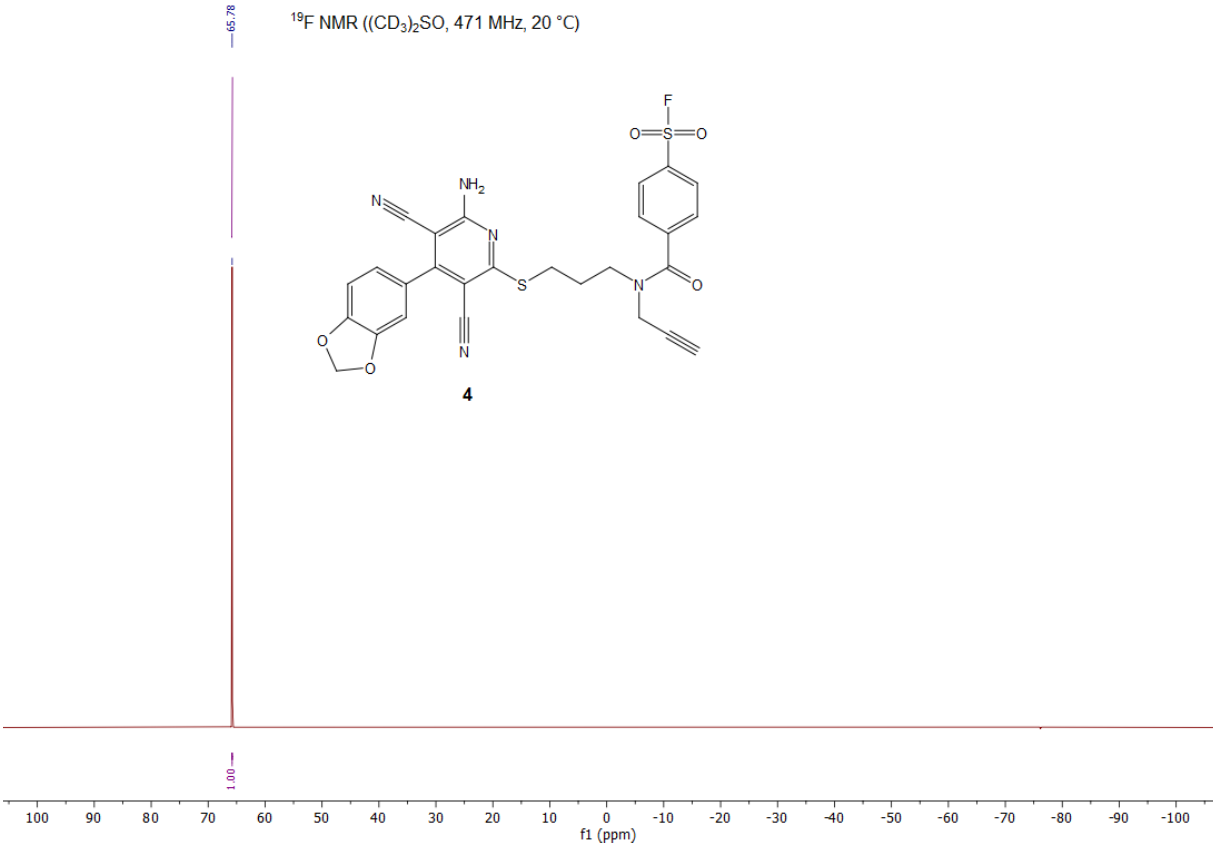

^{19}F NMR ((CD$_3$)$_2$SO, 471 MHz, 20 °C)
65.78
1.00
F
O
S
O
NH2
N
N
S
N
O
O
O
N
4
f1 (ppm)

^{1}H NMR ($CDCl_3$, 400 MHz)

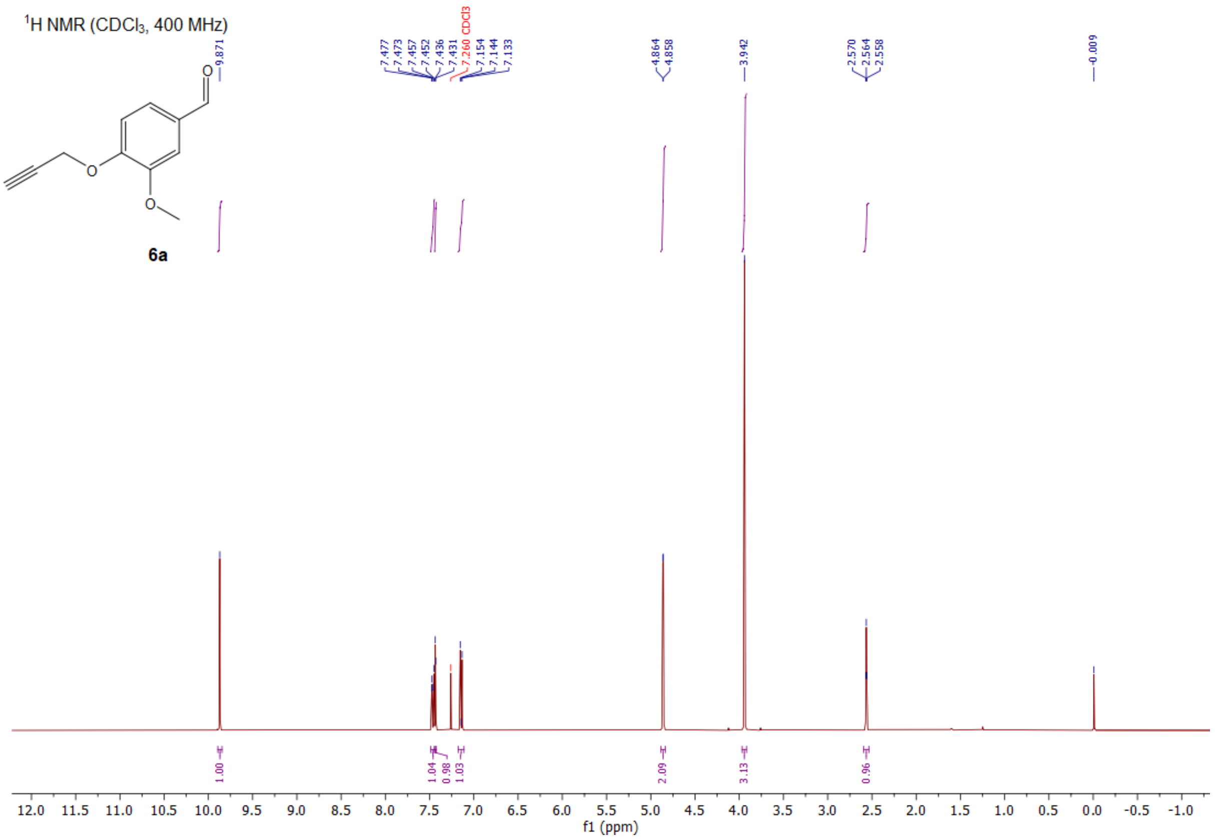

6a

^{1}H NMR ($CDCl_3$, 400 MHz)

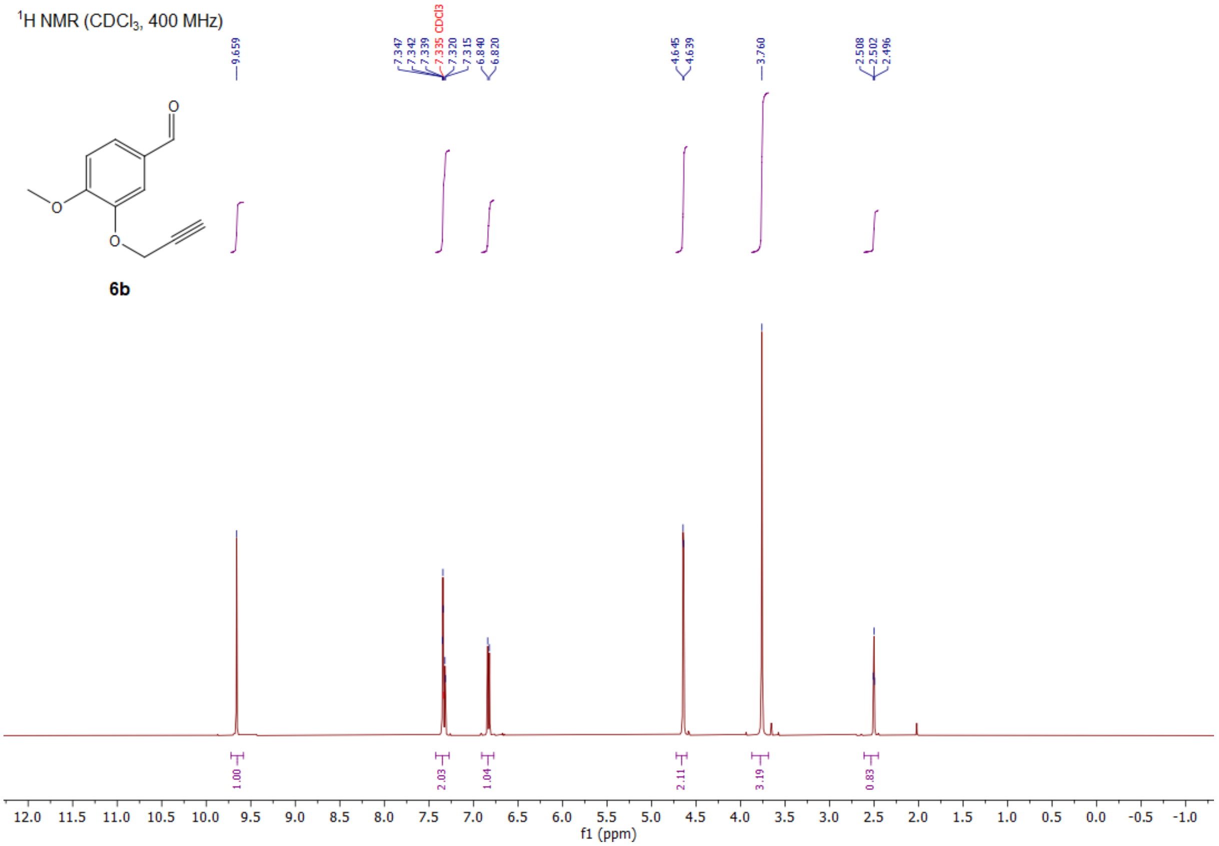

6b

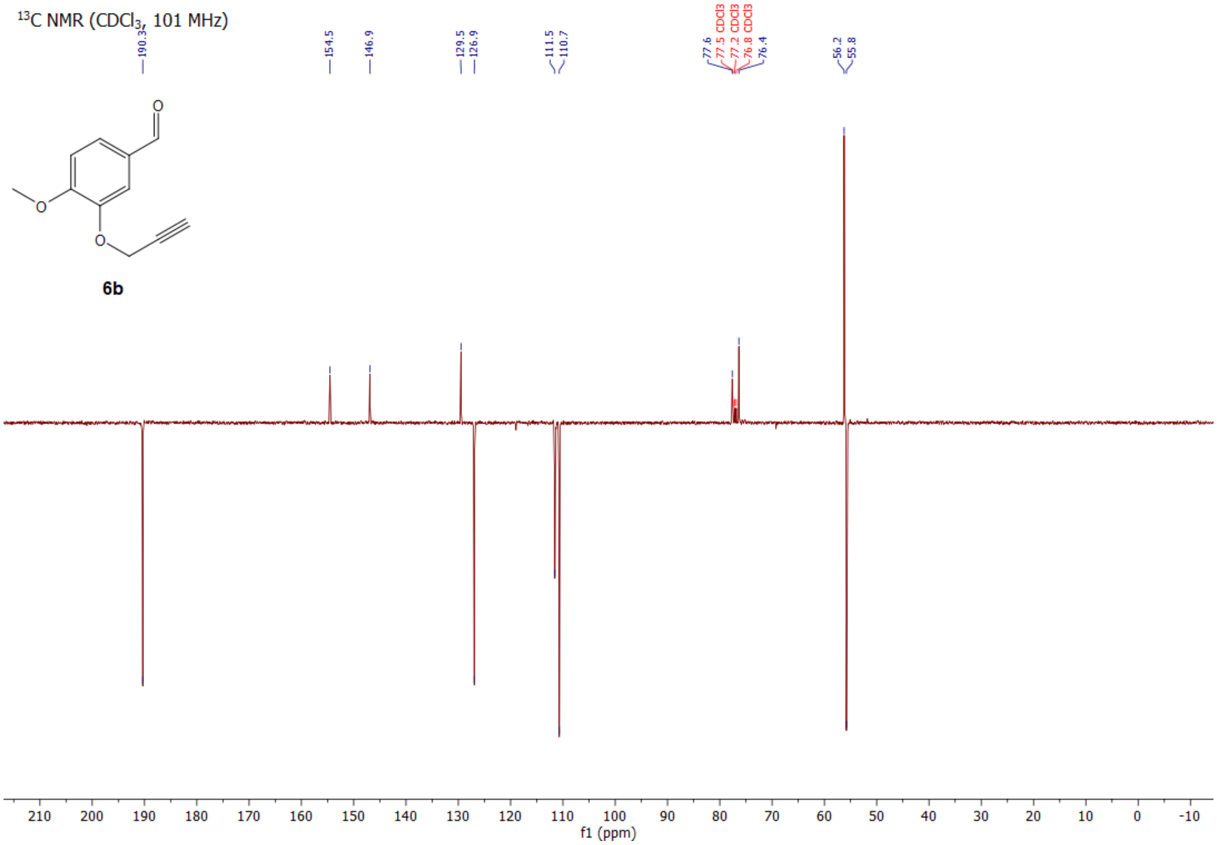

^{13}C NMR ($CDCl_3$, 101 MHz)
190.3
154.5
146.9
129.5
126.9
111.5
110.7
77.6
77.5 CDCl3
77.2 CDCl3
76.8 CDCl3
76.4
56.2
55.8
6b
210 200 190 180 170 160 150 140 130 120 110 100 90 80 70 60 50 40 30 20 10 0 -10
f1 (ppm)

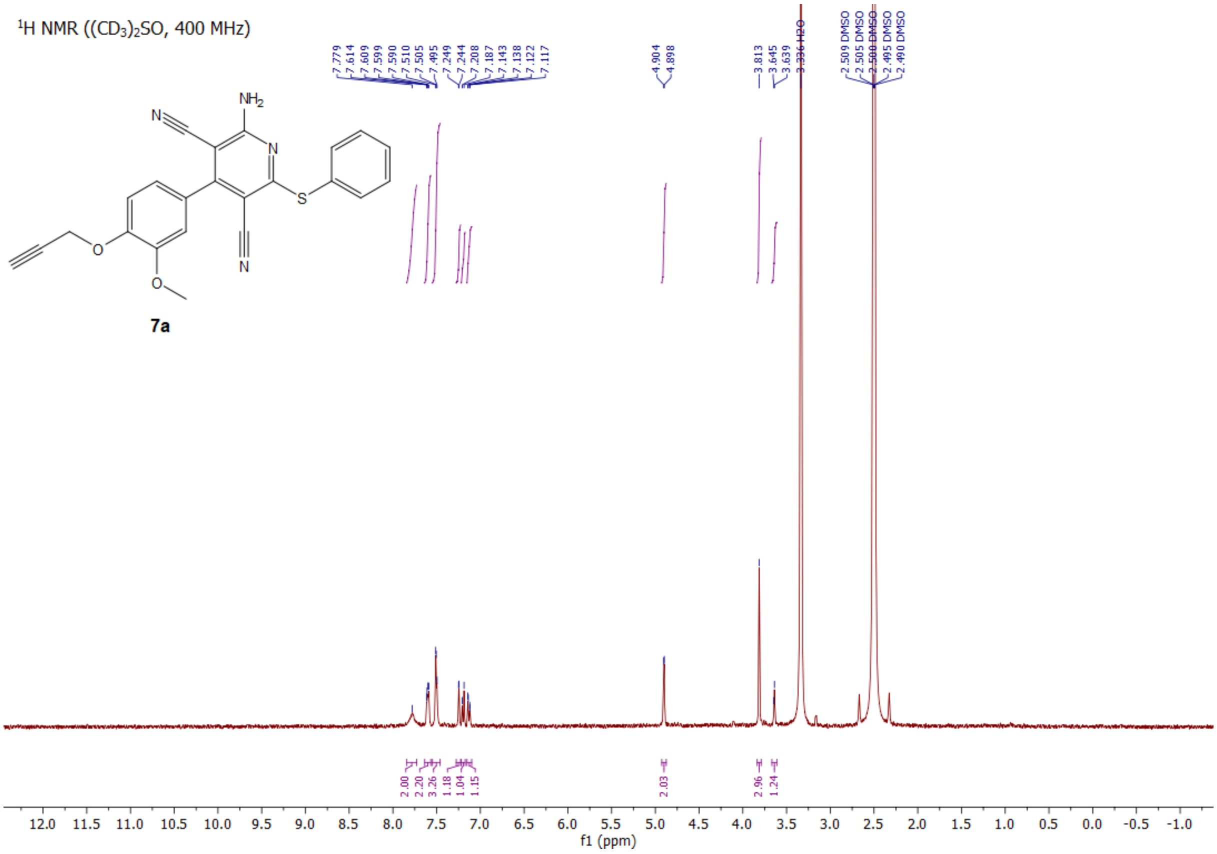

^{1}H NMR ($(CD_3)_2SO$, 400 MHz)
7.779
7.614
7.609
7.599
7.590
7.510
7.505
7.495
7.249
7.244
7.208
7.187
7.143
7.138
7.122
7.117
4.904
4.898
3.813
3.645
3.639
3.336 H2O
2.509 DMSO
2.505 DMSO
2.500 DMSO
2.495 DMSO
2.490 DMSO
NH2
N
N
N
S
O
O
7a
2.00
2.20
3.26
1.18
1.04
1.15
2.03
2.96
1.24
12.0 11.5 11.0 10.5 10.0 9.5 9.0 8.5 8.0 7.5 7.0 6.5 6.0 5.5 5.0 4.5 4.0 3.5 3.0 2.5 2.0 1.5 1.0 0.5 0.0 -0.5 -1.0
f1 (ppm)

^{1}H NMR ($CDCl_3$, 300 MHz)

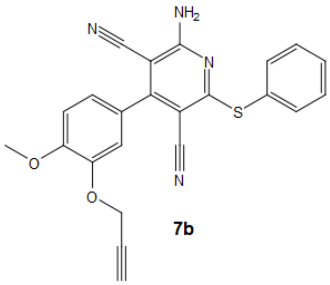

7b

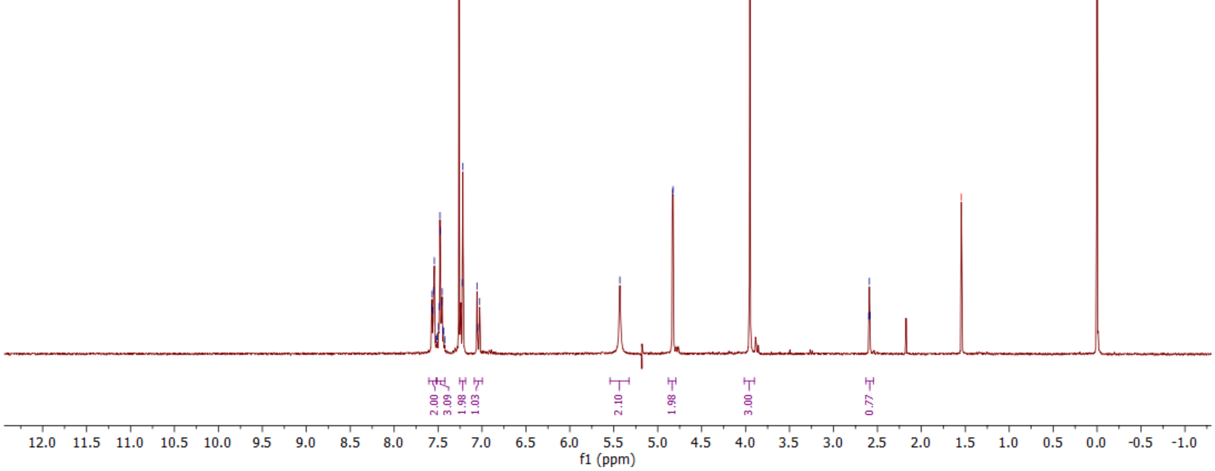

^{1}H NMR ($CDCl_3$, 300 MHz)

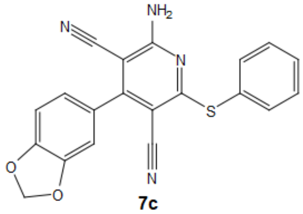

7c

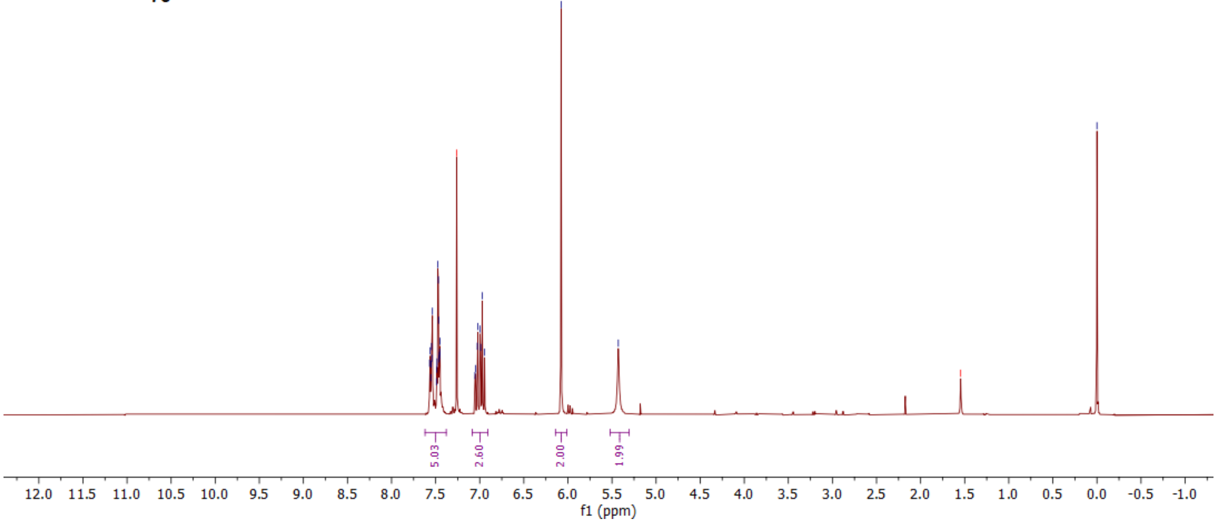

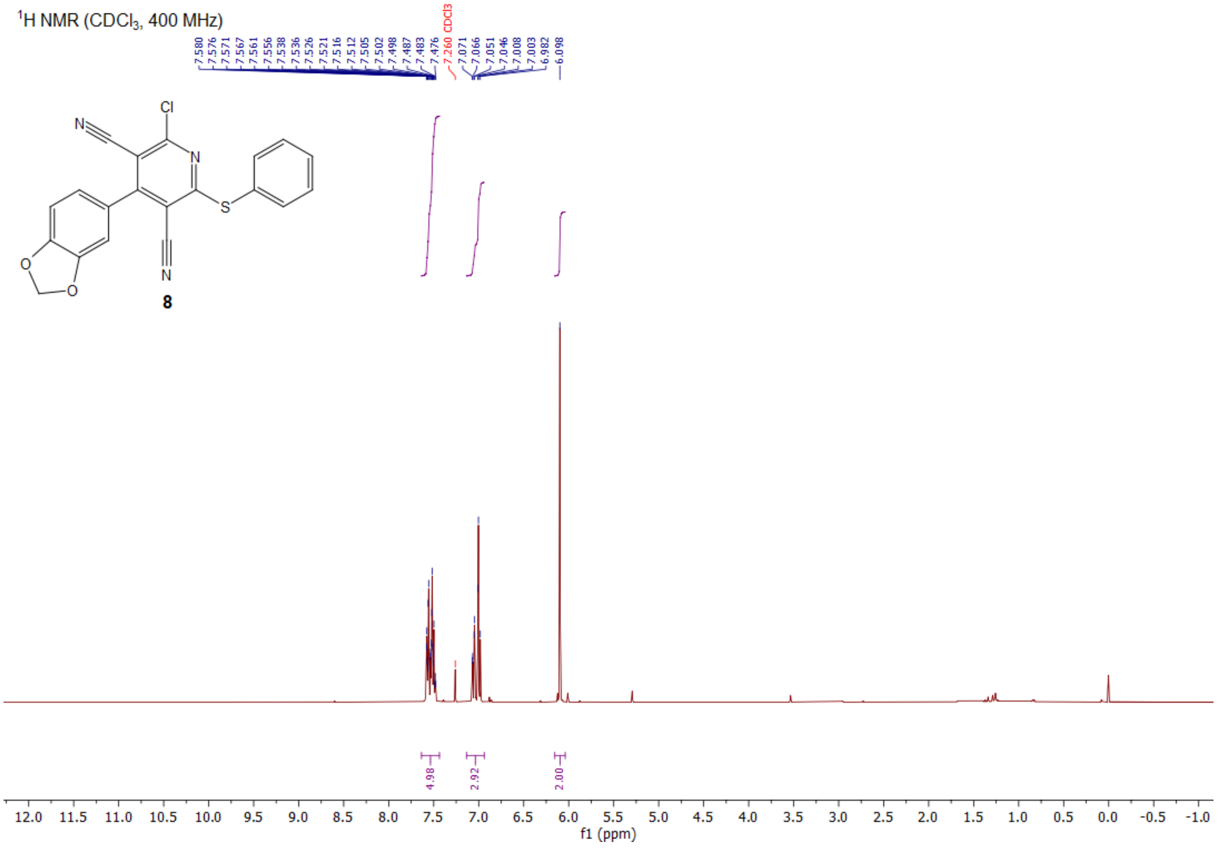

¹H NMR (CDCl₃, 400 MHz)
7.580 7.576 7.571 7.567 7.561 7.556 7.538 7.536 7.526 7.521 7.516 7.512 7.505 7.502 7.498 7.487 7.483 7.476
7.260 CDCl3
7.071 7.066 7.051 7.046 7.008 7.003 6.982
6.098
Cl
N
S
O
O
8
4.98
2.92
2.00
12.0 11.5 11.0 10.5 10.0 9.5 9.0 8.5 8.0 7.5 7.0 6.5 6.0 5.5 5.0 4.5 4.0 3.5 3.0 2.5 2.0 1.5 1.0 0.5 0.0 -0.5 -1.0
f1 (ppm)

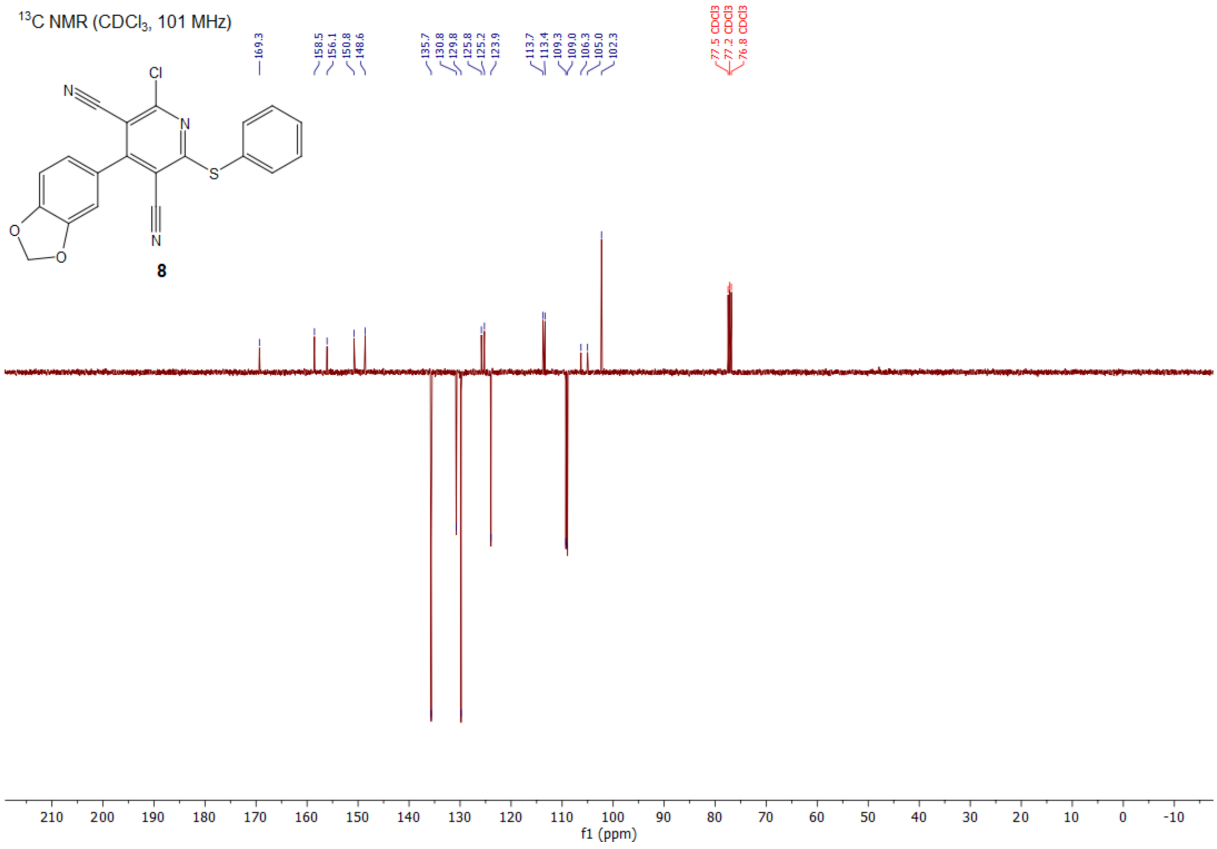

¹³C NMR (CDCl₃, 101 MHz)
169.3
158.5 156.1 150.8 148.6
135.7 130.8 129.8 125.8 125.2 123.9
113.7 113.4 109.3 109.0 106.3 105.0 102.3
77.5 CDCl3
77.2 CDCl3
76.8 CDCl3
Cl
N
S
O
O
8
210 200 190 180 170 160 150 140 130 120 110 100 90 80 70 60 50 40 30 20 10 0 -10
f1 (ppm)

^{1}H NMR ($CDCl_3$, 500 MHz)

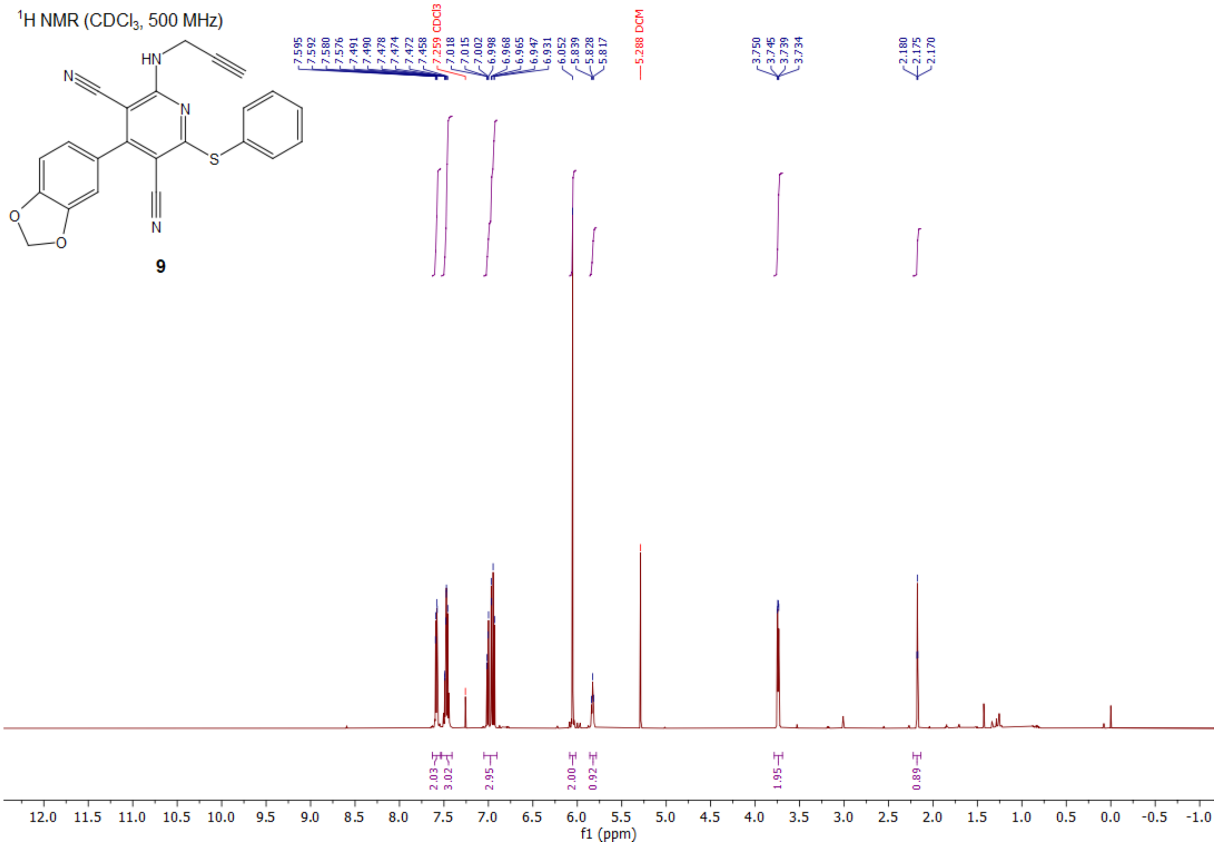

^{13}C NMR ($CDCl_3$, 126 MHz)

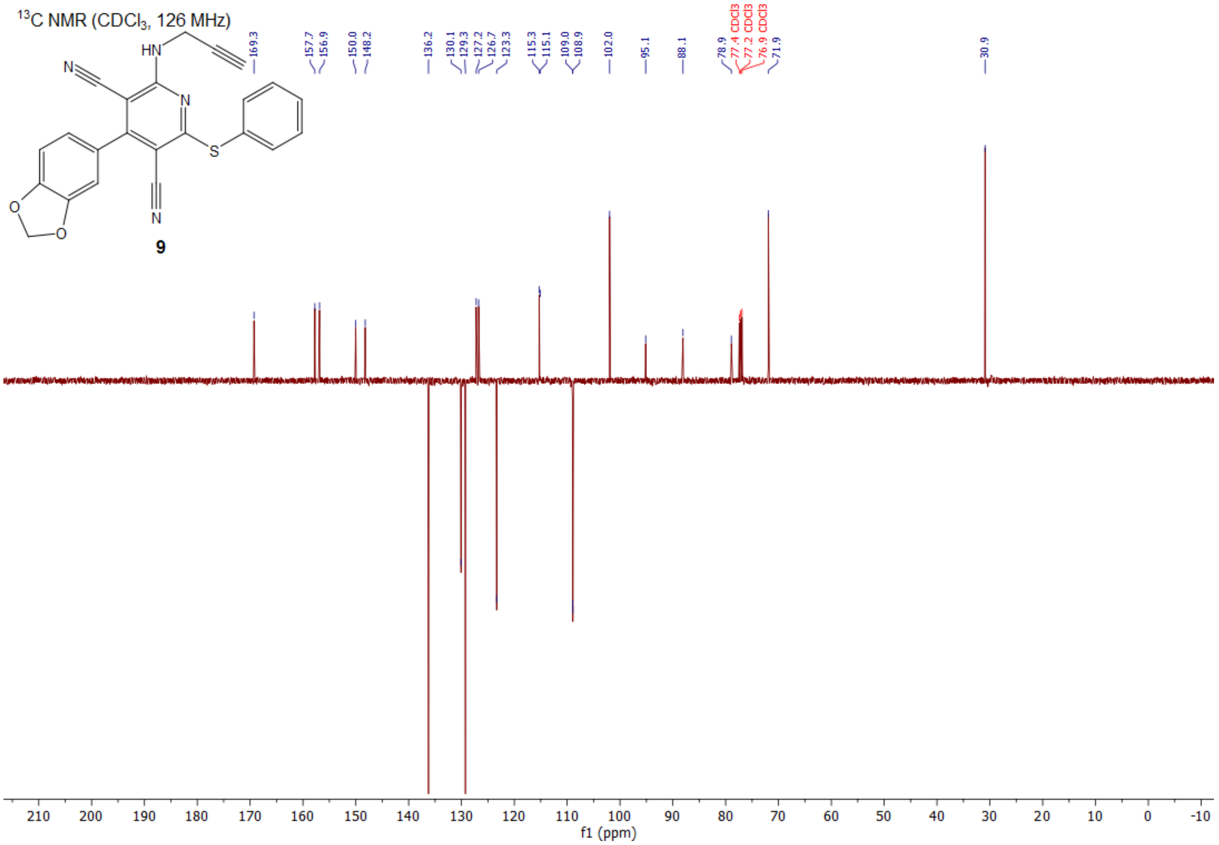

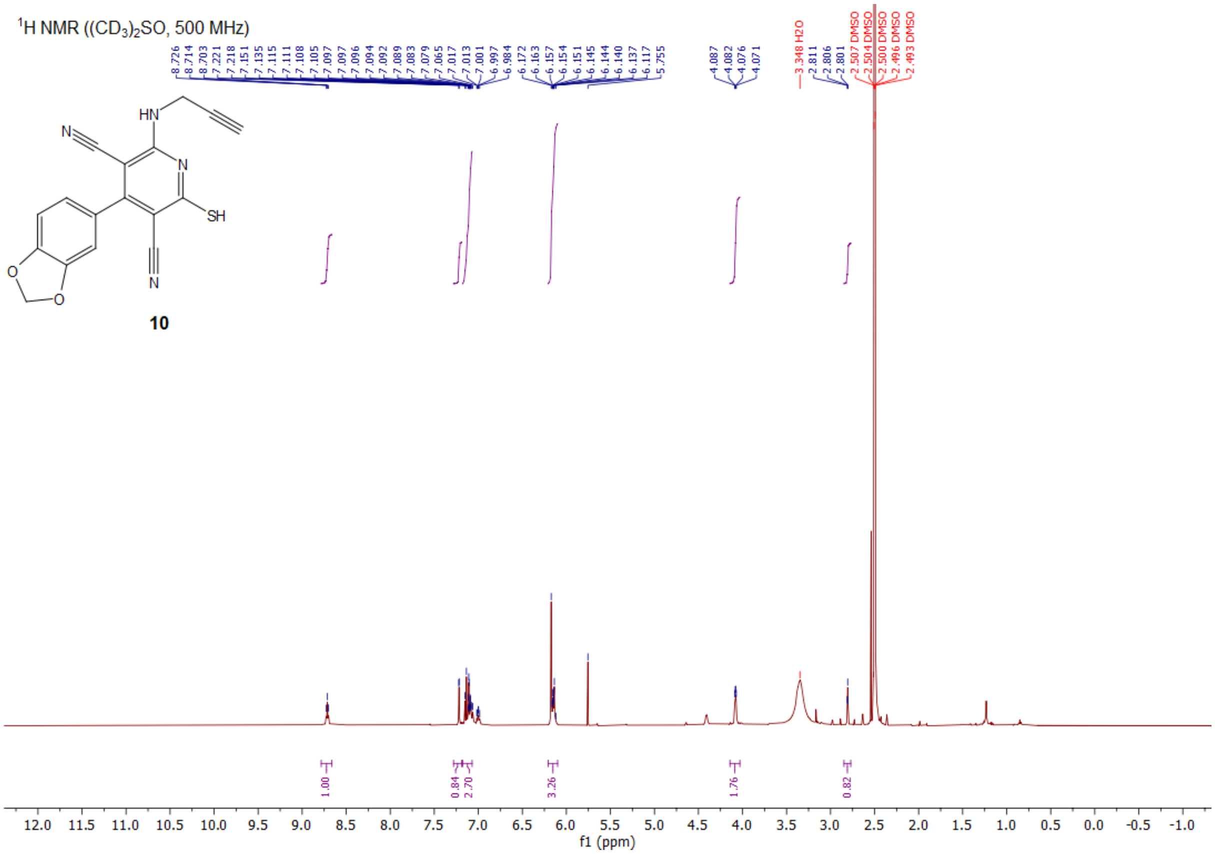

1H NMR ((CD3)2SO, 500 MHz)
10
f1 (ppm)

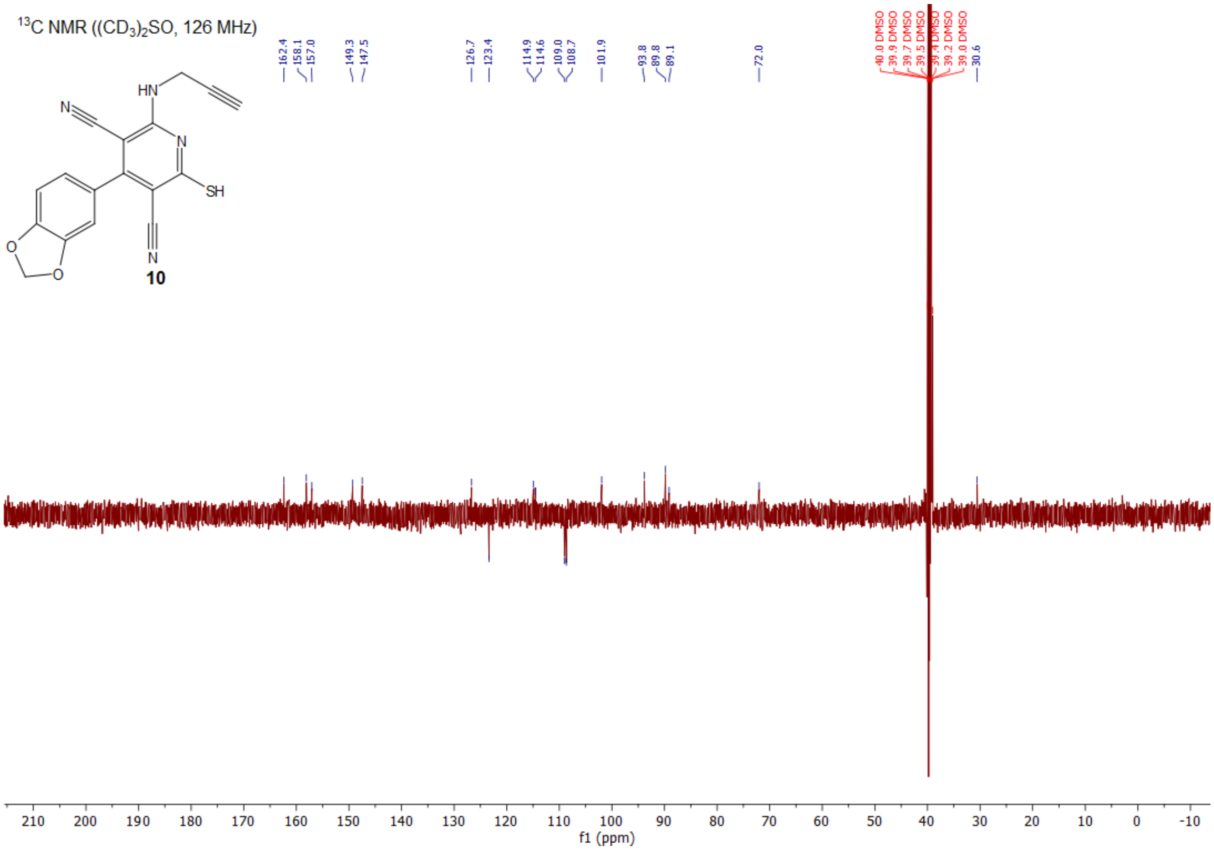

13C NMR ((CD3)2SO, 126 MHz)
10
f1 (ppm)

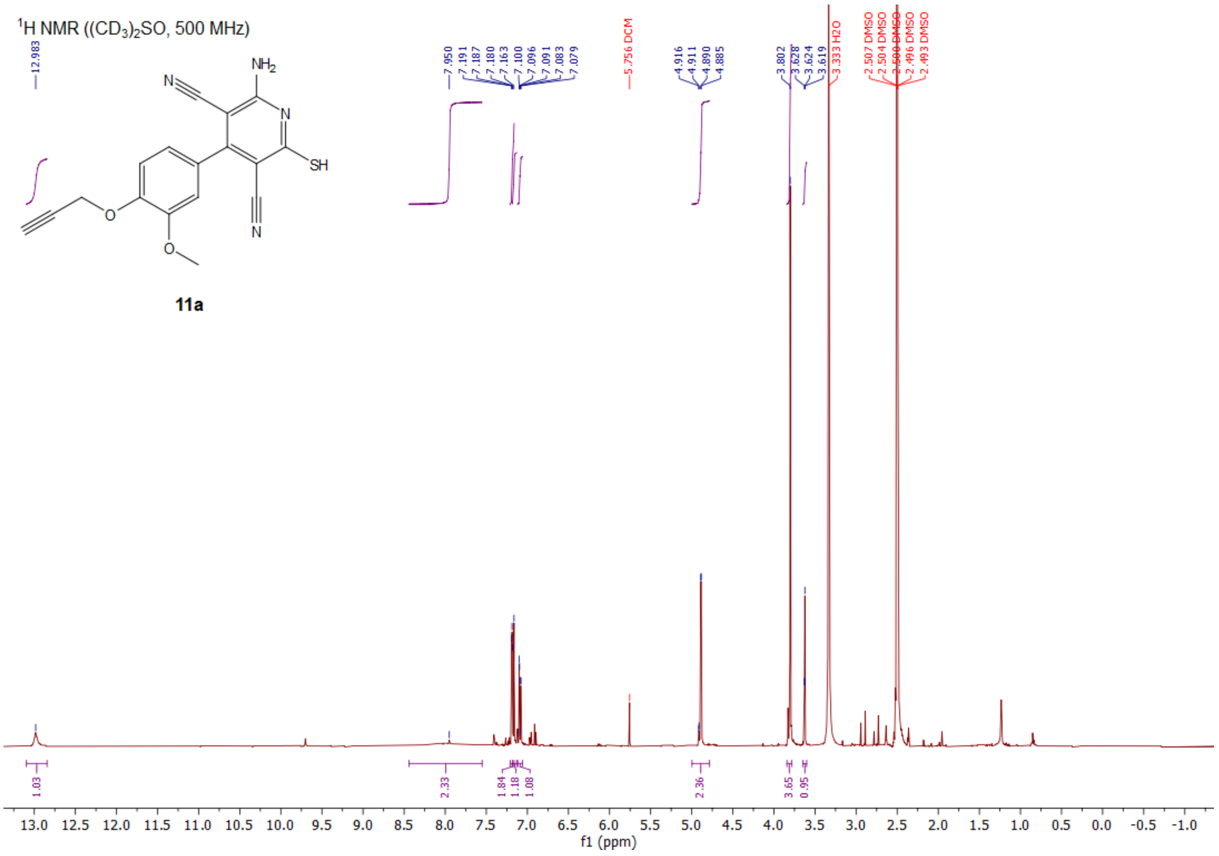

^{1}H NMR ((CD$_3$)$_2$SO, 500 MHz)
11a
12.983
7.950
7.191
7.187
7.180
7.163
7.100
7.096
7.091
7.083
7.079
5.756 DCM
4.916
4.911
4.890
4.885
3.802
3.628
3.624
3.619
3.333 H2O
2.507 DMSO
2.504 DMSO
2.500 DMSO
2.496 DMSO
2.493 DMSO
1.03
2.33
1.84
1.18
1.08
2.36
3.65
0.95
f1 (ppm)

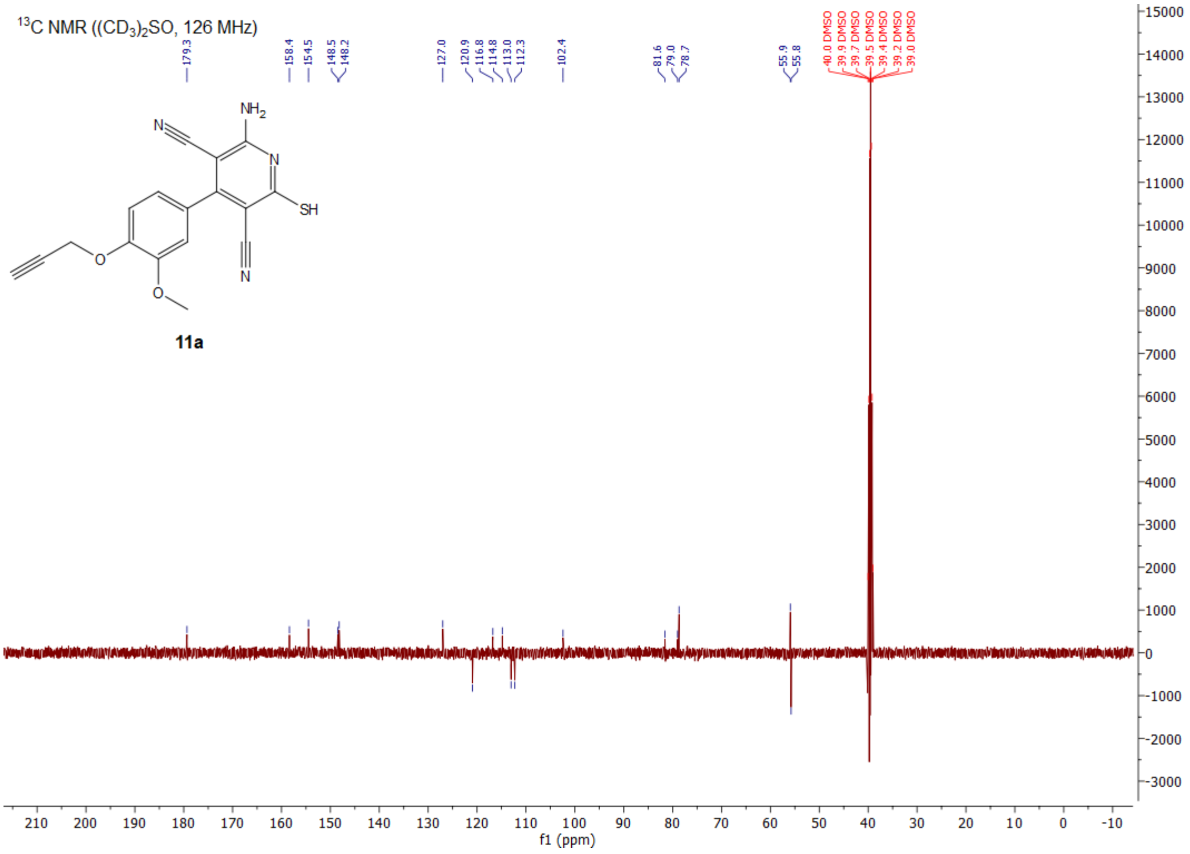

^{13}C NMR ((CD$_3$)$_2$SO, 126 MHz)
11a
179.3
158.4
154.5
148.5
148.2
127.0
120.9
116.8
114.8
113.0
112.3
102.4
81.6
79.0
78.7
55.9
55.8
40.0 DMSO
39.9 DMSO
39.7 DMSO
39.5 DMSO
39.4 DMSO
39.2 DMSO
39.0 DMSO
f1 (ppm)

^{1}H NMR (CD_3OD, 400 MHz)

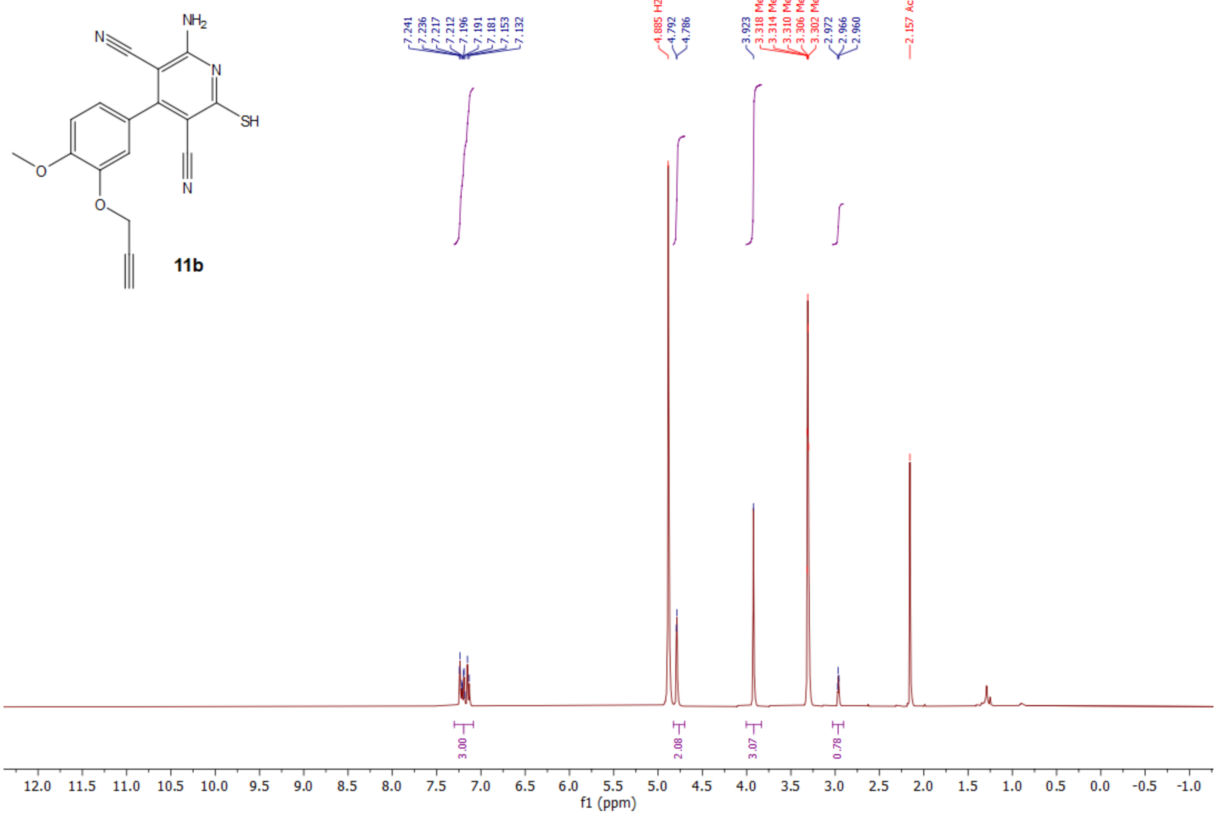

^{1}H NMR (CD_3OD, 400 MHz)

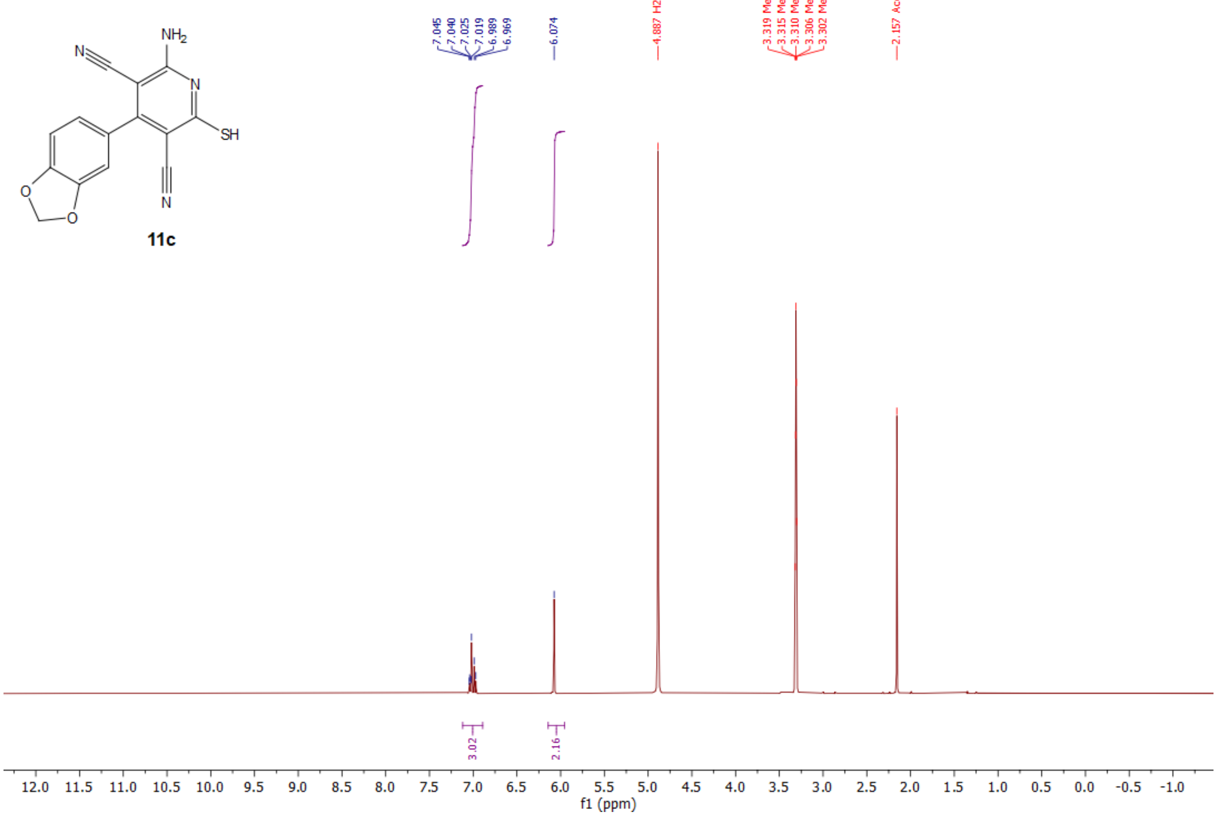

^{1}H NMR ($CDCl_3$, 300 MHz)

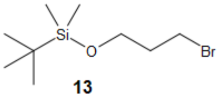

13

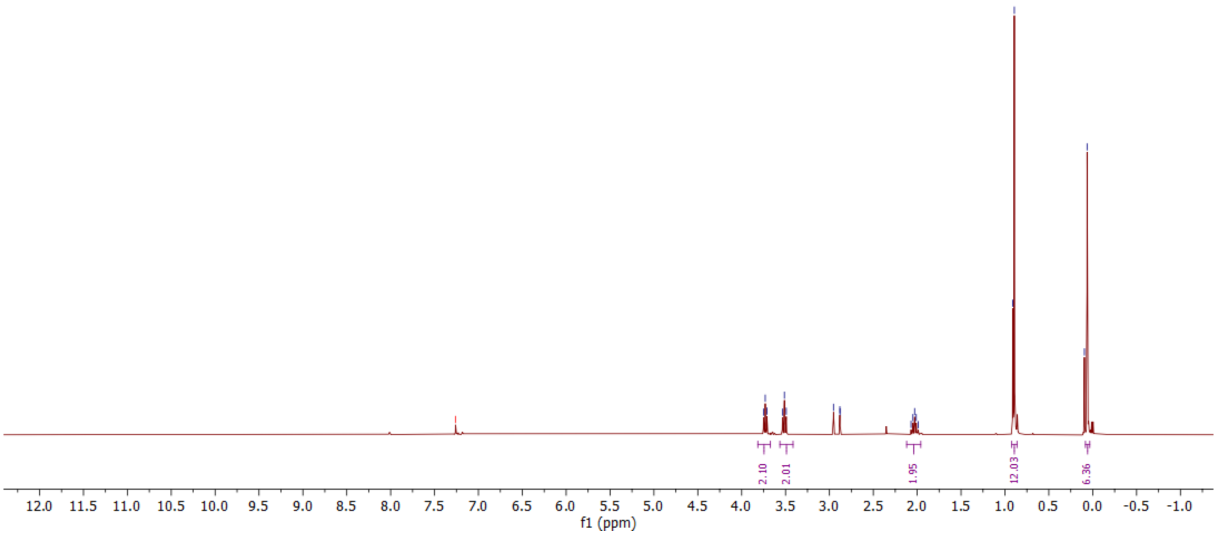

^{13}C NMR ($CDCl_3$, 75 MHz)

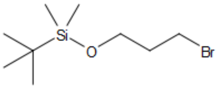

13

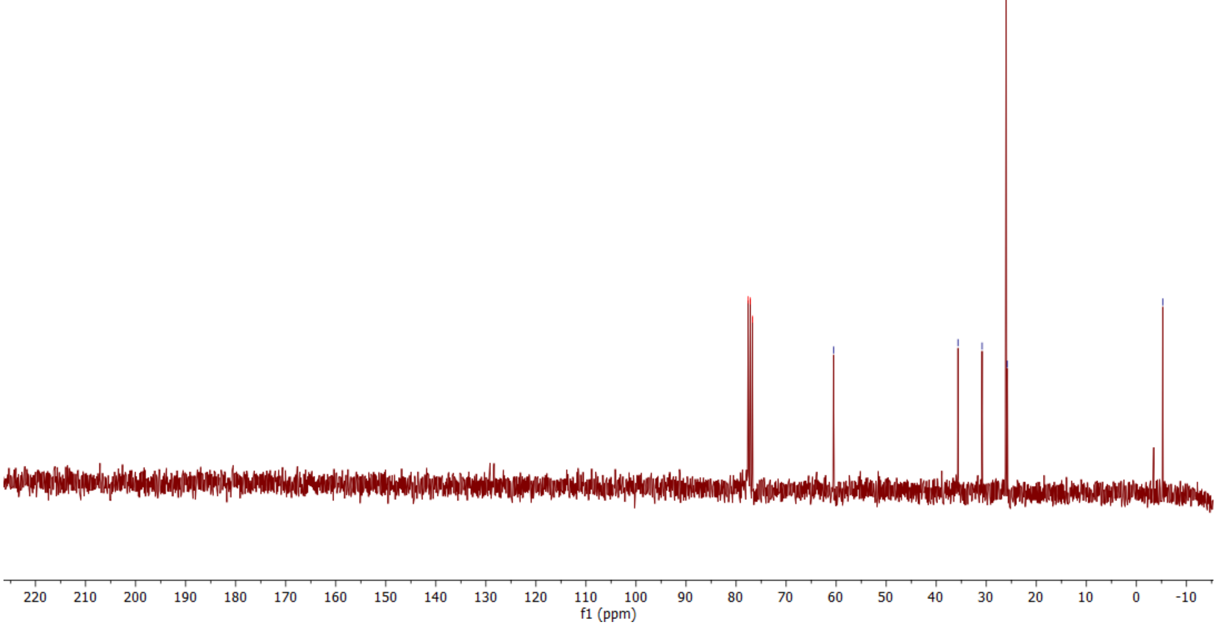

^{1}H NMR ($CDCl_3$, 400 MHz)

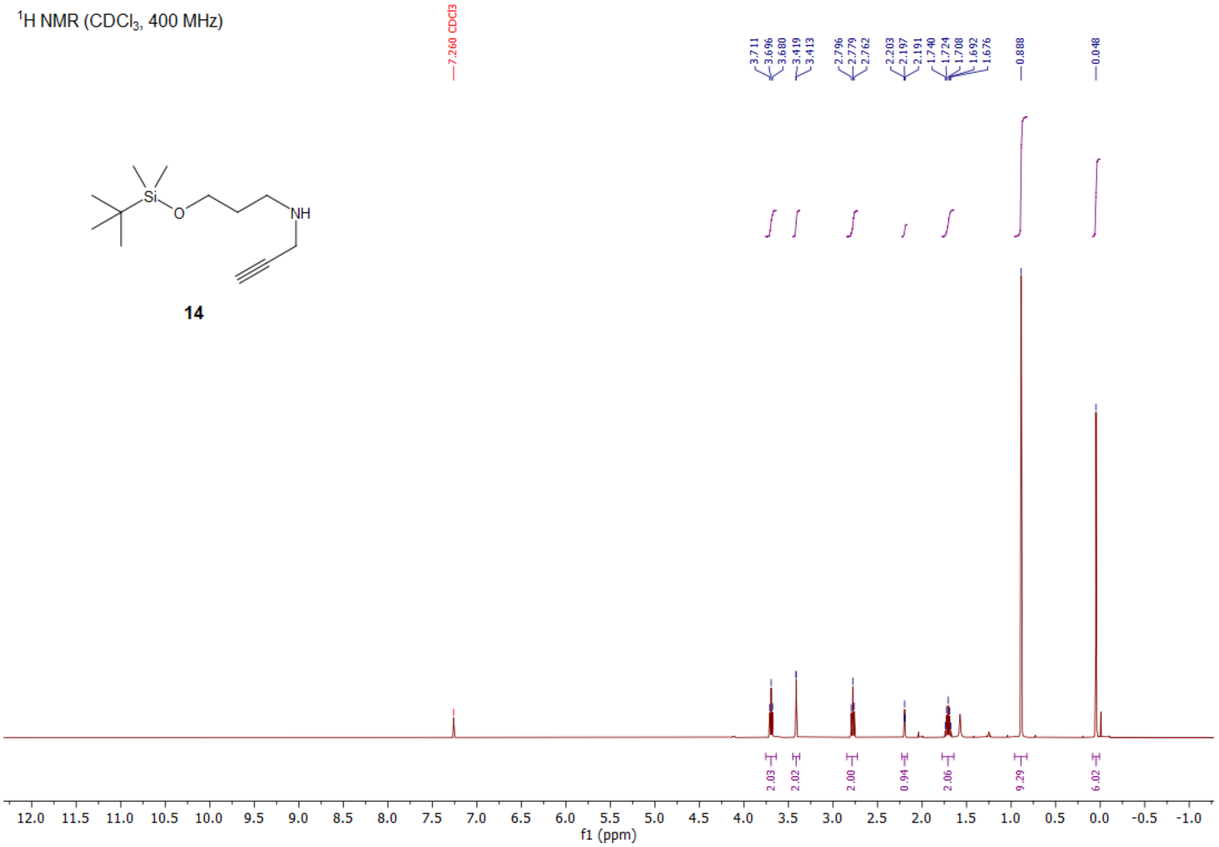

14

^{13}C NMR ($CDCl_3$, 101 MHz)

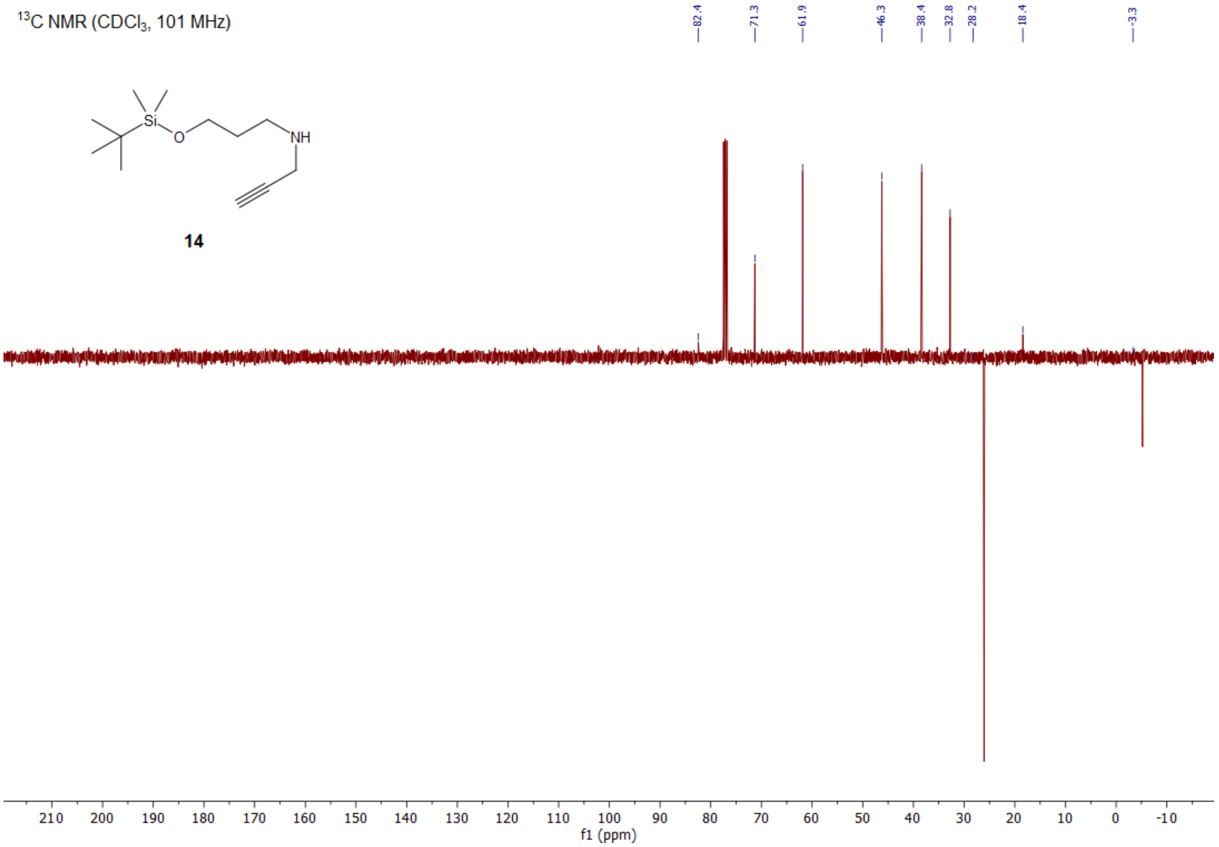

14

^{1}H NMR ($CDCl_3$, 500 MHz, 20 °C)

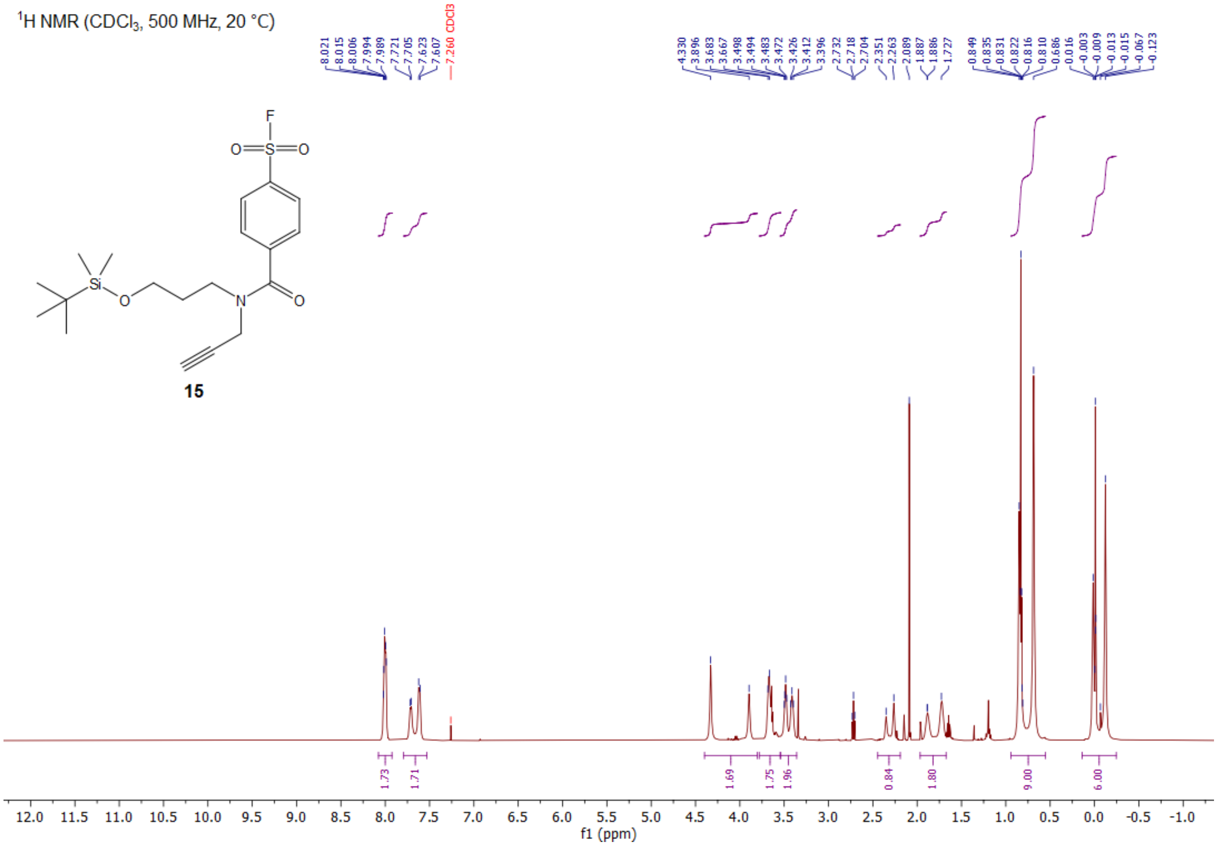

^{1}H NMR ($CDCl_3$, 500 MHz, 59 °C)

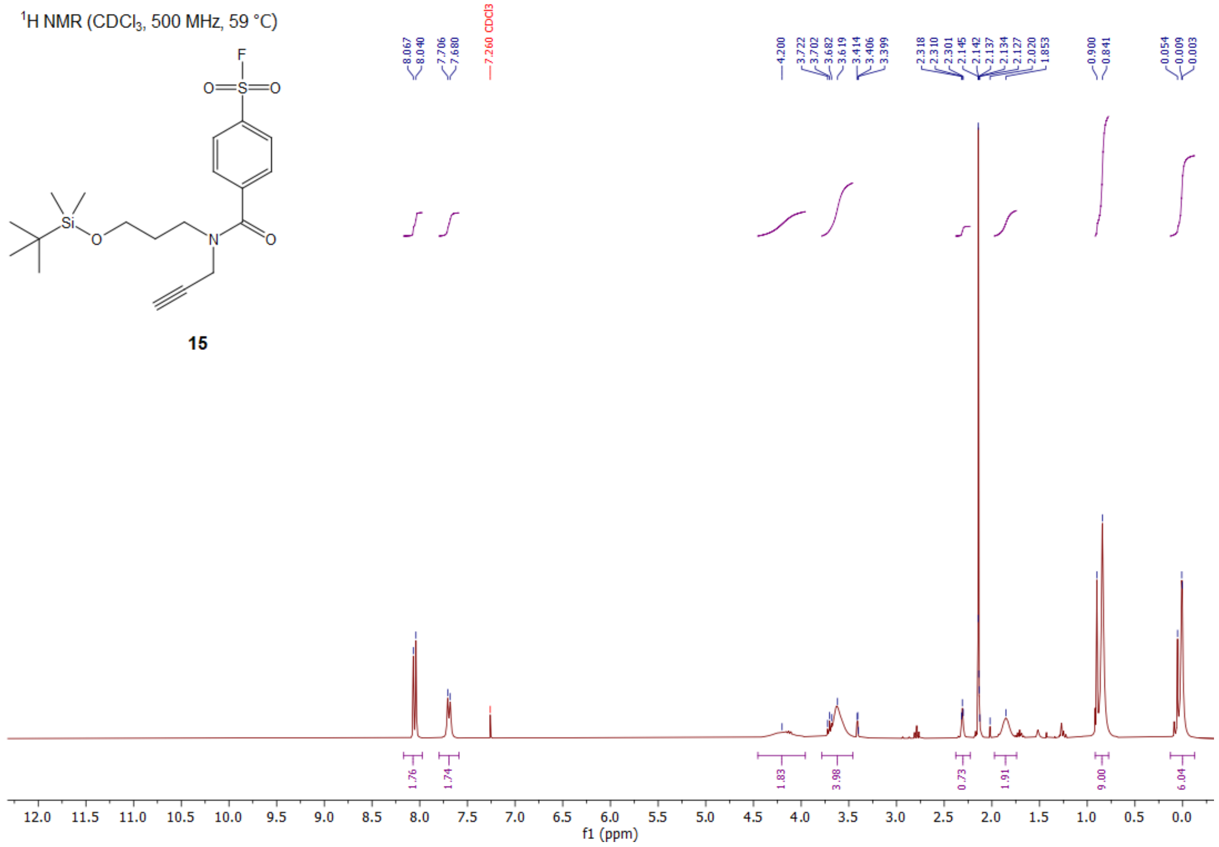

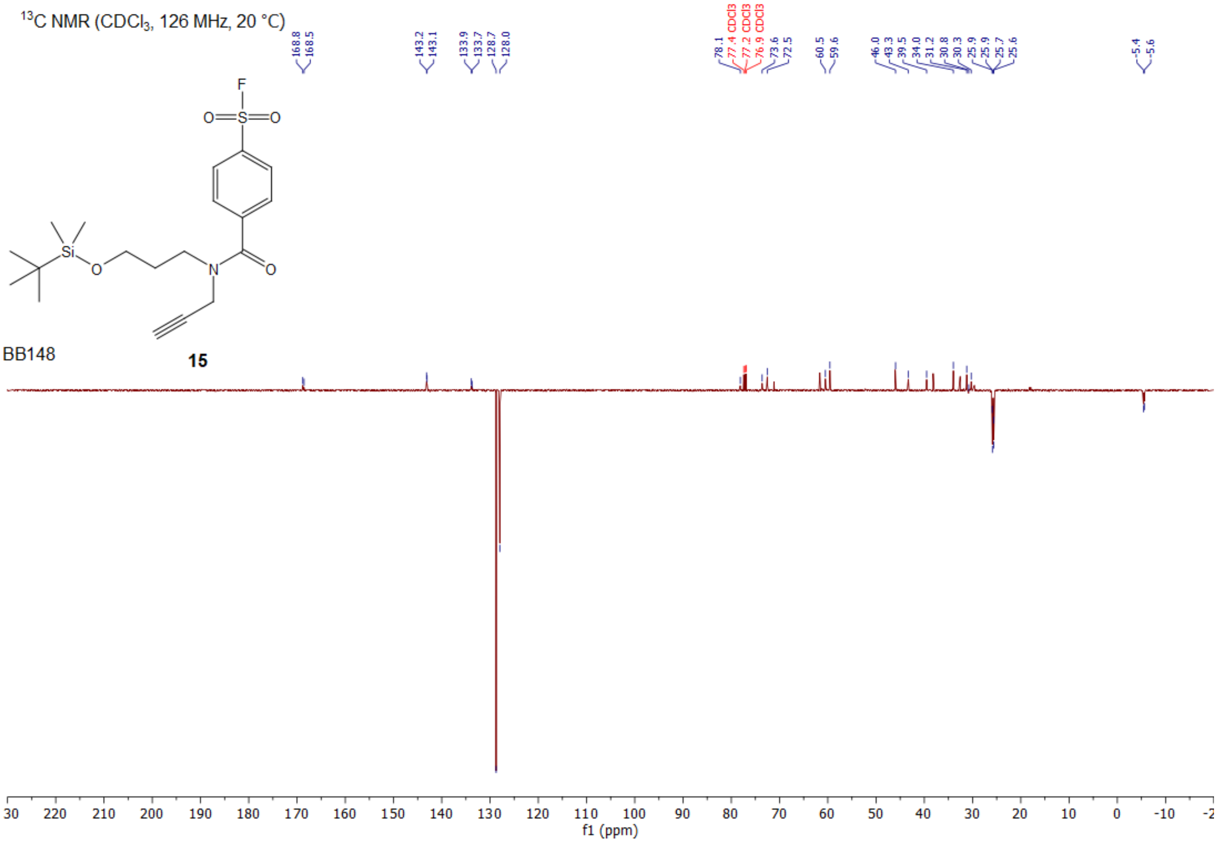
13C NMR (CDCl3, 126 MHz, 20 °C)
BB148
15
168.8
168.5
143.2
143.1
133.9
133.7
128.7
128.0
78.1
77.4 CDCl3
77.2 CDCl3
76.9 CDCl3
73.6
72.5
60.5
59.6
46.0
43.3
39.5
34.0
31.2
30.8
30.3
25.9
25.9
25.7
25.6
-5.4
-5.6
f1 (ppm)

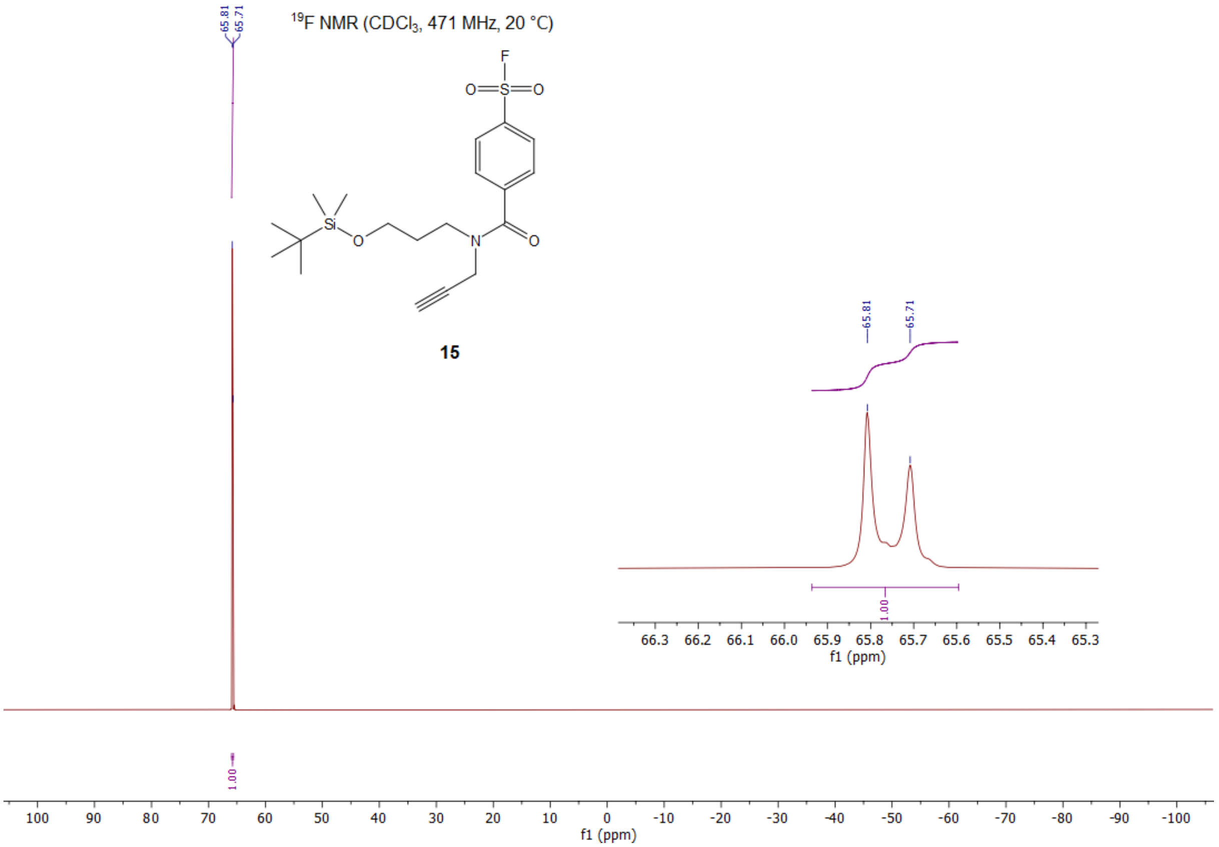
19F NMR (CDCl3, 471 MHz, 20 °C)
15
65.81
65.71
1.00
f1 (ppm)

^{1}H NMR ($CDCl_3$, 400 MHz)

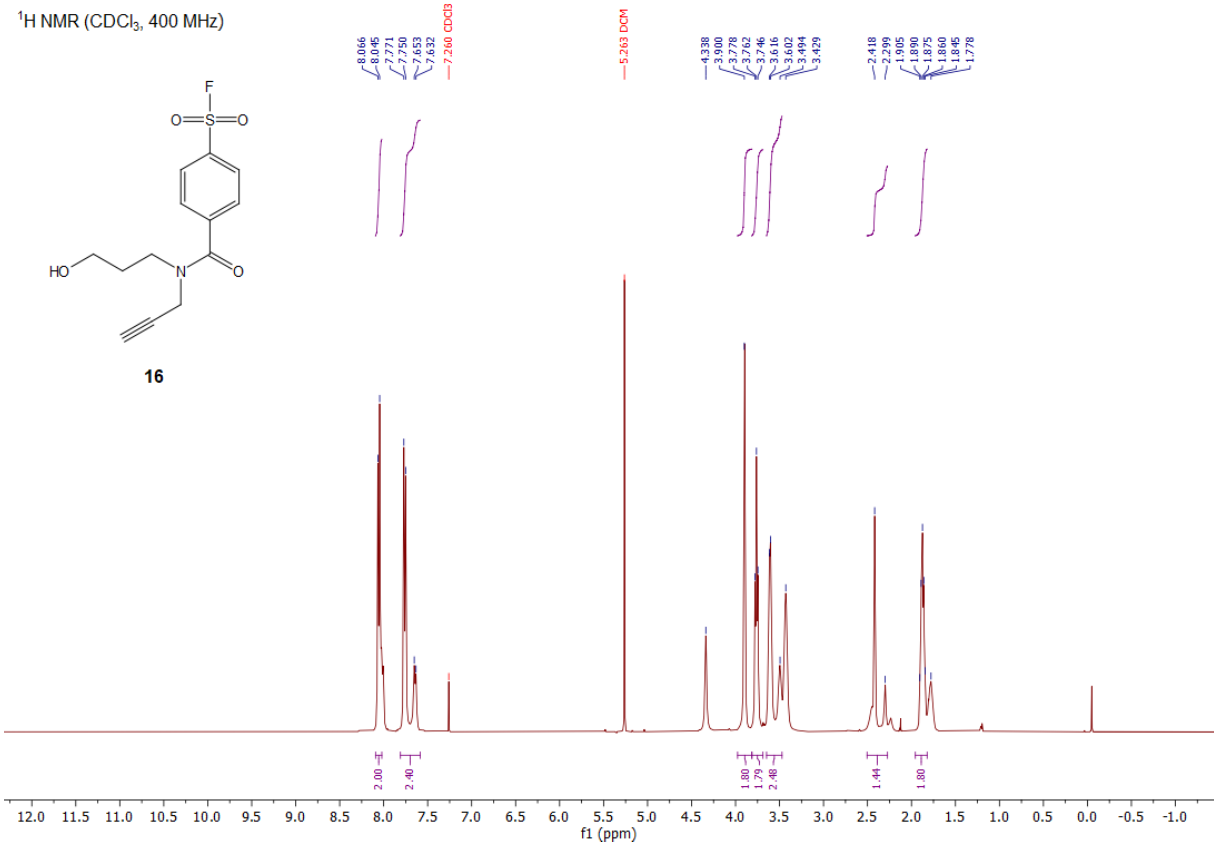

^{13}C NMR ($CDCl_3$, 101 MHz)

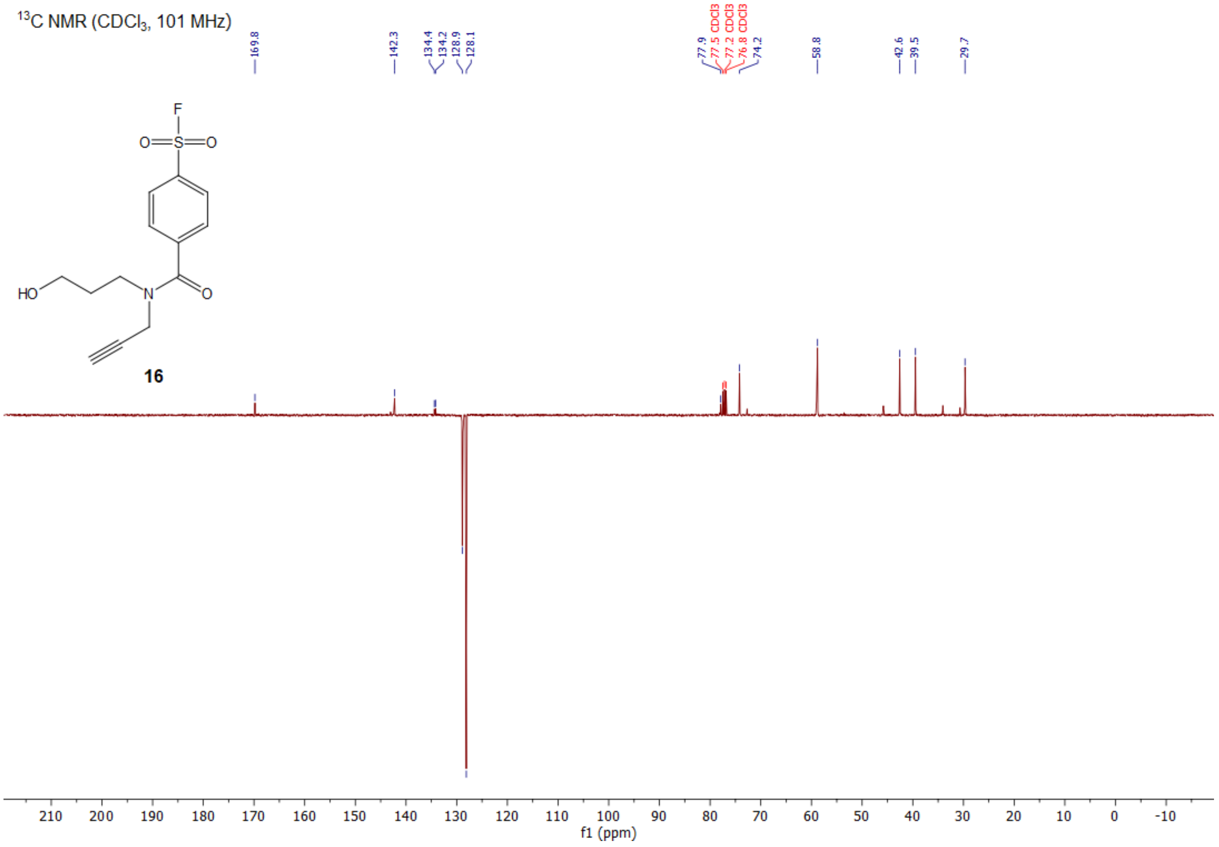

^{19}F NMR ($CDCl_3$, 471 MHz)

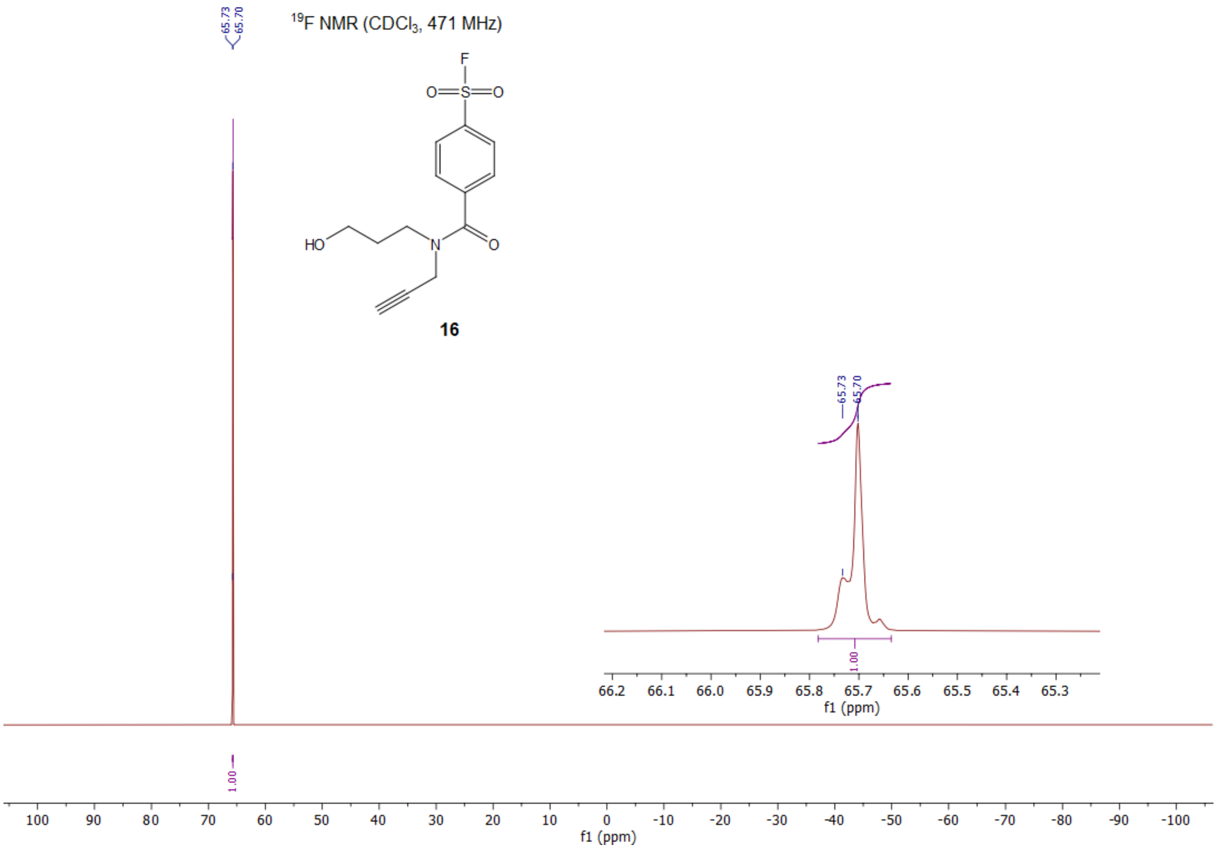

16

^{1}H NMR ($CDCl_3$, 500 MHz)

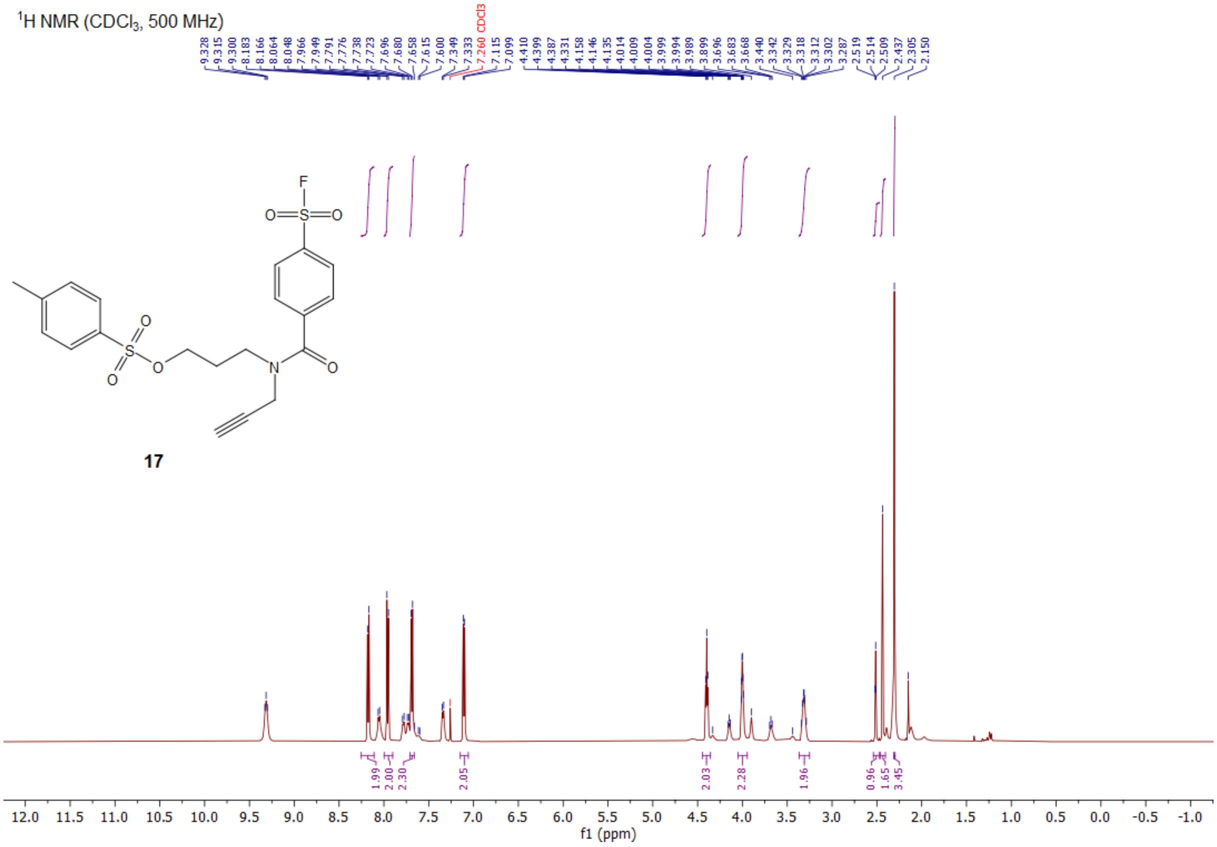

17

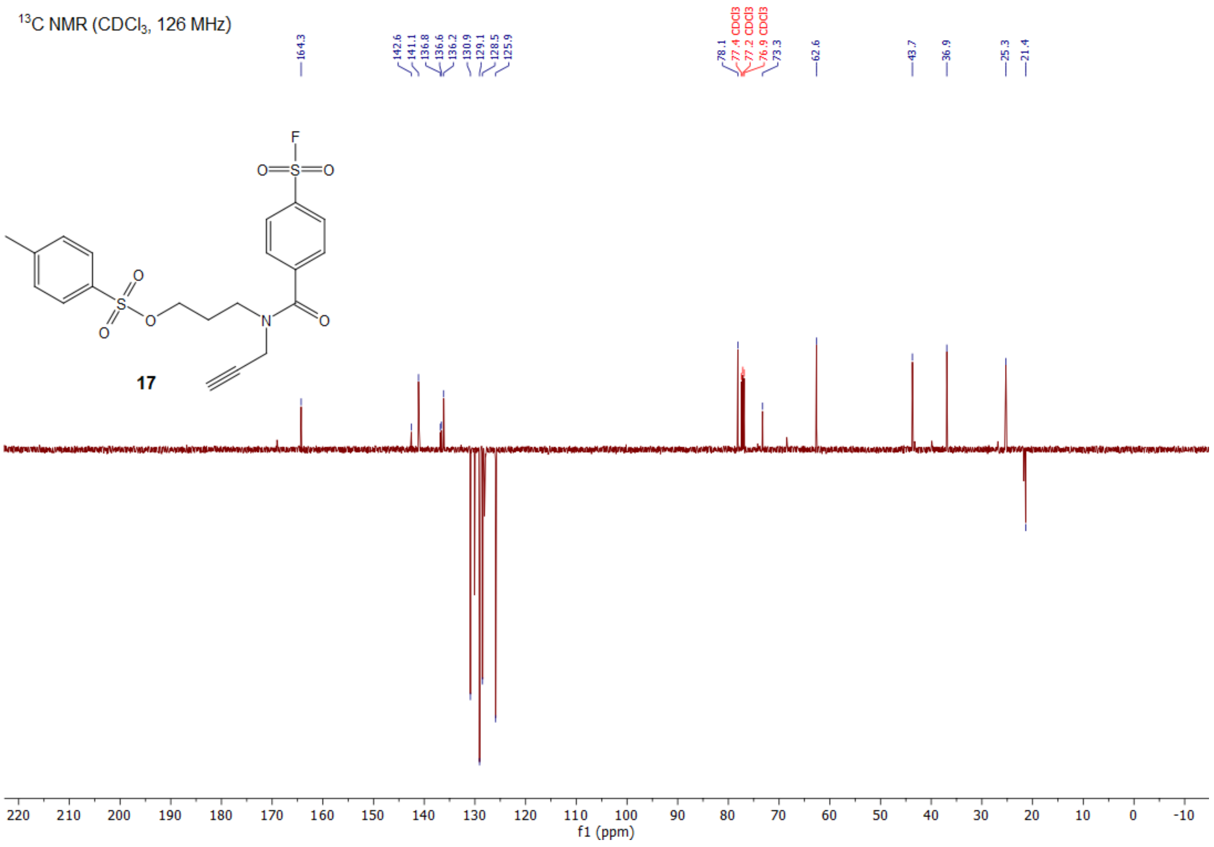

^{13}C NMR ($CDCl_3$, 126 MHz)
164.3
142.6
141.1
136.8
136.6
136.2
130.9
129.1
128.5
125.9
78.1
77.4 CDCl3
77.2 CDCl3
76.9 CDCl3
73.3
62.6
43.7
36.9
25.3
21.4
17
f1 (ppm)

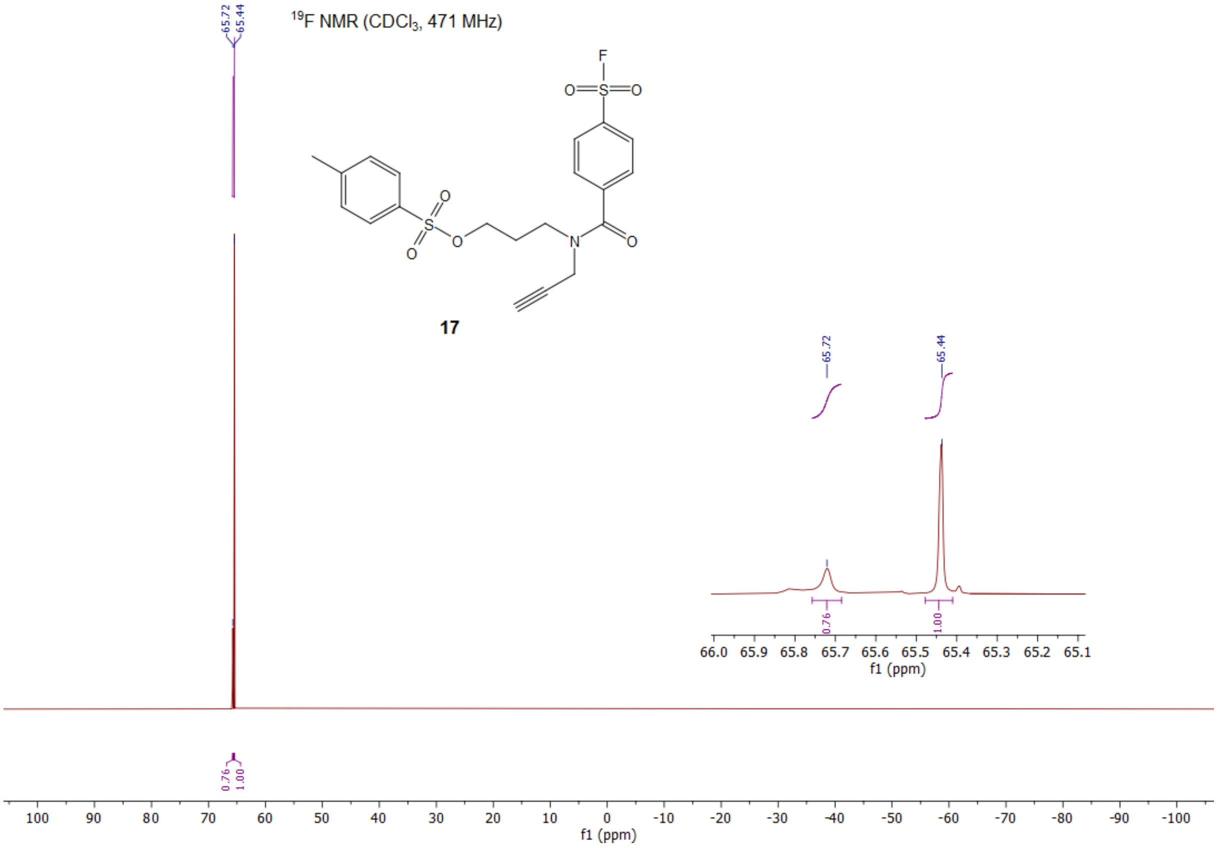

^{19}F NMR ($CDCl_3$, 471 MHz)
65.72
65.44
17
0.76
1.00
f1 (ppm)

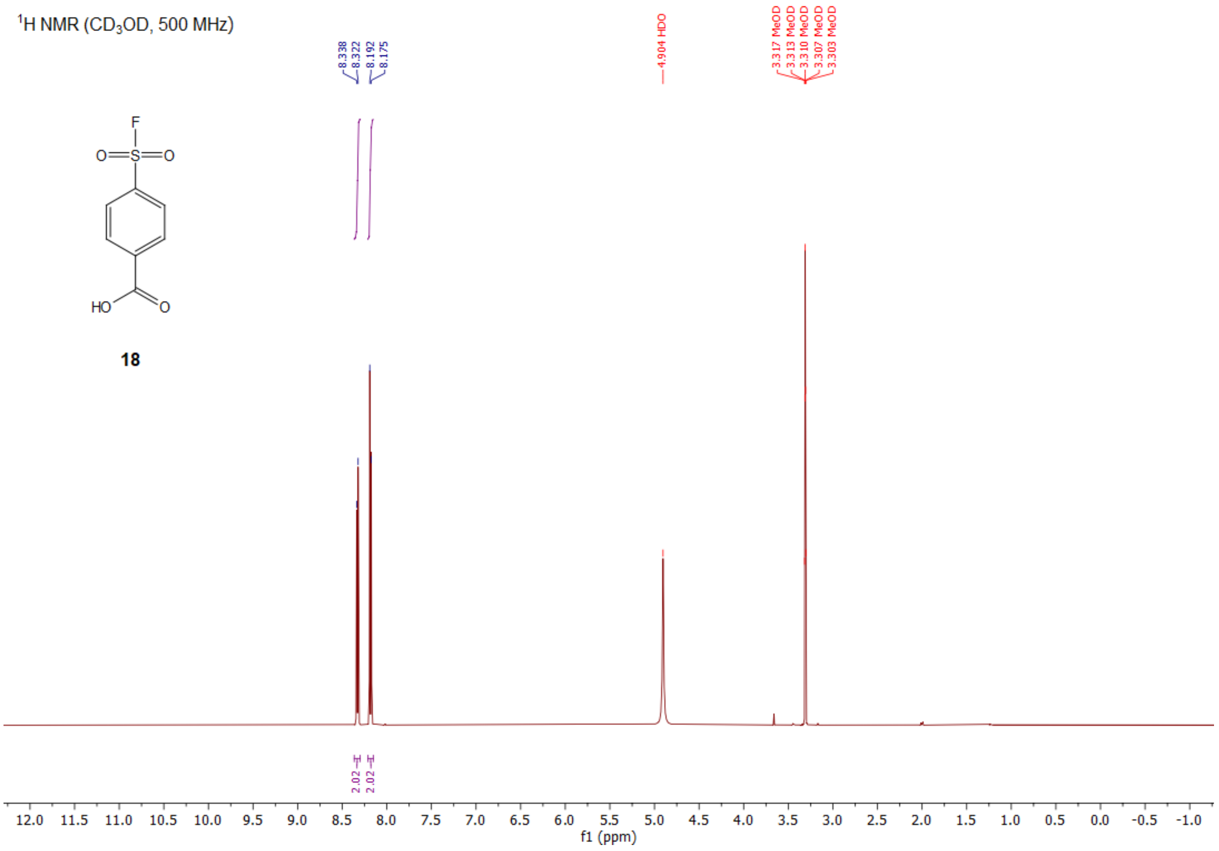

1H NMR (CD3OD, 500 MHz)
8.338
8.322
8.192
8.175
4.904 HDO
3.317 MeOD
3.313 MeOD
3.310 MeOD
3.307 MeOD
3.303 MeOD
F
O
S
O
HO
O
18
2.02
2.02
12.0
11.5
11.0
10.5
10.0
9.5
9.0
8.5
8.0
7.5
7.0
6.5
6.0
5.5
5.0
4.5
4.0
3.5
3.0
2.5
2.0
1.5
1.0
0.5
0.0
-0.5
-1.0
f1 (ppm)

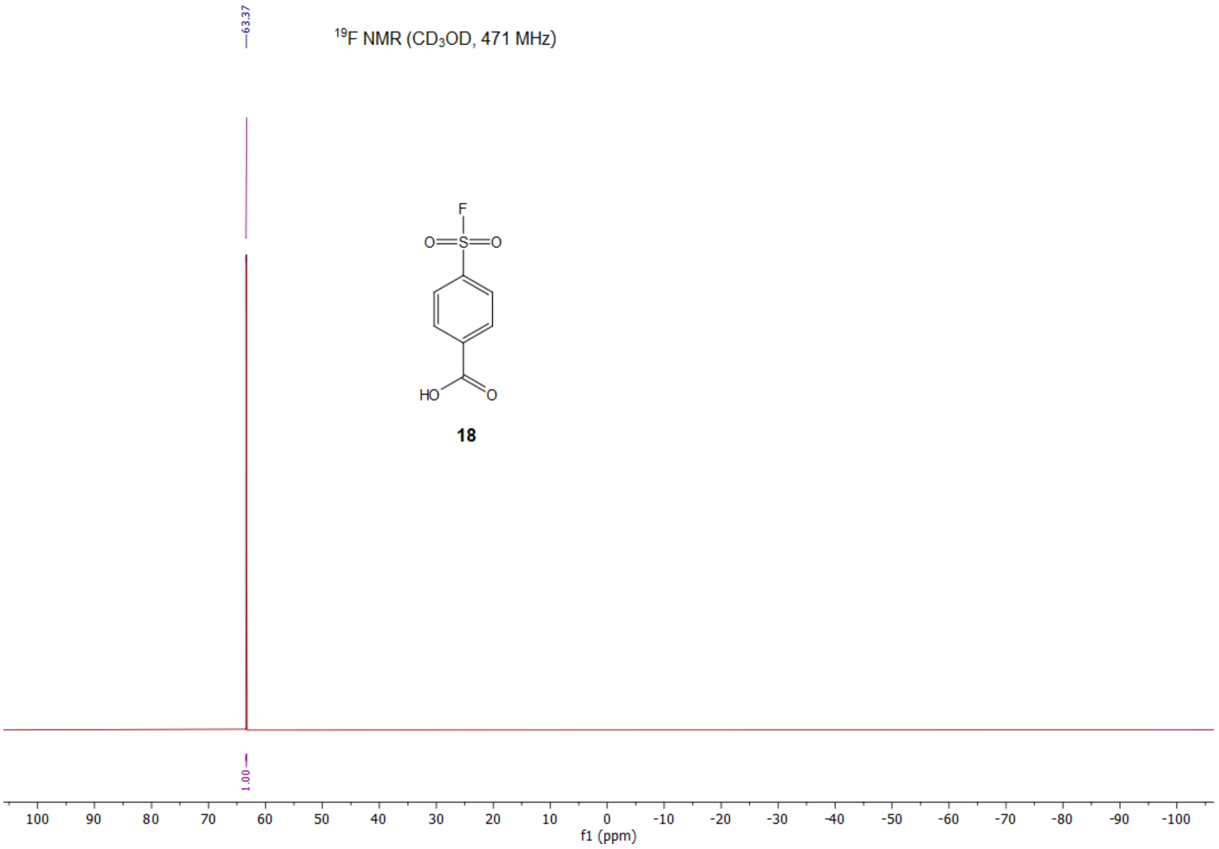

-63.37
19F NMR (CD3OD, 471 MHz)
F
O
S
O
HO
O
18
1.00
100
90
80
70
60
50
40
30
20
10
0
-10
-20
-30
-40
-50
-60
-70
-80
-90
-100
f1 (ppm)

^{1}H NMR ($CDCl_3$, 400 MHz)

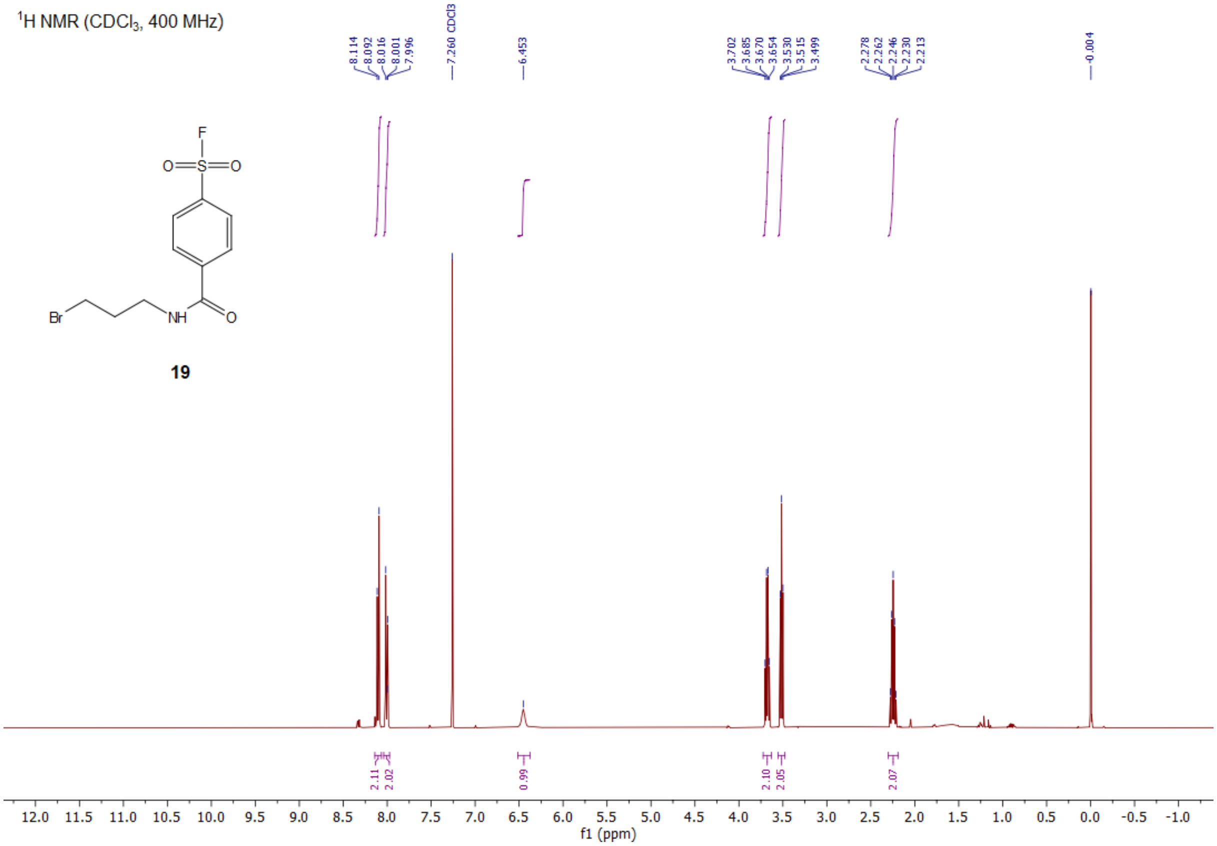

^{19}F NMR ($CDCl_3$, 471 MHz)

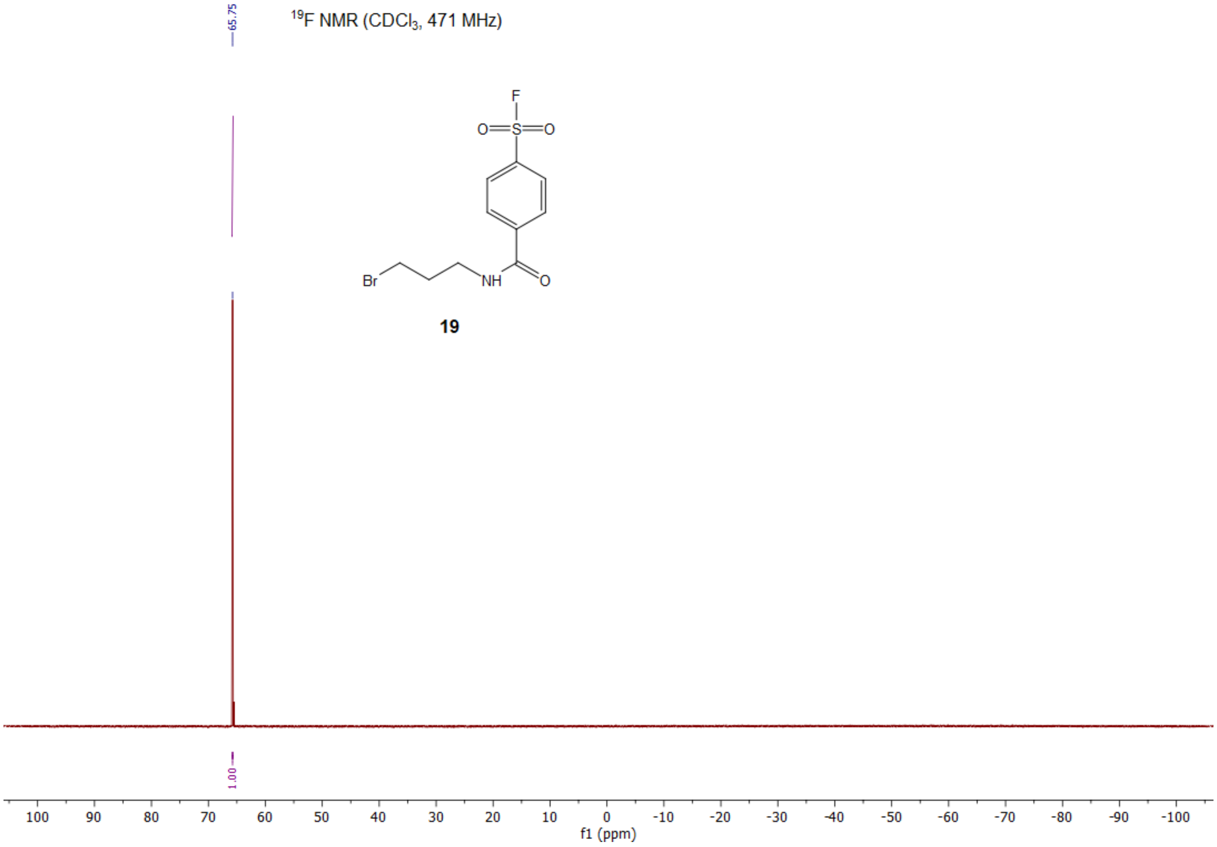

References

(1) Schrödinger Release 2019-1: Maestro, Schrödinger, LLC: New York, 2019.

(2) Zhu, K.; Borrelli, K. W.; Greenwood, J. R.; Day, T.; Abel, R.; Farid, R. S.; Harder, E. Docking Covalent Inhibitors: A Parameter Free Approach to Pose Prediction and Scoring. *J. Chem. Inf. Model.* **2014**, *54* (7), 1932–1940. https://doi.org/10.1021/ci500118s.

(3) Smith, P. K.; Krohn, R. I.; Hermanson, G. T.; Mallia, A. K.; Gartner, F. H.; Provenzano, M. D.; Fujimoto, E. K.; Goeke, N. M.; Olson, B. J.; Klenk, D. C. Measurement of Protein Using Bicinchoninic Acid. *Anal. Biochem.* **1985**, *150* (1), 76–85. https://doi.org/10.1016/0003-2697(85)90442-7.

(4) Rodbell, M. Metabolism of Isolated Fat Cells: I. EFFECTS OF HORMONES ON GLUCOSE METABOLISM AND LIPOLYSIS. *J. Biol. Chem.* **1964**, *239* (2), 375–380. https://doi.org/10.1016/S0021-9258(18)51687-2.

(5) McKeel, D. W.; Jarett, L. Preparation and Characterization of a Plasma Membrane Fraction from Isolated Fat Cells. *J. Cell Biol.* **1970**, *44* (2), 417–432. https://doi.org/10.1083/jcb.44.2.417.

(6) Liang, H. X.; Belardinelli, L.; Ozeck, M. J.; Shryock, J. C. Tonic Activity of the Rat Adipocyte A1-Adenosine Receptor. *Br. J. Pharmacol.* **2002**, *135* (6), 1457–1466. https://doi.org/10.1038/sj.bjp.0704586.

(7) Green, A.; Milligan, G.; Dobias, S. B. G(i) down-Regulation as a Mechanism for Heterologous Desensitization in Adipocytes. *J. Biol. Chem.* **1992**, *267* (5), 3223–3229. https://doi.org/10.1016/s0021-9258(19)50719-0.

(8) Yang, X.; Dilweg, M. A.; Osemwengie, D.; Burggraaff, L.; van der Es, D.; Heitman, L. H.; IJzerman, A. P. Design and Pharmacological Profile of a Novel Covalent Partial Agonist for the Adenosine A1 Receptor. *Biochem. Pharmacol.* **2020**, *180*. https://doi.org/10.1016/j.bcp.2020.114144.

(9) Cheng, Y.-C.; Prusoff, W. H. Relationship between the Inhibition Constant (KI) and the Concentration of Inhibitor Which Causes 50 per Cent Inhibition (I50) of an Enzymatic Reaction. *Biochem. Pharmacol.* **1973**, *22* (23), 3099–3108. https://doi.org/10.1016/0006-2952(73)90196-2.

(10) Kourounakis, A.; Visser, C.; de Groote, M.; IJzerman, A. P. Differential Effects of the Allosteric Enhancer (2-Amino-4,5-Dimethyl-Trienyl)[3-(Trifluoromethyl) Phenyl]Methanone (PD81,723) on Agonist and Antagonist Binding and Function at the Human Wild-Type and a Mutant (T277A) Adenosine A1 Receptor. *Biochem. Pharmacol.* **2001**, *61* (2), 137–144. https://doi.org/10.1016/S0006-2952(00)00536-0.

(11) van Rooden, E. J.; Florea, B. I.; Deng, H.; Baggelaar, M. P.; van Esbroeck, A. C. M.; Zhou, J.; Overkleeft, H. S.; van der Stelt, M. Mapping in Vivo Target Interaction Profiles of Covalent Inhibitors Using Chemical Proteomics with Label-Free Quantification. *Nat. Protoc.* **2018**, *13* (4), 752–767. https://doi.org/10.1038/nprot.2017.159.

(12) Wessel, D.; Flügge, U. I. A Method for the Quantitative Recovery of Protein in Dilute Solution in the Presence of Detergents and Lipids. *Anal. Biochem.* **1984**, *138* (1), 141–143.

https://doi.org/10.1016/0003-2697(84)90782-6.

(13) Rappsilber, J.; Mann, M.; Ishihama, Y. Protocol for Micro-Purification, Enrichment, Pre-Fractionation and Storage of Peptides for Proteomics Using StageTips. *Nat. Protoc.* **2007**, *2* (8), 1896–1906. https://doi.org/10.1038/nprot.2007.261.

(14) Cox, J.; Mann, M. MaxQuant Enables High Peptide Identification Rates, Individualized p.p.b.-Range Mass Accuracies and Proteome-Wide Protein Quantification. *Nat. Biotechnol.* **2008**, *26* (12), 1367–1372. https://doi.org/10.1038/nbt.1511.

(15) van Dalen, F. J.; Bakkum, T.; van Leeuwen, T.; Groenewold, M.; Deu, E.; Koster, A. J.; van Kasteren, S. I.; Verdoes, M. Application of a Highly Selective Cathepsin S Two-Step Activity-Based Probe in Multicolor Bio-Orthogonal Correlative Light-Electron Microscopy. *Front. Chem.* **2021**, *8*, 1–13. https://doi.org/10.3389/fchem.2020.628433.

(16) Allan, C.; Burel, J. M.; Moore, J.; Blackburn, C.; Linkert, M.; Loynton, S.; MacDonald, D.; Moore, W. J.; Neves, C.; Patterson, A.; Porter, M.; Tarkowska, A.; Loranger, B.; Avondo, J.; Lagerstedt, I.; Lianas, L.; Leo, S.; Hands, K.; Hay, R. T.; Patwardhan, A.; Best, C.; Kleywegt, G. J.; Zanetti, G.; Swedlow, J. R. OMERO: Flexible, Model-Driven Data Management for Experimental Biology. *Nat. Methods* **2012**, *9* (3), 245–253. https://doi.org/10.1038/nmeth.1896.
